# PIM1 controls GBP1 activity to limit self-damage and to guard against pathogen infection

**DOI:** 10.1126/science.adg2253

**Published:** 2023-10-06

**Authors:** Daniel Fisch, Moritz M Pfleiderer, Eleni Anastasakou, Gillian M Mackie, Fabian Wendt, Xiangyang Liu, Barbara Clough, Samuel Lara-Reyna, Vesela Encheva, Ambrosius P Snijders, Hironori Bando, Masahiro Yamamoto, Andrew D Beggs, Jason Mercer, Avinash R Shenoy, Bernd Wollscheid, Kendle M Maslowski, Wojtek P Galej, Eva-Maria Frickel

**Affiliations:** 1 Host-*Toxoplasma* Interaction Laboratory, The Francis Crick Institute, London, UK; 2 Institute of Microbiology and Infection, School of Biosciences, University of Birmingham, Edgbaston, UK; 3 European Molecular Biology Laboratory, 71 Avenue des Martyrs, Grenoble, France; 4 Institute of Immunology and Immunotherapy, University of Birmingham, Edgbaston, UK; 5 Department of Health Sciences and Technology (D-HEST), ETH Zurich, Institute of Translational Medicine (ITM), Zurich, Switzerland; 6 Swiss Institute of Bioinformatics (SIB), Lausanne, Switzerland; 7 Mass Spectrometry and Proteomics Platform, The Francis Crick Institute, London, UK; 8 Bruker Nederland BV; 9 Department of Immunoparasitology, Research Institute for Microbial Diseases, Osaka University, Osaka, Japan; 10 Laboratory of Immunoparasitology, WPI Immunology Frontier Research Center, Osaka University, Osaka, Japan; 11 Institute of Cancer and Genomic Sciences, University of Birmingham, Edgbaston, UK; 12 MRC Centre for Molecular Bacteriology & Infection, Department of Infectious Disease, Imperial College London, London, UK; 13 The Francis Crick Institute, London, UK; 14 Institute of Metabolism and Systems Research, University of Birmingham, Edgbaston, UK; 15 Cancer Research UK Beatson Institute, Glasgow, UK; 16 School of Cancer Sciences, University of Glasgow, Glasgow, UK

## Abstract

Disruption of cellular activities by pathogen virulence factors can trigger innate immune responses. Interferon-gamma (IFNγ)-inducible antimicrobial factors, such as the guanylate binding proteins (GBPs), promote cell-intrinsic defense by attacking intracellular pathogens and by inducing programmed cell death. Working in human macrophages, we discovered that GBP1-expression in the absence of IFNγ killed the cells and induced Golgi fragmentation. IFNγ-exposure improved macrophage survival via the activity of the kinase PIM1. PIM1 phosphorylated GBP1 leading to its sequestration by 14-3-3σ, which thereby prevented GBP1 membrane association. During *Toxoplasma gondii* infection, the virulence protein TgIST interfered with IFNγ-signaling and depleted PIM1 thereby increasing GBP1-activity. While infected cells can restrain pathogens in a GBP1-dependent manner, this mechanism can protect uninfected bystander cells. Thus, PIM1 can provide a bait for pathogen virulence factors, guarding the integrity of IFNγ-signaling.

IFNγ is a pivotal cytokine that promotes innate and adaptive immunity during infection. IFNγ acts on all nucleated cells to induce expression of a large repertoire of interferon-stimulated genes (ISGs) with potent antimicrobial activities (1). Pathogens are targeted by antimicrobial pathways after their detection by pattern recognition receptors (PRRs, pattern-triggered immunity) or when they disrupt a regulator of an immune response (effector-triggered immunity). The latter mechanism, also referred to as guard immunity, was originally described in plants and suggests that innate immunity can indirectly detect virulence factors via their activities (2). The complexity of guard immunity in mammalian systems remains largely unexplored (reviewed in (3)). Whether guard immunity exists in the context of inflammatory type II interferon production and signaling is unknown.

Among the ISGs are large GTPases of the GBP family (4). GBPs restrict infection by targeting intracellular microbes, promoting their rupture and release of microbial ligands, by redirecting autophagy and oxidative machinery to pathogen-containing compartments, and by removing replicative niches through induction of pyroptosis or apoptosis (5–15). IFNγ upregulates antimicrobial genes, including GBPs, in the primary infected cells and in neighboring, uninfected bystander cells. IFNγ thus induces ubiquitous expression of GBPs in most cell types. Among GBPs, GBP1 is the most abundant in IFNγ-primed cells. GBP1 has a C-terminal prenylation site that enables its membrane association (16) and forms homo-/hetero-dimers and membrane-bound coatomers with itself or with other GBPs (17, 18). The GBP1 GTPase converts GTP to GMP in two steps (19). GBP1 can target membranes of the host Golgi apparatus (16, 20), as well as plasma membrane-derived vacuoles containing *Toxoplasma gondii* (Tg) (10, 11) or lipopolysaccharide (LPS) on cytosolic bacteria (7, 11, 13–15). However, how uninfected cells protect themselves from the potentially destructive actions of GBP1 remains unclear.

## IFNγ and phosphorylation control GBP1 activity and prevent cytotoxicity

**Fig. 1:**
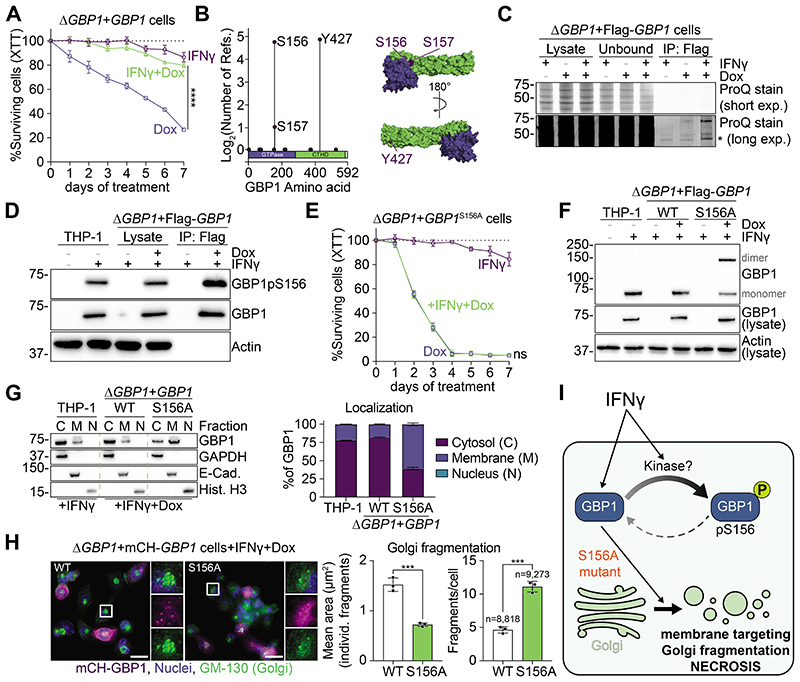
IFNγ and phosphorylation control GBP1 cytotoxicity and activity. **(A)** XTT cell survival assay of THP-1Δ*GBP1*+*GBP1* WT cells treated with IFNγ±Dox for indicated number of days. **(B)** Graph and GBP1 structure showing predicted phosphorylation sites and their surface-localization based on data from proteomics references (Ref). **(C)** Images with short or long exposure (exp.) for a ProQ-Diamond phosphoprotein stain for immunoprecipitated Flag-GBP1 from THP-1Δ*GBP1*+Flag-*GBP1* cells treated with IFNγ±Dox. *Marks background bands. **(D)** Immunoblots using GBP1pS156 antibody showing phosphorylation of GBP1 in IFNγ+Dox-treated THP-1Δ*GBP1*+Flag-*GBP1*. **(E)** XTT cell survival assay of THP-1Δ*GBP1*+*GBP1*^S156A^ cells treated with IFNγ±Dox for indicated number of days. **(F)** Immunoblot-analysis of GBP1 oligomerization in crosslinked pellets and in cell lysates. **(G)** Immunoblots of subcellular fractionation to determine GBP1 localization in IFNγ-primed THP-1 WT or Δ*GBP1*+*GBP1* cells expressing GBP1^S156A^ or WT GBP1 with markers for the cytosol (C), membranes (M) or the nucleus (N) and quantification. **(H)** Immunofluorescence images of cells expressing mCH-*GBP1* WT or S156A for 48 hours and stained for Golgi marker GM130 and quantification of mean area and number of Golgi fragments. Number of quantified cells indicated in figure. Green: GM-130 (Golgi); Magenta: mCH-GBP1; Blue: Nuclei; Scale bar 20 μm. **(I)** Cartoon summarizing findings on GBP1 activity control. **Data information:** Images in **(C-D)** and **(F-H)** representative of n = 3 experiments. Graphs in **(A)**, **(E)** and **(G+H)** show mean ± SEM from n = 3 experiments. *** *P* ≤ *0.001*; **** *P* < 0.0001 for indicated comparisons in **(A+E)** from one-way ANOVA and in **(H)** from unpaired t-tests; ns, not significant. For gel source data, see Data S5.

To investigate how the activities of GBP1 are regulated in uninfected cells, we used a Doxycycline (Dox)-inducible expression system in phorbol myristate acetate (PMA)-differentiated THP-1 macrophages (THP-1Δ*GBP1*+*GBP1*) (11). We first measured the survival of cells that ectopically expressed GBP1 in the absence of exposure to IFNγ. Dox-induced ectopic expression of GBP1 was cytotoxic, progressively leading to ~75% cell loss over 7 days (Fig. 1A). Exposure of GBP1-expressing cells to IFNγ reduced cell loss by ~55% (Fig. 1A). Treatment with IFNγ in the absence of ectopic GBP1 expression resulted in minimal cell loss (Fig. 1A). Thus, we surmised that an unknown IFNγ-dependent process and/or protein(s) must block GBP1-driven toxicity.

GBP1 contains three putative, surface-exposed phosphorylation sites: Ser156, Ser157 and Tyr427 (Fig. 1B). Immunoprecipitated Flag-GBP1 from macrophages probed for phosphorylated serine residues confirmed the presence of a phosphorylated GBP1 species, which increased upon exposure to IFNγ (Fig. 1C). We hypothesized that IFNγ-mediated phosphorylation of GBP1 could be a regulatory switch that suppresses the toxicity of GBP1. To test this possibility, we studied phosphorylation-deficient mutants of GBP1 (S156A, S157A, Y427F) expressed in Δ*GBP1* cells (Fig. S1). While S157A and Y427F had no effect on the dispersed, granular appearance of GBP1 within the cytosol, GBP1^S156A^ accumulated in perinuclear areas (Fig. S2A) that immunofluorescence assays revealed to be the Golgi apparatus (Fig. S2B). Furthermore, GBP1^S156A^-expressing cells were more elongated than cells expressing WT GBP1 (Fig. S2C) and displayed changes in their cortical actin cytoskeleton (Fig. S2D). Phosphorylation of GBP1 Ser156 might thus be controlling localization and activities of GBP1.

To test whether GBP1 was phosphorylated at Ser156 specifically, we raised a phospho-specific antibody and validated its specificity, as follows: First, the antibody specifically interacted with phosphorylated GBP1-peptide (epitope: amino acids Ile152-Cys166); second, it bound to immunoprecipitated Flag-GBP1 WT but not Flag-GBP1^S156A^; third, with the exception of minor reactivity towards GBP3, the antibody showed no cross-reactivity with other GBPs; and fourth, it specifically stained mCherry-tagged GBP1 (mCH-GBP1) but not mCH-GBP1^S156A^ in immunofluorescence assays (Fig. S3A-D). Using this antibody, we confirmed by immunoprecipitation and immunoblot assays that GBP1 was phosphorylated at Ser156 upon exposure of cells to IFNγ (Fig. 1D). Expression of GBP1^S156A^ was more toxic than WT GBP1 and IFNγ treatment no longer suppressed toxicity of GBP1^S156A^ (Fig. 1E). We thus checked macrophages that expressed WT GBP1 or GBP1^S156A^ for markers of regulated cell death (Fig. S4A) but did not observe any hallmarks of apoptosis, necroptosis or pyroptosis after 2, 4 or 7 days (Fig. S4B). Chemical inhibition of regulated cell death pathways failed to prevent cells from dying over 4 days of constitutive ectopic expression of GBP1 (Fig. S4C). Again, addition of IFNγ rescued cells that expressed WT GBP1 but not GBP1^S156A^ and minimally affected cell viability by itself (Fig. S4C). Thus, GBP1 phosphorylation on Ser156 was required to prevent unwanted toxicity and unregulated, necrotic death of human macrophages.

We next asked whether phosphorylation of GBP1 on Ser156 affected its known activities, such as oligomerization (14, 17, 18) and membrane association (16). Immunoblots showed that cytosolic WT GBP1 was monomeric, whereas GBP1^S156A^ dimers were readily detected, suggestive of increased GBP1 activity (Fig. 1F). Subcellular fractionation showed increased membrane association of GBP1^S156A^ (Fig. 1G). Because GBP1^S156A^ also exhibited increased Golgi targeting (Fig. S2B), we next assessed Golgi morphology after 2 days of continuous GBP1 expression. We saw increased fragmentation of the Golgi in cells that expressed GBP1^S156A^ as compared to WT GBP1, showing ~2.5x more fragments per cell with a decreased average fragment size (Fig. 1H). Taken together, the phosphodeficient GBP1^S156A^ mutant showed increased association with and disruption of the Golgi, which was accompanied by unregulated cellular necrosis. Thus, Ser156 phosphorylation suppresses GBP1 activities and protects cells from self-inflicted damage (Fig. 1I).

## The kinase PIM1 phosphorylates GBP1 at Ser156

**Fig. 2:**
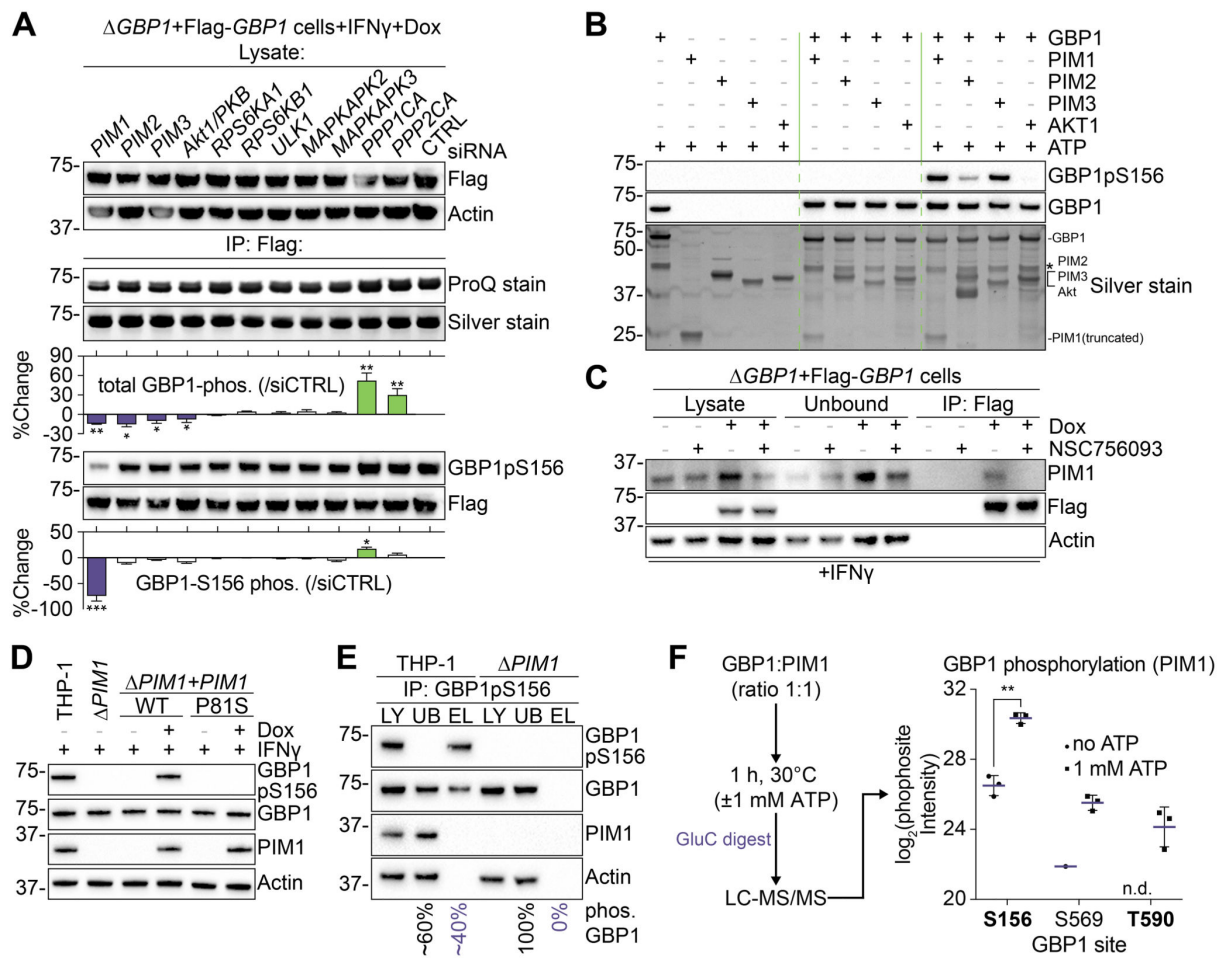
The kinase PIM1 phosphorylates GBP1 at Ser156. **(A)** Immunoblots and silver/ProQ-Diamond phosphoprotein stain of immunoprecipitated Flag-GBP1 WT from IFNγ+Dox-treated THP-1Δ*GBP1*+Flag-*GBP1* cells transfected with siRNA for the indicated genes. Quantification shows change in total GBP1 phosphorylation (top) and change in GBP1 Ser156 phosphorylation (bottom) normalized to siRNA CTRL transfected cells. **(B)** Immunoblot and silver stain of in vitro kinase assays with PIM family kinases ±10 μM ATP. *Marks unspecific protein bands. **(C)** Immunoblots following crosslinking and immunoprecipitation of Flag-GBP1 from THP-1Δ*GBP1*+Flag-*GBP1* cells treated with IFNγ±Dox and GBP1:PIM1 interaction inhibitor NSC756093. **(D)** Immunoblots testing for phosphorylation of endogenous GBP1 in IFNγ-primed THP-1 WT or Δ*PIM1* macrophages reconstituted with PIM1 WT or kinase dead (P81S) and additionally treated with Dox. **(E)** Immunoblots of GBP1-immunoprecipitation from IFNγ-primed THP-1 WT or Δ*PIM1* cells using GBP1pS156 antibody. Volumes of unbound (UB) and elution (EL) fraction were equalized to determine the proportion of phosphorylated GBP1 (indicated below). LY: lysate. **(F)** Mass spectrometry analysis of the PIM1:GBP1 kinase assay. Plot depicts log2 phosphosite intensity of identified Ser156, Ser569, Thr590 sites of GBP1, which were localized with probabilities > 0.99. **Data information:** Images in **(A-E)** representative of n = 3 experiments. Graphs in **(A)** show mean ± SD from n = 3 experiments, and in **(F)** mean ± SD from n = 3 replicates. * *P* ≤*0.05*; ** *P* ≤ *0.01*; *** *P* ≤ *0.001*; **** *P* ≤ *0.0001* in **(A)** from one-way ANOVA comparing to siRNA CTRL transfected cells and in **(F)** from unpaired two sample t-test following adjustment for multiple comparisons. For gel source data, see Data S5.

To narrow down the number of kinases capable of phosphorylating GBP1 on Ser156, we first used in silico analysis, which identified 9 candidate protein kinases (Fig. S5A). For each of these kinases, we confirmed expression in macrophages by RT-qPCR and reduced their expression via siRNA silencing (Fig. S5B and Fig. S6). Next, we immunoprecipitated Flag-GBP1 from THP-1 cells transfected with siRNAs directed against the kinases or against protein phosphatase 1/2 catalytic subunit alpha (*PPP1CA, PPP2CA;* Fig. 2A). Quantification of phosphoproteins suggested a reduction in total phosphorylation of GBP1 when PIM-family kinases were silenced, whereas silencing of *PPP1CA* or *PPP2CA* increased its total phosphorylation levels (Fig. 2A). Immunoblots using the Ser156 phospho-specific antibody showed that loss of PIM1 most strongly reduced phosphorylation of GBP1 at this residue (Fig. 2A). We next tested whether PIM family kinases could directly phosphorylate GBP1. Indeed, recombinant PIM1, 2 or 3 all phosphorylated GBP1 to varying degrees in vitro (Fig. 2B). Furthermore, we confirmed a previously observed direct interaction between PIM1 and GBP1 (21) in IFNγ-exposed THP-1 cells following cross-linking and immunoprecipitation. The PIM1:GBP1 interaction inhibitor NSC756093 abolished this interaction (Fig. 2C). Next, we generated THP-1Δ*PIM1* CRISPR/Cas9 knockout cells and confirmed the knockout of PIM1 by immunoblotting and RT-qPCR (Fig. S7A). Endogenous GBP1 was not phosphorylated at Ser156 in IFNγ-exposed Δ*PIM1* cells and phosphorylation was restored upon reconstitution with PIM1 WT but not with a PIM1 kinase-dead mutant (PIM1^P81S^ (22); Fig. 2D and Fig. S7B). Immunoprecipitation of endogenous GBP1 from IFNγ-exposed THP-1 WT cells with the phospho-specific antibody showed that ~40% of the cellular GBP1 pool was phosphorylated (Fig. 2E).

PIM1/2/3 are >65% similar to each other but vary in their efficiency of GBP1 phosphorylation (Fig. 2A+B). To assess whether PIM2/3 also play a role in cellulo, we compared their expression levels in naïve and IFNγ-treated macrophages by RT-qPCR and immunoblotting. Only PIM1 showed IFNγ-inducibility (Fig. S8A), confirming previous findings (23). Knockout of PIM1 did not influence the kinetics or magnitude of GBP1 expression (Fig. S8B), but endogenous GBP1 was no longer phosphorylated at Ser156 in Δ*PIM1* cells (Fig. 2D+S8C). Additional depletion of PIM2/3 in Δ*PIM1* cells had no influence on phosphorylation of GBP1 as determined by immunoblotting (Fig. S8C). However, immunoprecipitation and ProQ-stain of endogenous GBP1 from Δ*PIM1* cells showed that the protein remained phosphorylated at residues other than Ser156 (Fig. S8D).

Because PIM1 showed a prominent effect on phosphorylation of GBP1 and is a known GBP1-interactor in cancer cells, we performed additional experiments with recombinant PIM1 and GBP1 to explore their interaction. These experiments showed a concentration-dependent increase in phosphorylation of GBP1 by PIM1, which was abolished by inclusion of NSC756093, corroborating a requirement for direct interaction between the two proteins (Fig. S8E). High resolution mass spectrometry enabled dissection of the PIM1 kinase-GBP1 substrate relationship and identified three phosphorylation sites (Ser156, Ser569, Thr590; Fig. 2F). Fragment peak annotation of the electron-transfer dissociation (ETD)-activated MS/MS spectra for the corresponding monophosphorylated peptides unequivocally confirmed phosphorylation at Ser156 and Thr590 (MaxQuant PTM site localization probability >0.9999; Fig. S8F and Data S1). Ser156-phosphorylation increased by 14-fold upon addition of ATP, firmly establishing PIM1 as a bona fide GBP1 kinase.

We next sought to identify the PIM1 recognition sequence within GBP1. Evolutionary analysis of >3,300 eukaryotic *GBP* genes identified the closest GBP1 homologues in 484 species (Fig. S9A and Data S2). The majority of analyzed organisms also had a well-conserved PIM1 homologue (Data S2). The GBP1 homologues were analyzed for the presence of the corresponding phospho-serine, and the amino acid sequence upstream of the putative phospho-serine residue was compared to previously reported PIM1 recognition motifs (24–27). From this, it appeared that a basophilic kinase substrate motif (R^151^xRxKS^156^) was required for recognition of GBP1 by PIM1 (Fig. S9A). The PIM1 motif and the putative phosphorylation site (residue equivalent to Ser156) were well-conserved across evolution and their appearance in GBP sequences correlated with each other (Fig. S9A). We used a series of GBP1 mutants (Fig. S1) to assess the functional role of this motif in guiding Ser156 phosphorylation. We observed a complete loss of Ser156 phosphorylation in K155A or R153A mutants and reduced phosphorylation for R151A (Fig. S9B). Because Lys155 and Arg153 lie within the phospho-antibody epitope, their mutation could impair antibody binding. We thus used phosphoprotein-specific staining to assess phosphorylation independently. GBP1^K155A^ had residual phospho-staining levels similar to that of GBP1^S156A^, whereas GBP1^R153A^ or GBP1^R151A^ showed only a ~40% reduction (Fig. S9B). Similarly, GBP1^R151A/R153A^ and all mutants including K155A displayed reduced phosphorylation, similar to the residual level of S156A (Fig. S9B). Thus, the PIM1 phosphorylation motif in GBP1 is a basophilic kinase substrate motif.

What about phosphorylation and control of other GBPs? Having established the GBP1 recognition motif for PIM1, we next assessed the other human GBP family members (Fig. S9C). From this, it appeared that only GBP3, which is expressed at low levels in human macrophages (11), possesses a functional motif. All other GBPs have disruptive amino acid replacements in the recognition motif (GBP2, 4, 6 and 7) or a mutated phosphorylation acceptor site (GBP5, Fig. S9C). We next tested the importance of PIM1 on cellular survival. In Δ*PIM1* cells, IFNγ-stimulation alone was sufficient to induce gradual cell loss over 7 days (Fig. S9D), resembling ectopic expression of WT GBP1 in naïve cells (Fig. 1A). Notably, silencing of GBP1, but no other GBP, prevented viability loss, indicating that in the absence of PIM1, GBP1 was the only GBP activated in an uncontrolled manner (Fig. S9D). IFNγ-inducible PIM1 is thus directly responsible for phosphorylation of GBP1 on Ser156, which prevents damage to cells.

## 14-3-3σ bound phospho-GBP1 is inactive

**Fig. 3:**
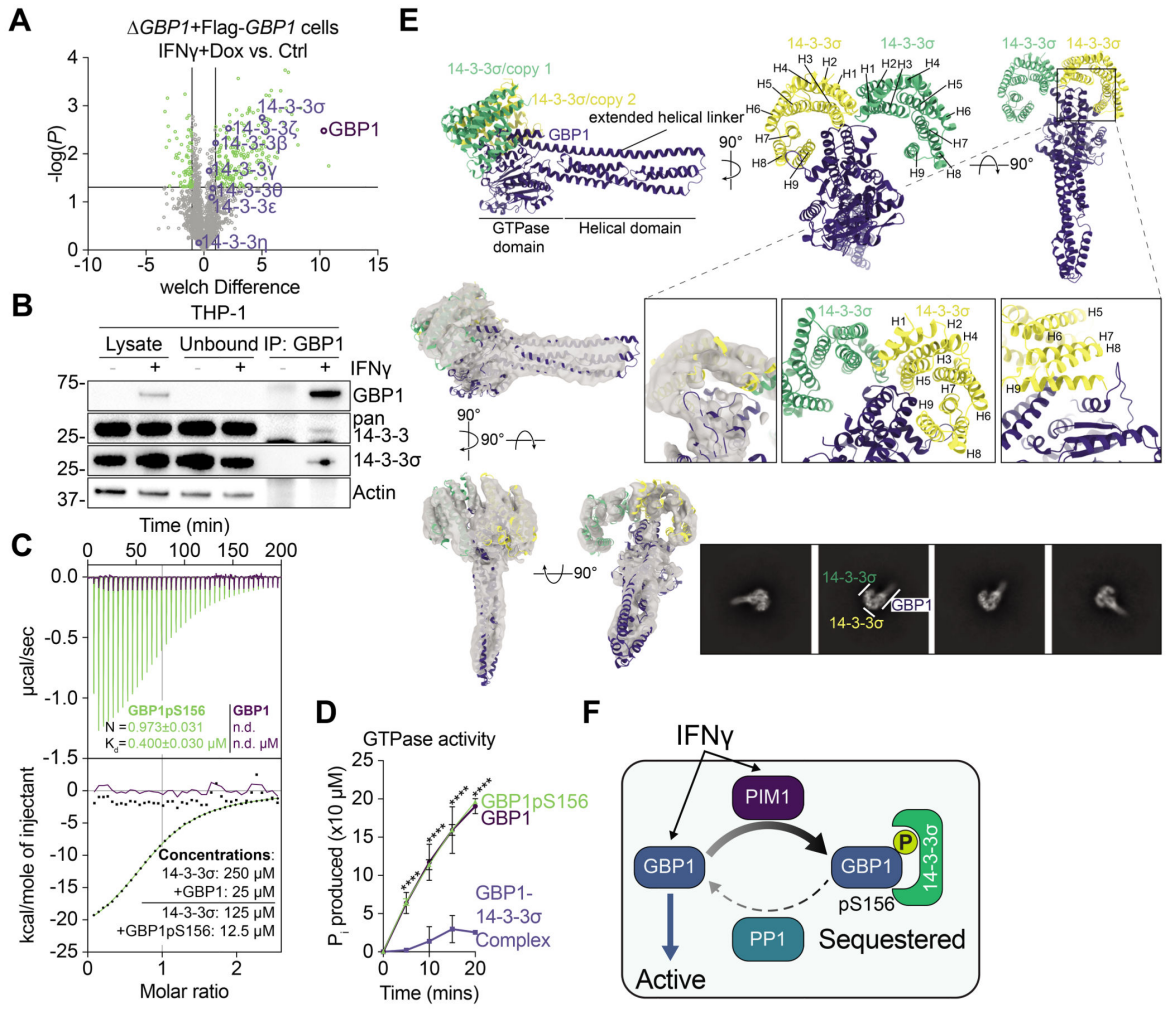
Phosphorylated GBP1 is bound and inactivated by 14-3-3σ. **(A)** Volcano plot of mass spectrometry data analysis of GBP1-interacting proteins obtained following co-immunoprecipitation of Flag-GBP1 from IFNγ (CTRL) or IFNγ+ Dox-treated THP-1Δ*GBP1*+Flag-*GBP1* cells. GBP1-interacting proteins above the significance threshold highlighted in green and 14-3-3 proteins in blue. **(B)** Immunoblots of co-immunoprecipitation of endogenous GBP1 from THP-1 WT cells treated with IFNγ. **(C)** Isothermal titration calorimetry determining thermodynamics of GBP1:14-3-3σ complex formation. 14-3-3σ at indicated concentrations was injected in 7 μL aliquots to non-phosphorylated or in vitro phosphorylated GBP1 at indicated concentrations. Determined molar ratio N at equilibrium and dissociation constant K_d_ as shown in the figure. **(D)** GTPase activity assay of 2 μM free GBP1, in vitro phosphorylated GBP1 or GBP1:14-3-3σ complex. **(E)** Top: Overview of the trimeric GBP1:14-3-3σ complex consisting of two 14-3-3 copies (yellow + green) bound to the GBP1 GTPase domain. Middle: insets depict details of GBP1:14-3-3 dimer binding interface. Cryo-EM density is shown in grey with rigid-body docked GBP1 (PDB: 1F5N) and 14-3-3σ (PDB: 1YWT) crystal structures. Bottom: Representative 2D classes with highlighted complex components. **(F)** Cartoon depicting the observed inhibitory mechanism, in which PIM1 phosphorylates human GBP1 at Ser156 which is subsequently bound by 14-3-3σ. Upon dephosphorylation, GBP1 is liberated and becomes active. **Data information:** Images in **(B)** representative of n = 3 experiments. Graph in **(A+D)** show mean ± SD from n = 3 experiments. Curves in **(C)** representative of n = 3 experiments and fitted to a model with one set of binding sites. **** *P* < 0.0001 for indicated comparisons in (D) from 2-way ANOVA following adjustment for multiple comparisons. For gel source data, see Data S5.

Ser156 of GBP1 is part of a flexible, surface-exposed loop and is the central serine of a putative, evolutionarily conserved mode I 14-3-3 binding motif (Fig. S9A and Fig. S10A). Mass spectrometry analysis of GBP1 interacting proteins in IFNγ-treated human macrophages showed the presence of all 14-3-3 proteins, including significant interactions with 14-3-3β, ζ and σ (Fig. 3A and Data S3), as expected (28). Immunoprecipitation of endogenous GBP1 confirmed its interaction with 14-3-3 proteins (pan-14-3-3 antibody) and with 14-3-3σ specifically (Fig. 3B). Immunoprecipitation of co-transfected Flag-GBP1 and HA-tagged 14-3-3 proteins (β, γ, ε, ζ, η, θ, σ) showed promiscuous interaction of all 14-3-3 proteins with GBP1 WT but not with GBP1^S156A^ or GBP1^R153A/P158A^ carrying a mutated 14-3-3 binding motif (Fig. S10B). The GBP1^T590A^ mutation did not affect its binding to 14-3-3 proteins (Fig. S10B). We thus further examined the potential roles of Ser156 phosphorylation. The mRNA levels for the seven 14-3-3 proteins varied in human macrophages, with the abundance of 14-3-3σ being the lowest in naïve cells and showing an increase upon IFNγ-treatment (Fig. S10C and Fig. S6). Expression of the other 14-3-3 family members remained unchanged (Fig. S10C). Semi-quantitative co-immunoprecipitations of phosphorylated, endogenous GBP1 and purified HA-tagged 14-3-3 proteins corroborated our findings and showed the highest binding affinity between GBP1 and 14-3-3σ (K_d_ = 0.37±0.10 μM (SD); Fig. S11). This was independently confirmed by isothermal titration calorimetry (ITC) with recombinant 14-3-3σ and in vitro phosphorylated recombinant GBP1 (Fig. 3C and Fig. S12). Unphosphorylated GBP1 did not bind 14-3-3σ (Fig. 3C), confirming its specific interaction with the phosphorylated protein.

We next reconstituted the GBP1-PIM1-14-3-3σ interaction in vitro and found that they formed a ~125 kDa complex in the presence of ATP, most likely composed of one monomer of GBP1 (67 kDa) and a 14-3-3σ dimer (dimer: 56 kDa) (Fig. S13A). The stable phospho-GBP1:14-3-3σ complex could be purified and analyzed by size exclusion chromatography (Fig. S13B). Dimerization of GBP1 occurred in absence of GTP but increased markedly in the presence of GTP or GDP+AlF_3_. The latter locks GBP1 in a GDP-bound state, showing that conformational changes in the GBP1 GTPase domain are required for its dimerization (Fig. S13C). Phosphorylated and 14-3-3σ-bound GBP1 did not dimerize (Fig. S13C). Furthermore, the rate of GTP hydrolysis by GBP1 in a GBP1:14-3-3σ complex was reduced by >8-fold as compared to free GBP1 (Fig. 3D). Phosphorylation of GBP1 without 14-3-3σ-binding did not influence the rate of GTP hydrolysis (Fig. 3D).

To visualize the interaction between phosphorylated GBP1 and 14-3-3σ, we solved the structure of the purified protein complex (Fig. S13B) using single particle cryogenic electron microscopy (cryo-EM, Fig. 3E, for details see Fig. S14 and Table S1). We obtained a cryo-EM map of the GBP1:14-3-3 heterotrimer at 5 Å resolution, confirming binding of one 14-3-3σ homodimer to a single copy of GBP1 by grabbing its GTPase domain and interacting with the linker helix that connects the helical domain to the GTPase domain (Fig. 3E). While the overall resolution did not permit de novo modelling, we were able to unambiguously dock previously reported crystal structures as rigid bodies based on secondary structure features (Fig. 3E). Close interactions were observed for GBP1 loop R183-T197 with the termini of helix 8+9 of 14-3-3σ copy 1. Furthermore, 14-3-3σ helices 5+9 were in contact with the parallel-running linker helix of GBP1 (Fig. 3E). In this position the phosphate binding site of 14-3-3σ copy 2 was in close proximity to GBP1 Thr590, for which we also observed phosphorylation (Fig. 2F) but no effect on 14-3-3-binding upon mutation of GBP1 Thr590 (Fig. S10B). Thus we hypothesize that 14-3-3σ binding to GBP1 functions in a similar way as 14-3-3 sequestration of Rnd3 GTPase (29), requiring two phosphorylation sites and the farnesyl moiety. For GBP1, both Ser156 and Thr590 would be involved in sequential two-step 14-3-3-binding, with the initial recognition by Ser156 and subsequent stabilization by binding to Thr590. In summary, our model suggests that phosphorylated GBP1 is sequestered by 14-3-3σ, acting as a molecular padlock to keep the protein inactive through inhibition of dimerization and GTPase-activity (Fig. 3F).

## PIM1 and 14-3-3σ control GBP1 activities

**Fig. 4:**
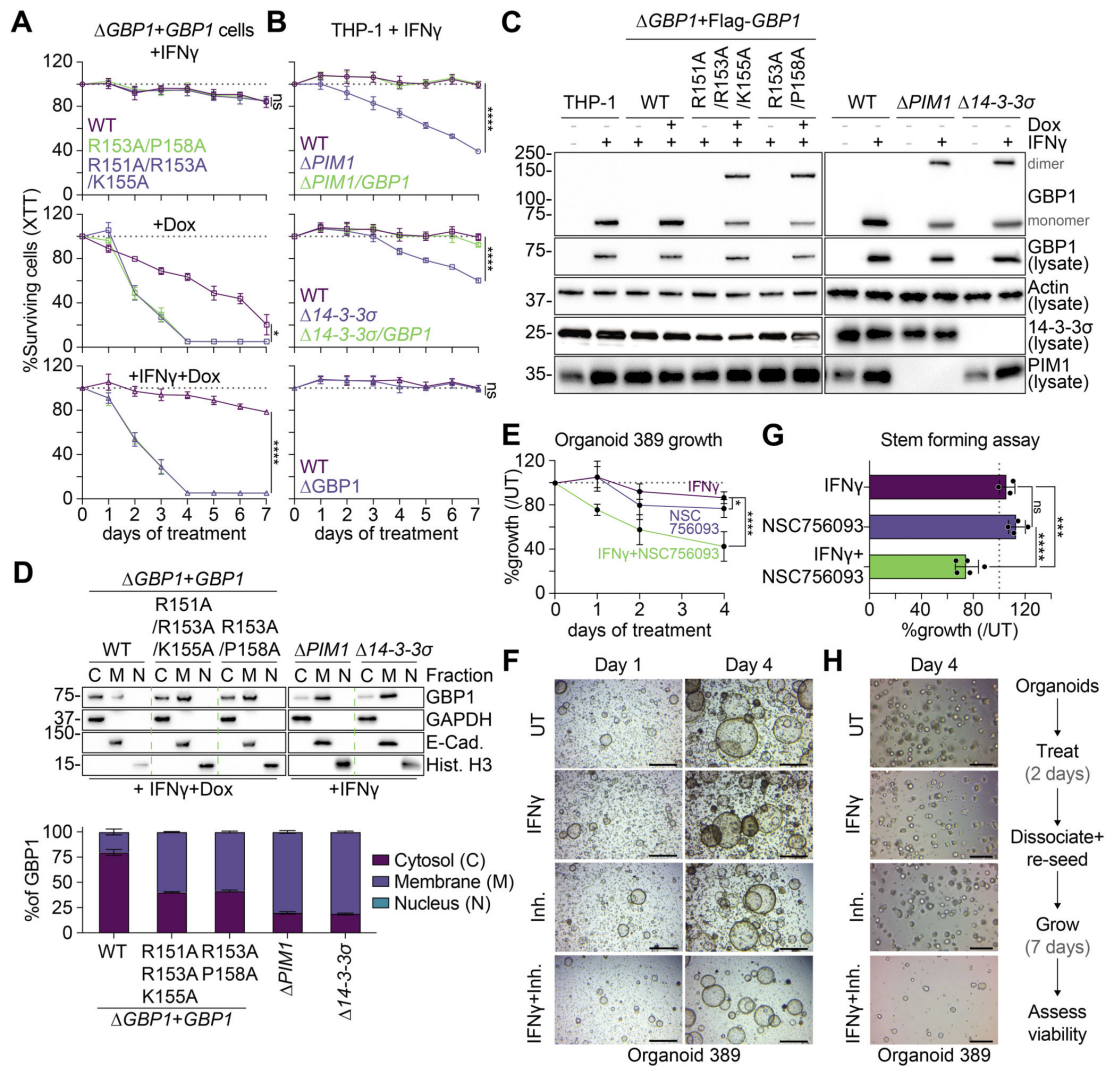
PIM1 phosphorylation of GBP1 protects cells from self-inflicted damage. **(A)** XTT cell survival assay of THP-1Δ*GBP1*+*GBP1* WT, +*GBP1*^R151A/R153A/K155A^ (kinase motif mutant) or +GBP1^R153A/P158A^ (14-3-3 binding motif mutant) cells treated with IFNγ±Dox for indicated number of days. **(B)** XTT cell survival assay kinetics of THP-1Δ*GBP1*, Δ*PIM1*, Δ*14-3-3σ*, Δ*PIM1/GBP1* or Δ*14-3-3σ*/*GBP1* cells treated with IFNγ for indicated number of days. **(C)** Immunoblot-analysis of GBP1 in crosslinked pellets and in cell lysates of indicated cells treated with IFNγ±Dox or left untreated. **(D)** Immunoblots of subcellular fractionation to determine GBP1 localization in indicated cells with respective markers for the cytosol (C), membranes (M) or the nucleus (N) and quantification of localization. **(E)** MTT survival assay of patient-derived colorectal tumour organoids treated with IFNγ and NSC756093 for 4 days, shown as percent growth compared to non-treated controls. **(F)** Images of organoids following indicated treatments with IFNγ and inhibitor NSC756093 (Inh.). Scale bar 1 mm. **(G)** Stem forming assay of organoids treated for 2 days as indicated, dissociated, and re-grown for 7 days in the absence of treatment. MTT assay on day 7 following re-seeding indicating relative re-growth. **(H)** Representative images from day 4 of re-growth of stem forming assay. Scale bar 200 μm. **Data information:** Images in **(C-D)** and **(F-H)** representative of n = 3 experiments. Graphs in **(A-B), (D-E)** and **(G)** show mean ± SEM n = 3 experiments, normalized to untreated cells. * P ≤0.05; *** P ≤ 0.001; **** P ≤ 0.0001 in **(A-B)**, **(E)** and **(G)** comparing to WT or untreated (UT) cells and for indicated comparisons in **(E)** from one-way ANOVA and in. For gel source data, see Data S5.

Having identified a role for PIM1 and 14-3-3σ in inhibiting the activity of GBP1, we next asked whether PIM1 and 14-3-3σ protected cells from the toxic effects of GBP1. We used CRISPR/Cas9 to delete *14-3-3σ* (encoded by the gene *SFN*, also called *YWHAS*, Fig. S7A) and reconstituted the cells with Dox-inducible 14-3-3σ (Fig. S7C). Next, we measured long-term survival of cells forced to express GBP1 mutants that cannot be recognized and inactivated by PIM1 (GBP1^R151A/R153A/K155A^) or 14-3-3σ (GBP1^R153A/P158A^). Similar to WT GBP1 (Fig. 1A), ectopic expression of these GBP1 mutants led to complete cell loss, pointing to their toxicity (Fig. 4A). Like the phospho-deficient GBP1^S156A^ (Fig. 1E), IFNγ treatment was unable to prevent cell death (Fig. 4A). We then measured long-term survival of WT, Δ*GBP1*, Δ*PIM1*, Δ*PIM1/GBP1*, Δ*14-3-3σ* or Δ*14-3-3σ*/*GBP1* cells. IFNγ-treatment of Δ*PIM1* cells led to cell loss, as previously observed (Fig. S9D), as did IFNγ-treatment of Δ*14-3-3σ* cells (Fig. 4B). In both cases cell loss was prevented by additional knockout of GBP1, whereas IFNγ-treatment of THP-1 WT or Δ*GBP1* cells did not reduce viability (Fig. 4B). Moreover, both GBP1 mutants and endogenous GBP1 in Δ*PIM1* or Δ*14-3-3σ* cells were prone to dimerization (Fig. 4C) and localization to cellular membranes in IFNγ-exposed macrophages (Fig. 4D). Again, this resembled the phosphorylation-deficient S156A mutant (Fig. 1F+G).

To test whether this mechanism holds true at the tissue-level, we used IFNγ-responsive, patient-derived colorectal tumor organoids and tested if the GBP1:PIM1 interaction inhibitor NSC756093 affected cell viability, organoid growth and stemness. IFNγ-treatment alone had no impact on organoid survival/growth and the inhibitor NSC756093 alone only caused mild cytotoxicity (Fig. 4E). However, treatment with a combination of IFNγ and inhibitor (and thereby unleashing GBP1 from its phosphorylation-driven control) decreased organoid growth by ~60% over the course of 4 days (Fig. 4E). Visual inspection confirmed these findings, showing fewer and smaller organoids upon treatment with IFNγ and NSC756093 compared to untreated, IFNγ- or inhibitor-only treated samples. (Fig. 4F). To further interrogate the effects of treatment with IFNγ and NSC756093, we next evaluated the stemness of organoids using stem forming assays. To do so, organoids pre-treated with IFNγ and NSC756093 for 48 hours were dissociated, re-seeded, and left to grow back into organoids for 7 days without any additional treatment (Fig. 4G-H). This revealed a lasting effect of IFNγ and NSC756093 on the ability of organoid precursors to self-renew and illustrates the potency of GBP1 in colorectal tumor organoids (Fig. 4G+H). In summary, PIM1 and 14-3-3σ are required to control the activity of GBP1 in human cells via phosphorylation and cytosolic sequestration.

## *Toxoplasma* infection depletes PIM1 and activates GBP1

**Fig. 5:**
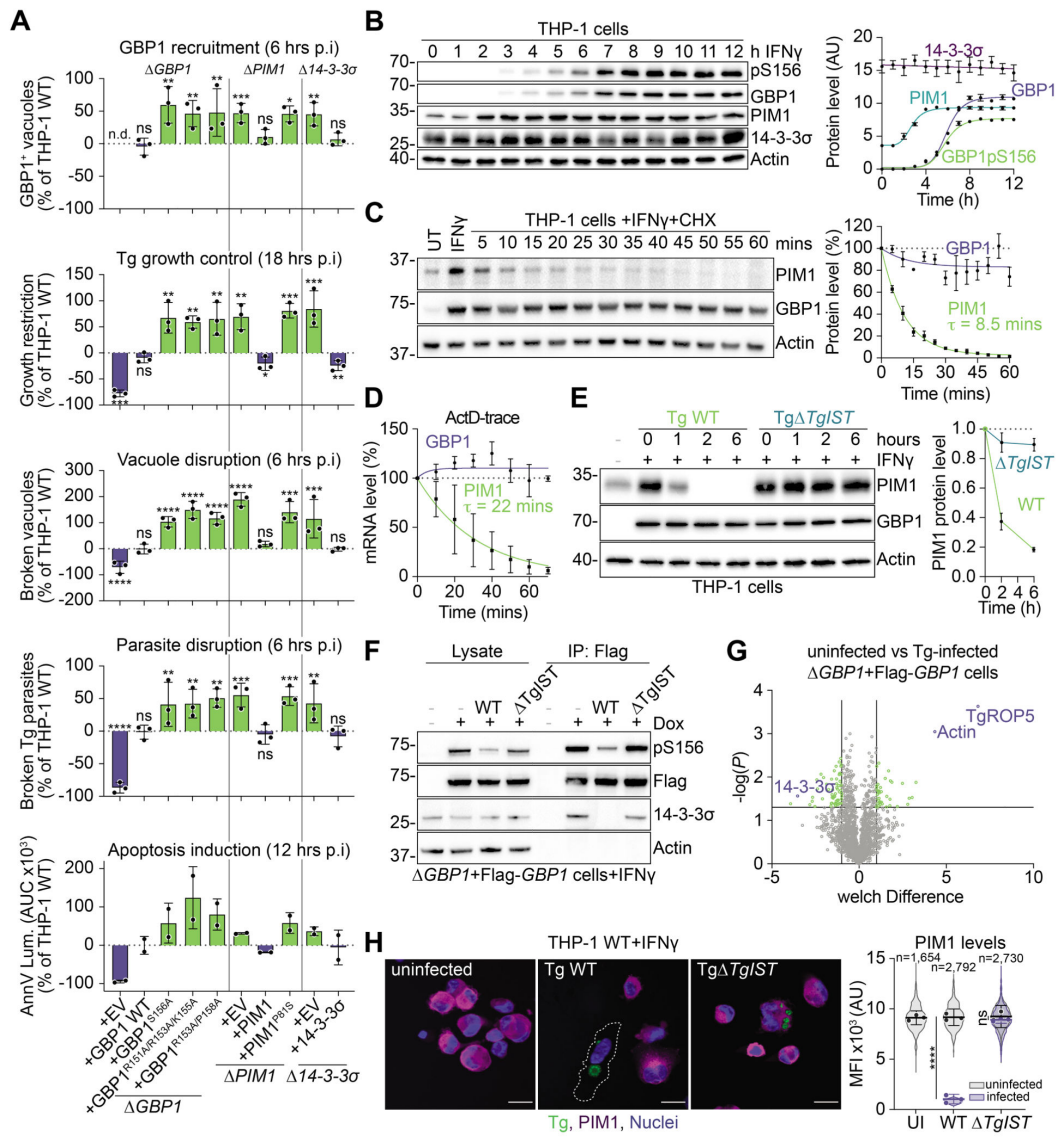
Toxoplasma infection depletes PIM1 and activates GBP1. **(A)** Quantification of GBP1 recruitment to *Toxoplasma gondii* (Tg) vacuoles, Tg growth restriction, vacuole/parasite disruption and apoptosis assay (area under the curve, AUC) in IFNγ+Dox-treated cells expressing the indicated mutant of GBP1, Δ*PIM1* or Δ*14-3-3σ* cells reconstituted with the indicated protein or empty vector (EV). Values plotted relative to IFNγ-primed WT cells. **(B)** Immunoblots of IFNγ-treated THP-1 WT cells and protein level quantification. **(C)** Immunoblots of THP-1 WT cells treated with 50 μg/mL cycloheximide (CHX) and quantification of protein half-life. **(D)** RT-qPCR determination of mRNA stabilities in THP-1 WT cells treated with Actinomycin D (ActD). **(E)** Immunoblots and quantification of PIM1 protein level in THP-1 WT cells treated with IFNγ and infected with Tg WT or Δ*TgIST*. **(F)** Immunoblots of Flag-GBP1 co-immunoprecipitation from IFNγ/Dox-treated THP-1Δ*GBP1*+Flag-*GBP1* cells infected with Tg WT or Δ*TgIST* for 6 hours. **(G)** Mass spectrometry analysis of GBP1-interacting proteins in uninfected (UI) or Tg WT-infected and IFNγ+Dox-treated THP-1Δ*GBP1*+Flag-*GBP1* cells. **(H)** Immunofluorescence images of IFNγ-primed and uninfected (UI) or Tg WT or Δ*TgIST*-infected THP-1 WT after 12 hours. White: cell outline; Magenta: PIM1; Green: Tg; Blue: Nuclei. Scale bar: 20 μm. Graph shows quantification of PIM1 MFI depending on infection status: Grey, uninfected cells; Blue, infected cells. **Data information:** Images in **(B, C, F, E, H)** representative of n = 3 experiments. Graphs in **(A)** show mean ± SD of n = 3 experiments. Graphs in **(B+C)** show mean ± SD from n = 3 and in **(D+E)** from n = 4 experiments. Graphs in **(G)** shows data from n = 3 replicates. * *P* ≤*0.05*; ** *P* ≤ *0.01*; *** *P* ≤ *0.001*; **** *P* ≤ *0.0001* in **(A)** from 2-way ANOVA comparing to THP-1 WT cells and in **(H)** from nested t-test comparing infected to uninfected cells following adjustment for multiple comparisons; ns, not significant. For gel source data, see Data S5.

We hypothesized that in the absence of phosphorylation of GBP1 by PIM1 and its sequestration by 14-3-3σ, GBP1 is free to target pathogen compartments more effectively. To test how control of GBP1 affects its trafficking and antimicrobial activities, we quantified its ability to restrict growth of *Toxoplasma gondii* (Tg). We tested cells that expressed GBP1 mutants with impaired phosphorylation (S156A, R151A/R153A/K155A) or binding of 14-3-3σ (R153A/P158A), cells that lacked PIM1 (or expressing kinase-dead PIM1^P81S^) or that lacked 14-3-3σ. All displayed increased recruitment of GBP1 to Tg vacuoles, restricted growth of Tg more efficiently and showed increased disruption of Tg-containing vacuoles and parasites, with subsequent higher induction of apoptosis as compared to IFNγ-treated THP-1 WT controls (Fig. 5A). This indicated increased parasiticidal activity of GBP1 in conditions that prevented GBP1 phosphorylation and/or its interaction with 14-3-3σ. Reconstituting knockout cells with WT PIM1 or 14-3-3σ respectively, decreased all pathogen control measures back to THP-1 WT levels (Fig. 5A).

When assessing dynamics of the association of GBP1 with Tg-containing vacuoles, GBP1 was recruited faster and to higher levels in Δ*PIM1* or NSC756093-treated THP-1 WT cells (Fig. S15A-B). Fluorescence recovery after photobleaching assays (FRAP) showed increased mobility of mCH-tagged GBP1 in Δ*PIM1* cells or cells that express mCH-GBP1^S156A^, which could explain the faster kinetics of GBP1 recruitment to Tg vacuoles (Fig. S15C). Conversely, silencing of *PPP1CA* impaired growth control of Tg, whereas treatment with NSC756093 improved it. Depletion of PIM2/3 affected neither GBP1 recruitment to Tg vacuoles nor growth control of Tg (Fig. S15D-F).

How did GBP1 phosphorylation and protein abundance change in Tg-infected cells? We first measured the induction of GBP1, 14-3-3σ and PIM1 as well as the appearance of Ser156-phosphorylated GBP1 in THP-1 WT cells following IFNγ-treatment (Fig. 5B). This time course showed more rapid induction of PIM1 than GBP1. Contrary to our RT-qPCR data (Fig. S10C), the levels of 14-3-3σ did not change (Fig. 5B). Notably, GBP1 appeared to be phosphorylated early upon its expression (Fig. 5B), indicating that the PIM1/14-3-3σ-mediated control of GBP1 was immediately active. PIM1 protein and mRNA were unstable and short-lived (τ_1/2_ ~8.5 minutes or ~22 minutes respectively) as compared to more stable GBP1 (protein τ_1/2_ >12 hours; Fig. 5C-D). This led us to hypothesize that PIM1 degradation might serve to uncouple the inhibition of GBP1. In this way, IFNγ-induced PIM1 serves a dual role: as a guard that detects pathogen-mediated interference of IFNγ-signaling (leading to depletion of PIM1), and to protect self-membranes from damage inflicted by GBP1. Tg secretes a pathogen effector protein known as *T*. *gondii* inhibitor of STAT1 transcriptional activity (TgIST). Upon entering the host cell nucleus, TgIST sequesters STAT1 on specific loci and facilitates formation of nonpermissive chromatin by recruiting the nucleosome remodeling deacetylase (NuRD) repressor complex. The nascent transcription sites thereby remain silenced (30, 31). Given the IFNγ-inducible nature of the *PIM1* gene, we hypothesized that TgIST completely shuts down *PIM1* gene expression, leading to loss of PIM1. Indeed, we observed rapid TgIST-dependent depletion of PIM1 upon infection with Tg (Fig. 5E), further precipitating the loss of GBP1 phosphorylation and interaction with 14-3-3σ (Fig. 5F). Mass spectrometry of GBP1 interactomes of Tg-infected cells, confirmed decreased interaction of GBP1 with 14-3-3σ and showed increased interaction with host-derived actin and parasite-derived TgROP5 compared to uninfected cells (Fig. 5G and Data S4). These experiments showed that the activity of GBP1 is restrained in uninfected cells but can be rapidly triggered upon infection by a pathogen that interferes with IFNγ-signaling. Supporting our hypothesis of bystander cell protection, we found that PIM1 was depleted specifically only in Tg WT-infected macrophages, but not in uninfected cells from that same experiment or in Δ*TgIST*-infected cells (Fig. 5H).

Finally, we wanted to investigate the effect of PIM1 and 14-3-3σ on GBP1 activity against Gram-negative bacteria such as *Salmonella* Typhimurium (STm) which is distinct from its activity against vacuolar pathogens such as Tg. For comparison we assessed the cellular distribution of PIM1, 14-3-3σ, GBP1 and Ser156-phosphorylated GBP1 in Tg and STm-infected cells by immunofluorescence imaging (Figure S16). In uninfected cells, all proteins showed a high degree of colocalization (Pearson’s coefficient >0.88, Fig. S16A). In Tg-infected cells we observed rapid recruitment of GBP1 to Tg vacuoles but no recruitment of PIM1, 14-3-3σ or GBP1pS156 (Fig. S16B). This corroborated our finding that GBP1 must be dephosphorylated and released from sequestration, prior to acquiring its parasiticidal, pathogen-proximal activity. We also observed decoration of the bacterial surface of cytosolic STm by GBP1 alongside recruitment of PIM1, 14-3-3σ and GBP1pS156 (Fig. S16C), suggesting that GBP1’s high-affinity to LPS might override control by PIM1-mediated phosphorylation and 14-3-3σ sequestration. Supporting this finding, we did not observe degradation of PIM1 or dephosphorylation of GBP1 upon STm-infection (Fig. S16D). Finally, in Δ*14-3-3σ* cells, phosphorylated GBP1 was recruited to Tg vacuoles, demonstrating that retention by 14-3-3σ in the cytosol, not phosphorylation by PIM1 alone, ultimately controlled GBP1 pathogen-compartment targeting (Fig. S16E).

## Discussion

Human GBP1 is a membrane-active large GTPase that can disrupt microbe-encapsulating membranes (5–15). However, GBP1 can also associate with the Golgi apparatus (16, 20) and/or the plasma membrane (13, 16). How GBP1 activity is regulated in cells and how it is recruited to specific subcellular sites has remained elusive. Here we found that a large fraction of GBP1 is phosphorylated by the kinase PIM1 at Ser156, establishing that post-translational modification of human GBP1 regulates its activity. Ser156-phosphorylated GBP1 was sequestered in the cytosol by 14-3-3σ, thereby preventing self-inflicted damage, in a mechanism reminiscent of how Rnd3 GTPase is inactivated (29). Thus, IFNγ-exposed macrophages are in an activated state but keep GBP1 on stand-by and its activity tightly controlled. In uninfected cells, ectopic expression, and unrestrained action of GBP1 led to necrosis. Similarly, ectopic expression of GBP1 in ovarian cancer cells reduces their viability by ~25% within 3 days (32). In infected cells, the Tg-induced block of IFNγ signaling-induced transcription depletes inherently short-lived PIM1 (33) and springs a trap that unleashes GBP1 onto the pathogen-containing compartment. Pathogens from all kingdoms of life have evolved effector proteins that interfere with IFNγ-signaling integrity. Some examples include *Mycobacterium tuberculosis* blocking transcriptional transactivation downstream of STAT1 (34), *Vaccinia virus* phosphatase H1 targeting STAT1 for dephosphorylation (35), Human cytomegalovirus depleting JAK kinases (36) and Hepatitis C virus NS5A protein or *Leishmania donovani* both disrupting STAT1 phosphorylation (37, 38). The cytokine-induced kinase PIM1 may thus serve as a detector for a broad range of pathogens that interfere with IFNγ signaling. Consistent with our work, PIM1 is a host factor that favors survival of Tg (39, 40). Furthermore, PIM1 is a known GBP1-interacting protein in cancer cells (21) and is a pro-survival, proto-oncogene that negatively regulates other pro-apoptotic proteins (24, 26, 41–44). Given GBP1’s ability to promote programmed cell death, use of the specific GBP1:PIM1 interaction inhibitor (21) is a promising way to chemically activate GBP1, with implications for innate immunity and cancer therapy.Innate immune mechanisms that can cause cellular damage must be carefully regulated to ensure protection of bystander cells. The activity of immune effectors is tightly controlled, either indirectly via inactivation of inhibitory guard proteins, or directly via activation of upstream PRRs. The indirect guard mechanisms, originally described in plants (45), detect interference of pathogens with key cellular processes, whereas the direct PRR mechanisms respond to conserved pathogen-associated molecular patterns. An example of such ‘guarding’ in Mammalia, conceptually similar to inactivation of GBP1, is regulation of the Pyrin inflammasome, where the cytoskeleton-sensing kinases PKN1/2 phosphorylate Pyrin and inactivate the protein via binding of 14-3-3 proteins. This system relays microbial perturbation of the cytoskeleton and thereby indirectly triggers immunity (46–49). Other recent examples include ‘self-guarding’ of MORC3 which controls HSV-1 infection and secondary type I IFN responses (50) or viral disruption of protein synthesis that triggers apoptosis via depletion of short-lived BCL-2 protein family members (51). Given the short half-life of PIM1, it is tempting to speculate that translation inhibition might also unleash GBP1 from its restriction. GBP1 directly activates immunity through its bona fide PRR function for cytosolic LPS (7, 11, 13–15). As we found here, its other, parasiticidal function is controlled by PIM1. Thus, GBP1 combines both innate immune surveillance strategies and antimicrobial activity in a single effector protein. The integration of an IFNγ-induced pathway with post-translational control of innate immune defense could cover a broad range of pathogens and enable cells to autonomously regulate immunity depending on their infection status. Similar cytokine-induced guard circuitries are likely to exist and thus ensure protection of innocent bystander cells.

## Materials and Methods

### Cell, parasite and bacteria culture, infections, and treatments

THP-1 cells (TIB202, ATCC) were maintained in RPMI with GlutaMAX (35050061, Gibco) supplemented with 10% FBS (Sigma), at 37°C in 5% CO_2_. THP-1s were differentiated with 50 ng/mL phorbol 12-myristate 13-acetate (PMA, P1585, Sigma) for 3 days and left to rest for 2 days by replacing the differentiation medium with complete medium without PMA. HEK293T (Cell Services, The Francis Crick Institute, London, UK), NIH3T3 (CRL-1658, ATCC) and human foreskin fibroblasts, HFFs (SCRC-1041, ATCC), were cultured in DMEM with GlutaMAX (Gibco) supplemented with 10% FBS (Sigma), at 37°C in 5% CO_2_. Cells were not used beyond passage 25 and HFFs not beyond passage 15.

Patient-derived colorectal tumor organoids (Organoid 389), originally derived from a T3N1 poorly differentiated, mismatch repair proficient adenocarcinoma of the sigmoid colon from a male subject (52), were grown and maintained in 50 μL Matrigel domes (CLS356231, Corning) with 500 μL human Intesticult medium (06010, StemCell) in 24-well plates at 37°C and 5% CO_2_ and passaged weekly.

Tg were maintained by serial passage on HFF cells, cultured in DMEM with GlutaMAX supplemented with 10% FBS, at 37°C in 5% CO_2_. Parasites were passaged the day before infection to maintain high viability. Tg were prepared from freshly 25G syringe lysed HFF cultures by centrifugation at 50 × g for 3 minutes, transferring the cleared supernatant into a new tube, subsequent centrifugation at 500 × g for 7 minutes and re-suspension of the pelleted parasites into fresh complete medium. Parasites were added to the cells at a MOI of 1. Infection was synchronized by centrifuged at 500 × g for 5 minutes. Two hours post-infection, extracellular parasites were removed with three washes using warm PBS (806552, Sigma) and fresh complete medium was added prior to culturing at 37°C, 5% CO_2_ for the required time.

STm strain SL1344-GFP (11) was maintained under Ampicillin selection (11593027, Gibco), and grown on LB + Ampicillin agar plates. One day prior to infection bacteria from a single colony were inoculated and grown overnight at 37°C. The overnight culture was diluted 1:20 into LB + 300 mM NaCl and grown shaking in a closed container to an OD_600_ of 0.9. Bacteria were harvested by centrifugation at 1,000 × g for 5 min, washed with serum-free cell culture medium twice and re-suspended in 1 ml plain medium. Cells were infected with STm at an MOI of 10, and infections were synchronized by centrifugation at 750 × g for 10 min. Infected cells were washed three times with warm PBS 30 min post-infection, and fresh medium containing 10 μg/ml gentamicin (15750060, Gibco) was added. Bacterial MOI used for infections was confirmed by plating on LB agar plates. An overview of all cell lines, parasites and bacteria strains can be found in Table S2. All cell culture work was performed without addition of antibiotics and the cells were regularly tested for mycoplasma contamination by immunofluorescence and PCR.

THP-1 cells were stimulated for 16 hours prior to infection in complete medium with addition of 50 IU/mL human IFNγ (285-IF, R&D Systems). Induction of protein expression in the Dox-inducible cells was performed with 200 ng/mL Dox (D9891, Sigma) overnight. To block translation for studying protein stability, cells were treated with 50 μg/mL cycloheximide (CHX, C7698, Sigma), and to block transcription with 6 ng/mL actinomycin D (A1410, Sigma). To block GBP1:PIM1 interaction, cells were treated with 100 nM NSC756093 (SML1310, Sigma). For in vitro assays, NSC756093 was used at 1 μM. To chemically induce pyroptosis, cells were primed with 100 ng/mL LPS O111:B4 (NC9673121, Enzo) and subsequently treated with 10 μM nigericin. Treatments to induce apoptosis were performed with 50 ng/mL TNFα (210-TA, R&D Systems) and 10 μg/mL CHX. The pan-caspase-inhibitors zVAD-fmk (25 μM, 60332, Cell Signalling Technologies) or qVD (20 μM, ab141421, Abcam) were used to block caspase activation for necroptosis induction or blocking apoptosis/pyroptosis. To inhibit pyroptosis cells were treated with 10 μM Mcc950 (inh-mcc, Invivogen) or 50 μM disulfiram (DSF, 1224008, Sigma). To inhibit necroptosis cells were treated with 50 μM GSK’872 (6492, Tocris) or 10 μM necrosulfonamide (NSA, 5025, Tocris). Tumor organoid cultures were treated with 100 IU/mL IFNγ or 1.66 μM NSC756093.

### Plasmid DNA and siRNA transfection

THP-1 cells were transfected with 30 nM siRNAs two days prior to infection (Table S3). The transfection mix was prepared as a 10X mix in OptiMEM containing the siRNA(s) and TransIT-X2 transfection reagent (MIR600x, Mirus) in a 1:2 ratio. All siRNAs were ON-TARGETplus pools from Dharmacon with the negative control ON-TARGETplus Non-targeting Pool (D-001810, Dharmacon). Transfection of DNA plasmids was performed using Lipofectamine 2000, following the manufacturer’s instructions (11668027, Invitrogen).

### Lentiviral transduction

Lentiviral packaging used HEK293T cells transfected with equimolar ratios of the required expression plasmid, pMD2.G (Addgene #12259) and psPAX2 (Addgene #12260, both gifts from Didier Trono) using Lipofectamine 2000 in serum-free DMEM. Medium was replaced 12 hours post-transfection with fresh DMEM + 10% FBS to rest the cells and again after 12 hours with DMEM + 10% FBS containing 5 mM sodium butyrate (B5887, Sigma). The next day, medium was replaced with RPMI + 10% FBS and the cells left to produce lentiviral particles for one day. Virus containing supernatant was filtered through a 0.43 μm syringe filter and supplemented with 8 μM polybrene (H9268, Sigma). The target cells were then resuspended in 500 μL of the virus containing medium and ‘spinfected’ for 30 minutes at 1,000 × g. One hour p.i. 1 mL complete medium was added, and the cells were left to rest. The infection procedure was repeated for a total of three times before selection with the appropriate selection reagent: 1 μg/mL Puromycin (A1113802, Gibco), 200 μg/mL Zeocin (J67140, Alfa Aesar) and/or 15 μg/mL Blasticidin S (15205, Sigma). Once untransduced control cells had died in the selection medium, the newly created cells were verified for successful transduction by immunoblotting.

### Creation of new cell lines

#### Inducible GBP1 cell lines

THP-1Δ*GBP1*, THP-1Δ*GBP1*+Tet-*GBP1* WT, THP-1Δ*GBP1*+Tet-Flag-*GBP1* WT and THP-1Δ*GBP1*+Tet-mCH-*GBP1* WT have been published before and new cell lines were created identically using Lentiviral transductions (11). To make cells expressing mutated GBP1, pLenti-Tet-GBP1 plasmids (11) with and without tags, were mutated using site-directed mutagenesis and transduced into the THP-1Δ*GBP1*+Tet target cells using lentiviral transduction as described before.

#### PIM1 and 14-3-3σ (SFN/YWHAS) CRISPR knockout cells

Guide RNA (gRNA) sequences targeting the 5’ and 3’ UTR of the respective gene were designed using crispr.mit.edu (Table S4). DNA oligonucleotides encoding the crRNA were annealed and cloned into BsmBI-digested (ER0451, Thermo Scientific) pLentiCRISPR-V2 backbone (53) using Quick Ligation™ kit (M2200, NEB) and transduced into THP-1 WT or THP-1Δ*GBP1* cells using Lentiviral particles. Following selection with puromycin for 7 days, cells were sub-cloned by serial dilution into 96-well plates using pre-conditioned complete medium supplemented with non-essential amino acids (11140076, Gibco), penicillin/streptomycin and GlutaMAX. Roughly 3 weeks after seeding, obtained clones were expanded and screened for absence of target protein expression by RT-qPCR. Clones that showed reduced or absent target protein expression underwent secondary screening by immunoblotting. For each of the cell lines, >4 single-cell clones with confirmed absence of PIM1 or 14-3-3σ protein respectively were pooled and cultured for two more weeks before undergoing final screening to confirm absence of the proteins. This resulted in the THP-1Δ*PIM1*, Δ*PIM1/GBP1*, Δ*14-3-3σ* and Δ*14-3-3σ*/*GBP1* cell lines.

#### Reconstitution of PIM1 and 14-3-3σ

CRISPR knockout cells of PIM1 and 14-3-3σ were transduced with the Dox-inducible system as previously described (11). To create PIM1 and 14-3-3σ expressing Dox-inducible plasmids (pLenti-Tet-*PIM1* or pLenti-Tet-*14-3-3σ*), the empty vector backbone was digested with BamHI, PIM1 and 14-3-3σ ORFs amplified from cDNA obtained from IFNγ-primed THP-1 WT cells and all fragments assembled with Gibson assembly. Further, the pLenti-Tet-PIM1 plasmid was mutated by site-directed mutagenesis to obtain pLenti-Tet-PIM1^P81S^ kinase-dead version and all vectors transduced into the respective target cells using lentiviral transduction.

### Quantitative RT-PCR

RNA was extracted from 0.25×10^6^ cells using Trizol reagent (15596026, Invitrogen). GlycoBlue (5 μg/mL, AM9516, Invitrogen) was added during the isopropanol precipitation step. RNA quality was measured on a Nanodrop 2000 Spectrophotometer (Thermo Scientific). 1 μg RNA was reverse transcribed using high-capacity cDNA synthesis kit (4368813, Applied Biosystems). qPCR used the PowerUP SYBR green kit (A25742, Applied Biosystems), 20 ng cDNA in a 10 μL reaction and primers at 1 μM final concentration (Sequences in Table S5). Primer specificity was ensured by designing primers to span exon-exon junctions, whenever possible, and for each primer pair a melt curve was recorded, compared to in silico predicted melt curves from uMelt (54) and amplicon sizes analyzed by agarose gel electrophoresis. Recorded Ct values were normalized to the recorded Ct of human *HPRT1*, and data plotted as ΔCt (Relative expression). To determine absolute expression, defined amounts of linearized plasmid standards were used as PCR template and obtained Ct values used to calculate transcript numbers.

### MTT/XTT and AnnV glow assay for cell survival and death

To determine cell viability using XTT assays (Cell proliferation kit II, Roche), 25,000 cells were seeded per well in a 96-well plate or 10,000 cells in a 384-well plate and differentiated as described before. Cells were treated in RPMI without phenol red (11835, Gibco). The detection reagent was freshly prepared according to the manufacturer’s instructions and added prior to the viability determination. Cells were then incubated at 37°C for 4 hours and absorption was measured at 475 nm and 660 nm for correction.

For organoid growth analysis, MTT reagent (M6494, Invitrogen) was added in a 1:10 dilution to the plates and the Matrigel domes disrupted by pipetting. Plates were returned to a 37°C incubator for 30 minutes, before adding lysis solution (4 mM HCl, 0.1% Triton-X100 in isopropanol), mixing by pipetting, followed by 10 minutes on a shaker in the dark. Each well was thoroughly mixed, and lysates transferred in quadruplicates to a microplate and absorbance read at 550 nm on an iMark Microplate Absorbance Reader (Biorad). Medium with Matrigel was used as a negative control.

Apoptosis kinetics were analyzed using the RealTime-Glo™ Annexin V Apoptosis Assay (JA1001, Promega) according to the manufacturer’s instructions. In brief, 50,000 cells were seeded per well of a white, tissue culture-treated 96-well plate, differentiated, pre-treated, and infected. Simultaneously with infection, detection reagent was added. Luminescence was measured using a Fluostar Omega plate reader (BMG Labtech) pre-heated to 37°C. No-cell, medium-only controls were used for background correction. To determine overall cell death levels, the area under the curve (AUC) was determined using Prism 8.4 (GraphPad Inc.).

### Molecular cloning of vectors for transient expression

For transient expression of Flag-*GBP1* following transfection, the ORF was amplified by PCR using Q5 polymerase and cloned into BamHI and EcoRI digested pcDNA3.1(+) vector using Quick Ligation™ kit. pcDNA3-HA-14-3-3β, γ, ε, ζ and σ were a gift from Anne Ridley (University of Bristol, Bristol, UK) (29) and Michael Yaffe (MIT, Boston, USA) (55). To produce N-terminally HA-tagged 14-3-3η and 14-3-3θ in a pcDNA3 vector identical to the other constructs, the pcDNA3-HA backbone was amplified by PCR. 14-3-3η was amplified from pCS2-HA-14-3-3η (Addgene #116887, a gift from Feng-Qian Li & Ken-Ichi Takemaru) (56) and 14-3-3θ from pcDNA3-14-3-3θ-HA (a gift from Anne Ridley) and finally inserts and backbone assembled by Gibson assembly.

### SDS-PAGE, immunoblotting, and gel staining

For immunoblotting, 0.5×10^6^ cells were seeded per well of a 48-well plate, differentiated with PMA, pre-treated, and infected as described above. At the end of treatments, cells were washed with ice-cold PBS and lysed for 5 minutes on ice in 50 μL RIPA buffer (150 mM NaCl, 1% Nonidet P-40, 0.5% sodium deoxycholate, 0.1% SDS, 25 mM Tris-HCl pH 7.4) supplemented with protease inhibitors (Protease Inhibitor Cocktail set III, EDTA free, Merck) and PhosSTOP phosphatase inhibitors (4906845001, Roche). Lysates were cleared by centrifugation at full speed for 15 minutes at 4°C. Sub-cellular fractionation was performed using QProteome Cell Compartment Kit (37502, Qiagen) following the manufacturer’s instructions. BCA assay (Pierce BCA protein assay kit, 23225, Thermo Scientific) was performed to determine protein concentrations. 10 μg of total protein per sample were mixed with Laemmli buffer (#1610737, Biorad) containing 5% DTT and denatured at 95°C for 10 minutes and then run on Bis-Tris gels (Novex, Invitrogen) in MOPS running buffer. For immunoblots of culture supernatants, cells were treated in OptiMEM (1105802, Gibco). Proteins in the supernatants were precipitated with 4 Vol acetone (V800023, Sigma) overnight at -20°C, and pelleted by centrifugation. Pellets were air dried for 10 minutes, re-suspended in 40 μL 2x Laemmli loading dye, denatured and used for immunoblotting.

Following SDS-PAGE, proteins were transferred onto nitrocellulose membranes using the iBlot transfer system (Invitrogen). Depending on the primary antibody used, the membranes were blocked with either 5% BSA (A2058, Sigma) or 5% dry-milk (M7409, Sigma) in TBS-T (0.05% Tween-20) for at least 1 hour at room temperature. Incubation with primary antibodies (Table S6) was performed at 4°C overnight. Blots were developed by washing with TBS-T, probed with 1:5000 diluted HRP-conjugated secondary antibodies, washed again and imaged on a ChemiDoc MP imaging system (Biorad) using ECL (Immobilon Western, WBKLS0500, Millipore). For quantification of protein band intensities, images were quantified using FIJI/ImageJ and normalized to the actin loading control of each membrane.

For silver staining of protein gels, following SDS-PAGE, the gels were washed in ddH_2_O and then silver stained following the manufacturer’s instructions (Silver Stain Plus Kit, 1610449, Biorad). Similarly, washed gels were used for ProQ Diamond staining following the manufacturer’s instructions (Pro-Q™ Diamond Phosphoprotein Gel Stain, P33300, Invitrogen). Stained gels were imaged on a ChemiDoc MP imaging system (Biorad). Similar to chemiluminescence quantification, protein bands from stained gels were quantified using FIJI ImageJ. Coomassie stain of SDS-PAGE gels was performed using InstantBlue® Coomassie Protein Stain (ISB1L, Abcam).

### GBP1 oligomerization assay

To assess GBP1 oligomerization, a protocol previously published for ASC oligomerization (57) was adapted. In brief, cells were seeded and treated as described above but harvested by scraping in ice-cold PBS containing 2 mM EDTA. Next, cell pellets were washed with PBS+2 mM EDTA and re-suspended in 200 μL buffer A (20 mM HEPES-KOH pH 7.5, 10 mM KCl, 1.5 mM MgCl_2_, 1 mM EDTA, 1 mM EGTA, 320 mM sucrose) and lysed by passing the suspension through a 25G needle. Lysates were cleared by centrifugation at 1,800 × g and 4°C for 15 minutes. From this, sample was kept as lysate input control. The remaining supernatant was diluted 2x with buffer A and then 400 μL CHAPS buffer (20 mM HEPES-KOH pH 7.5, 5 mM MgCl_2_, 0.5 mM EGTA, 0.1% CHAPS) was added. Precipitated GBP1 was pelleted by centrifugation at 7,500 × g for 30 minutes. Finally, pellets were re-suspended in 30 μL of CHAPS buffer with 4 mM of disuccinimidyl suberate (DSS, 21655, Thermo Scientific) and left to cross-link for 30 minutes at room temperature, pelleted again by centrifugation at 7,500 × g for 30 minutes and then dissolved in 2x Laemmli loading dye. Samples were then analyzed by immunoblotting.

### Immunoprecipitations

For immunoprecipitations 5×10^6^ cells were seeded in 6-well plates and differentiated, pre-treated and infected as described above. The cells were washed in ice-cold PBS and scraped from the plates. Whole-cell lysates were prepared by adding 500 μL lysis buffer (1% Triton X-100, 20 mM Tris–HCl pH 8.0, 130 mM NaCl, 1 mM DTT, 10 mM sodium fluoride) with added protease and phosphatase inhibitor cocktails and incubation for 15 minutes on ice. Lysates were cleared by centrifugation at 4°C for 10 minutes at full speed.

For Flag and HA immunoprecipitations, cleared lysates were added to Flag(M2)-agarose beads (A2220, Sigma) or Pierce™ Anti-HA Agarose beads (26182, Thermo Scientific) pre-equilibrated by washing three times with lysis buffer. Proteins were captured by incubation on a rotator overnight at 4°C. Beads were washed once with lysis buffer, three times with lysis buffer containing 260 mM NaCl and twice with lysis buffer. Proteins were eluted using 200 ng/mL 3xFlag peptide (F4799, Sigma) or 200 ng/mL HA peptide (I2149, Sigma) in lysis buffer by incubation on an orbital shaker (1,400 rpm) for 2 hours at room temperature.

For immunoprecipitation of endogenous GBP1/GBP1pS156, 2 μg of antibodies against the proteins and 50 μL of protein G sepharose beads (ab193259, abcam) were added per 1 mL of lysates (~2 mg of protein) and incubated on a rotator overnight at 4°C. Beads were washed with lysis buffer five times and proteins eluted by acidification with 50 μL of 0.2 M glycine pH 2.0 followed by an immediate wash with 50 μL of lysis buffer. Input, unbound and elution fractions were kept during immunoprecipitations and analyzed by immunoblotting.

### Semi-quantitative immunoprecipitation

To determine the affinity of GBP1 binding to 14-3-3 proteins, we adapted a previously published protocol for semi-quantitative co-immunoprecipitation (58). For this, HA-tagged 14-3-3 proteins were produced by transfection of HEK293T cells and purified by HA-immunoprecipitation. Proteins were eluted using HA-peptide and concentrated using 10 kDa cutoff Amicon® Ultra-15 Centrifugal Filters (UFC901024, Merck Millipore), exchanging the buffer to binding buffer (25 mM HEPES pH 7.3, 100 mM NaCl, 0.01% Triton X-100, 5% Glycerol, 1 mM DTT). Phosphorylated GBP1pS156 was purified from IFNγ- and Dox-treated THP-1Δ*GBP1*+Tet-Flag-*GBP1* WT cells using the GBP1pS156 antibody, and the protein concentrated with 50 kDa cutoff Amicon® Ultra-15 Centrifugal Filters (UFC905024, Merck Millipore), exchanging the buffer to binding buffer. Next, the purified and phosphorylated Flag-GBP1 was bound to Flag(M2)-agarose beads (A2220, Sigma) by rotating at 4°C overnight, washed with binding buffer and the beads blocked with 50 mM ethanolamine in PBS. Finally, 10 μL of prepared and washed GBP1 bait beads were incubated with different concentrations of purified 14-3-3 proteins for 1 hour at 4°C and then washed five times with binding buffer. GBP1 and bound 14-3-3 proteins were eluted by boiling the beads in 2x Laemmli loading dye. Finally, obtained samples were analyzed by immunoblotting and blots were quantified using FIJI/ImageJ.

### Mass Spectrometry analysis of GBP1 phosphorylation

In vitro kinase assay samples (+ATP and -ATP) were aliquoted in three technical replicates per condition and diluted with 8 M Urea to 25 mM Tris-HCl, 4 M Urea, 5 mM MgCl_2_. Proteins were reduced with 5 mM Tris(2-carboxyethyl)phosphine-hydrochloride (TCEP) for 30 minutes at 37°C and alkylated with 10 mM Iodoacetamide (IAA) for 30 minutes at room temperature in the dark. Subsequently, protein samples were loaded onto a 30 kDa molecular weight cutoff filter (Sartorius, VIVACON 500, VN01H22) and washed thrice with 50 mM ammonium bicarbonate (Ambic) by centrifugation at 14,000 × g. Proteins were digested with GluC (Promega, V1651) in 50 mM Ambic at a protease/protein (w/w) ratio of 1:20 at 37°C overnight. Peptides were eluted by centrifugation at 14,000 × g and the 30 kDa MWCO filter washed once with 50 mM Ambic. Peptides were cleaned with C18 UltraMicroSpin columns (The Nest Group) following the manufacturer’s recommendation and dried in a SpeedVac concentrator (Thermo Scientific).

Peptides were reconstituted in 3% ACN/0.1% FA/H_2_O and 0.5 μg per sample subjected to liquid chromatography tandem mass spectrometry using an nLC1000 coupled to an Orbitrap Fusion Tribrid mass spectrometer (Thermo Scientific). Peptides were loaded onto a 30 cm column (75 μm ID, New Objective) with mobile phase buffer A containing 0.1% FA/H_2_O and separated by reverse-phase liquid chromatography at a ReproSil-Pur 120 A C18-AQ 1.9 μm stationary phase (Dr. Maisch GmbH). Peptides were eluted with mobile phase buffer B, consisting of 99.9% ACN/0.1% FA, starting from 5% B and increasing up to 50% B in 100 minutes. LC-MS/MS measurements were acquired in data-dependent mode. MS1 spectra were recorded from 200-2,000 m/z with a resolution of 120,000 at 200 m/z in the Orbitrap analyzer. MS1 scans were triggered at an AGC target of 1×10^6^ and maximum injection time in auto mode. Within a cycle time of 3 seconds, peptides were iteratively isolated with a 1.3 m/z isolation window and fragmented by electron-transfer dissociation (ETD) with automatic charge-dependent determined ETD parameters. MS2 scans were recorded in the Orbitrap analyzer upon reaching the AGC target of 5×10^4^ with injection time in auto mode. Fragment ions were monitored with a resolution of 60,000 at 200 m/z. Fragmented peptides were dynamically excluded from further analysis for 30 seconds. Ions with charge state unassigned, 1 or > 6 were excluded from fragmentation.

DDA raw data were processed using MaxQuant (v1.5.6) (59). Spectra were searched against the sequences for recombinant GBP1, PIM1 as well as the proteome sequences of *Escherichia coli* (strain B / BL21-DE3) (tax ID: 469008) and *Trichoplusia ni* (tax ID: 7111), all retrieved from UniProtKB (accessed on 30.05.2021), and common contaminations. Default parameters of MaxQuant were applied, while choosing the protease GluC, allowing 4 miss cleavages, setting the variable modifications to Oxidation (M), Acetyl (Protein N-term) and Phospho (STY), and fixed modification to Carbamidomethyl (C). FDR of 0.01 was controlled at peptide spectrum match, PTM site, and protein level by MaxQuant. Phosphosite localization was automatically calculated by PTM Score of Andromeda within the MaxQuant proteomic suite. Phosphosite data were further analyzed with Perseus (v1.6.10.50). Phosphosites were filtered for minimum localization probability of >0.75 and minimum three measurements in total across all conditions. Peaklist of ETD-activated MS/MS spectrum for GBP1pS156, preprocessed by Andromeda/MaxQuant, was exported from the MaxQuant viewer, and annotated with Interactive Peptide Spectral Annotator (60), considering c-, z•-, and y-type fragment ions.The mass spectrometry proteomics data have been deposited to the ProteomeXchange Consortium via the PRIDE (61) partner repository with the dataset identifier PXD030010.

### Identification of GBP1 interacting proteins by mass spectrometry

10^7^ THP-1Δ*GBP1*+Tet-Flag-*GBP1* cells were seeded in 6-well plates, differentiated, pre-treated with IFNγ and Dox and infected with Tg. Cells were washed in ice-cold PBS, scraped from the plates, pelleted by centrifugation and washed in PBS. Whole-cell lysates were prepared by adding 500 μL lysis buffer (1% Triton X-100, 20 mM Tris–HCl pH 8.0, 130 mM NaCl, 1 mM DTT, 10 mM sodium fluoride) with added protease and phosphatase inhibitor cocktails and incubation for 15 minutes on ice. Lysates were cleared by high-speed centrifugation and used for Flag-immunoprecipitation. The samples were run on a 12% Bis-Tris polyacrylamide gel until the running front had entered the gel ~5 mm. Samples were excised from the gel, de-stained with 50% acetonitrile/50 mM ammonium bicarbonate, reduced with 10 mM DTT, and alkylated with 55 mM IAA. Following alkylation, proteins were digested with 250 ng of trypsin overnight at 37°C and the peptides extracted in 2% formic acid, 1% acetonitrile and speed vacuum dried.

The peptides were reconstituted in 50 μL 0.1% TFA prior to analysis and loaded on a 50 cm EASY-Spray™ column (75 μm inner diameter, 2 μm particle size, Thermo Fisher Scientific), equipped with an integrated electrospray emitter. Reverse-phase liquid chromatography was performed using the RSLC nano U3000 (Thermo Fisher Scientific) with a binary buffer system at a flow rate of 275 nL/min. Buffer A was 0.1% FA, 5% DMSO, and buffer B was 80% acetonitrile, 0.1% FA, 5% DMSO. The samples were run on a linear gradient of buffer B (2 - 30%) in 95.5 minutes. The nano LC was coupled to an Orbitrap Fusion Lumos mass spectrometer using an EASY-Spray™ nano source (Thermo Fisher Scientific). The Orbitrap Fusion Lumos was operated in DDA mode acquiring MS1 scan (R=120,000) in the Orbitrap, followed by HCD MS2 scans in the Ion Trap. Top Speed acquisition algorithm with a cycle time of 3 seconds was used to determine the number of selected precursor ions for fragmentation. The dynamic exclusion was set at 30 seconds. For ion accumulation the MS1 target was set to 4×10^5^ ions and the MS2 target to 2×10^3^ ions. The maximum ion injection time utilized for MS1 scans was 50 ms and for MS2 scans was 300 ms. The HCD normalized collision energy was set at 28 and the ability to inject ions for all available parallelizable time was set to “true”.

Orbitrap .RAW files were analyzed by MaxQuant (version 1.6.0.13), using Andromeda for peptide search. For identification, peptide length was set to 7 amino acids and match between runs was enabled. Parent ion and tandem mass spectra were searched against UniprotKB *Homo sapiens* (tax ID: 9606) and Tg (tax ID: 1080348) databases. For the search the enzyme specificity was set to trypsin with maximum of two missed cleavages. The precursor mass tolerance was set to 20 ppm for the initial search (used for mass re-calibration) and to 6 ppm for the main search. Product mass tolerance was set to 20 ppm. Fixed modification was defined as cysteine carbamidomethylation. Methionine oxidation and N-terminal protein acetylation were searched as variable modifications. The datasets were filtered on posterior error probability to achieve 1% FDR on protein level. Quantification was performed with the LFQ algorithm in MaxQuant using three replicate measurements per experiment. Results were compared against the CRAPome database (62) to eliminate common contaminants of Flag-immunoprecipitations (Data S3-4). The mass spectrometry proteomics data have been deposited to the ProteomeXchange Consortium via the PRIDE (61) partner repository with the dataset identifier PXD029463.

### In vitro kinase assay

To determine in vitro kinase activity of PIM1 (PV3503, Thermo), PIM2 (PV3649, Thermo), PIM3 (A30516, Thermo) and Akt1/PKB (P2999, Thermo) against GBP1 (ab114960, abcam), the recombinant proteins were mixed with 100 μM ATP (PV3227, Thermo) in kinase buffer (50 mM Tris-HCl pH 7.4, 10 mM MgCl_2_) and allowed to incubate for 30 minutes at 30°C. Reactions were stopped by addition of Laemmli buffer containing 5% DTT and denaturing at 95°C for 5 minutes prior to analysis by immunoblotting or silver stain.

### GTPase assay

To determine GTPase activity, 2 μM GBP1, in vitro phosphorylated GBP1 or purified GBP1:14-3-3σ complex was incubated with 1 mM of GTP in reaction buffer (40 mM Tris pH 7.5, 80 mM NaCl, 8 mM MgCl_2_, 1 mM EDTA) at room temperature. Concentration of free phosphate product was subsequently determined using Malachite Green Phosphate Assay Kit (MAK307, Sigma) by measuring absorbance at 620 nm and interpolating a phosphate standard curve.

### Antibody binding assay

ELISA plates were coated with 1 μM phosphorylated or unphosphorylated GBP1 peptides in 100 μL coating buffer (50mM carbonate/bicarbonate pH 9.6) at 4°C overnight. Plates were washed with TBS-T and blocked with 1% BSA in TBS-T for 1 hour at room temperature. Plates were washed again and GBP1pS156 antibody added in different dilutions in blocking buffer and left to bind at 4°C overnight. Plates were washed and probed with HRP-conjugated anti-rabbit secondary antibody, and developed with TMB solution (N301, Thermo Scientific) for 10 minutes at room temperature. Reaction was quenched by addition of 2 N H_2_SO_4_ and absorption quantified on a plate reader (Fluostar Omega, BMG Labtech).

### Isothermal titration calorimetry (ITC)

ITC was performed on a MicroCal VP-ITC. For each titration, 14-3-3σ and GBP1 or previously in vitro phosphorylated GBP1 were dialyzed into reaction buffer (50 mM HEPES pH 7.4, 150 mM KCl). Titrations were performed at 25°C with serial injections of 7 μL aliquots of 14-3-3σ (125/250 μM) into a solution of GBP1 or GBP1pS156 (12.5/25 μM, respectively) in the calorimeter cell (volume 1.3 mL). Data were analyzed with ORIGIN software (MicroCal).

### Fixed immunofluorescence microscopy

For imaging 0.25×10^6^ cells were seeded on coverslips in 24-well plates. Following differentiation, treatments and infection, cells were washed three times with warm PBS prior to fixation and then fixed with 4% methanol-free formaldehyde (28906, Thermo Scientific) for 15 minutes at room temperature. Fixed cells were washed with PBS and kept at 4°C overnight. Fixed specimens were permeabilized with PermQuench buffer (0.2% (w/v) BSA and 0.02% (w/v) saponin in PBS) for 30 minutes at room temperature and then stained with primary antibodies for one hour at room temperature. After three washes with PBS, cells were incubated with secondary antibody and 1 μg/mL Hoechst 33342 (H3570, Invitrogen) diluted in PermQuench buffer for 1 hour at room temperature. Coverslips were extensively washed with PBS, once with ddH_2_O and mounted using 5 μL Mowiol. Specimens were imaged on a Leica SP5-inverted confocal microscope at 100x magnification.

### High-throughput imaging

For infection analysis, 5×10^4^ THP-1s were seeded per well of a black-wall, clear bottom 96-well imaging plate (Thermo Scientific), differentiated, treated, and infected as described above. Following fixation with 4% methanol-free formaldehyde, specimens were permeabilized and stained with 1 μg/mL Hoechst 33342 and 2 μg/mL CellMask™ Deep Red plasma membrane stain (C10046, Invitrogen) at room temperature for 1 hour. After staining, the specimens were extensively washed with PBS and kept in for imaging.

For recruitment analysis, the cells were prepared as described above, but were seeded on black-wall, glass bottom 96-well imaging plates CG 1.0 (130-098-264, MACS Miltenyi). After fixation, cells were permeabilized and stained with primary antibody diluted in PermQuench buffer for 1 hour at room temperature. After three washes with PBS, cells were incubated with the appropriated secondary antibody and 1 μg/mL Hoechst 33342 diluted in PermQuench buffer for 1 hour at room temperature. Then, the specimens were extensively washed with PBS and kept in PBS for imaging.

Imaging for infection analysis used a Cell Insight CX7 High-Content Screening Platform (Thermo Scientific) or a Celldiscoverer 7 (Zeiss) using 10x magnification. For recruitment analysis, plates were imaged on an Opera Phenix High-Content Screening System (Perkin Elmer) or a Celldiscoverer 7 (Zeiss) using 20x/40x magnification. Following image acquisition, the images were fed into the HRMAn analysis pipeline (63, 64).

### Vacuole and parasite breakage assay

For quantification of Tg vacuole integrity, cells seeded in black-wall 96-well imaging plates were infected and treated as described above. One hour prior to fixation, 1 μg/mL HCS CellMask™ Deep Red (H32721, Invitrogen) was added to the culture medium to load the cytosol of host cells with this fluorescent dye. Following fixation and staining with Hoechst 33342, plates were imaged at 20x magnification on a Celldiscoverer 7 (Zeiss). Fluorescence of the dye within detected Tg vacuoles was then analyzed using HRMAn. For analysis of parasite disruption, cells expressing GFP_11_ fragment were infected with type II (Pru) TgΔ*Hpt*+GFP_1-10_ expressing parasites. Following the infection time course, cells were fixed, stained with Hoechst and anti-Tg-SAG1, imaged and analyzed for GFP fluorescence using HRMAn, as described previously (13).

### Fluorescence recovery after photobleaching

Quantification of GBP1 recruitment dynamics was performed using fluorescence recovery after photobleaching (FRAP). For this, 0.1×10^6^ THP-1 cells were seeded per well of an 8-well ibidi μ-slide (80826, ibidi) differentiated, treated and infected with Tg as described above. At 2 hours p.i. the specimens were moved to a Leica SP5-inverted confocal microscope. The experiment was performed using the LAS software FRAP-wizard with parameters: 256x256 px, 1,400 Hz line frequency scan speed with bidirectional scan, 2 AU pinhole size and bleaching laser power at 100%. Following selection of the bleach area on Tg vacuoles the experiment was performed with the following time course: 10 frames at 120 ms pre-bleach, 1 frame at 120 ms bleach, 100 frames at 120 ms post-bleach I, 10 frames at 1 second post-bleach II and 10 frames at 5 seconds post-bleach III. Following acquisition, data was double normalized (65) to correct for acquisition bleaching. Finally, using curve fit, the half-time of recovery was determined as measurement for mobility of the molecules.

### Imaging quantifications

Most quantifications of images were performed using HRMAn (63, 64). HRMAn was used for quantification of Tg-growth, Golgi fragmentation, recruitment of proteins to pathogen vacuoles and measurement of cell shapes, sizes and fluorescence properties. The Pearson’s correlation coefficient for colocalization analysis was computed using Coloc2 FIJI plugin (66). Average distance of GBP1 aggregates to the nucleus was determined using AggreCount (67). Golgi localization of GBP1 was quantified by determining the MFI of GBP1 in the GM130^+^ area and divided by the MFI of the respective cell cytosol to normalize for uneven expression.

### Recombinant protein expression and purification

*GBP1* and *14-3-3σ* ORFs were amplified from pLenti-Tet-*GBP1* and pLenti-Tet-*14-3-3σ* and ligated into NdeI and BamHI digested pET15d vector, pre-tagged N-terminally with an MBP-8xHis-3C or 6xHis-3C sequence using Gibson assembly to obtain pET15d-MBP-8xHis-3C-*GBP1* and pET15d-6xHis-3C-*14-3-3σ*, respectively. Plasmids were transformed into BL21 *E*. *coli*. Bacteria from a single colony were picked and grown in a 10 mL LB+Ampicillin (EU0400-D, Euromedex) starter culture overnight. Starter cultures were used to inoculate 12 L of LB+Ampicillin cultures (1:1000 dilution) which were grown to OD_600_ ~ 0.9. Protein expression was induced by addition of 1 μM β-D-1-thiogalactopyranoside (IPTG; EU0008-B, Euromedex). GBP1 was expressed for 16 hours at 18°C and 14-3-3σ at 30°C for 4 hours while shaking at 150 rpm.

Bacteria were harvested by centrifugation, washed with PBS, re-suspended in 100 mL of lysis buffer (GBP1: 50 mM HEPES pH 7.8, 30 mM imidazole, 150 mM KCl; 14-3-3σ: 50 mM NaH_2_PO_4_ pH 8.0, 10 mM imidazole; 300 mM NaCl) and lysed by sonication on ice (12 minutes, 1 second pulses with 1 second interruptions). The lysate was cleared by high-speed centrifugation at 20,000 × g for 1 hour at 4°C and cleared lysate loaded onto a 5 mL Ni-NTA column (ab270529, Abcam). The column was washed with 20 mL lysis buffer and bound proteins eluted with a 40 mL gradient from 30 mM to 500 mM imidazole in lysis buffer. Target protein-containing fractions were pooled and dialyzed overnight against size exclusion buffer (SEC buffer: 50 mM HEPES pH 7.8, 150 mM KCl) while also digesting with His-3C protease (Protein Expression and Purification Core Facility, EMBL Heidelberg). His-3C protease and cleaved tags were removed using reverse immobilized metal affinity chromatography Ni-IMAC purification (635660, Takara) and proteins concentrated using 50 kDa cutoff spin column concentrators (Amicon^®^ Ultra).

The full-length PIM1 ORF was amplified from pLenti-Tet-*PIM1* and ligated into BamHI and HindIII digested pLIB vector modified with a N-terminal MBP-TEV-SBP tag using Gibson assembly to obtain pLIB-MBP-TEV-SBP-*PIM1*. The assembled vector was used for Baculovirus generation in SF21 cells (SF21: 11497013, Gibco; SF-900™ II SFM: 11497013, Gibco) as described previously (68, 69). 5 mL Baculovirus was used for transfection of 500 mL Hi5 (*Trichoplusia ni* High Five Cells: B85502, Invitrogen; ExpressFive™ SFM, 10486025, Gibco) culture and incubated at 27°C for 72 hours. Hi5 insect cells were harvested by centrifugation, washed with PBS, re-suspended in lysis buffer (50 mM HEPES pH 8.0, 300 mM NaCl) and lysed by sonication at 4°C (12 minutes, 1 second pulses with 1 second interruptions). The lysate was cleared by high-speed centrifugation at 20,000 × g for 1 hour at 4°C. Cleared lysate was loaded onto pre-equilibrated amylose resin (E8021S, NEB) and left to bind for 4 hours at 4°C. The resin was washed with 10 mL lysis buffer three times and bound PIM1 eluted with 30 mL lysis buffer containing 25 mM maltose. Obtained MBP-TEV-SBP-PIM1 was dialyzed against 50 mM NaH_2_PO_4_ pH 8.0, 300 mM NaCl and 30 mM imidazole buffer overnight while also digesting with His-TEV protease (Protein Expression and Purification Core Facility, EMBL Heidelberg). His-TEV protease and cleaved tags were removed using serial reverse His- and amylose-affinity purification and PIM1 protein concentrated using 30 kDa cutoff spin column concentrators (Amicon® Ultra). Bulk-purified GBP1, PIM1 and 14-3-3σ were further purified by SEC with a HiLoad® 16/600 Superdex® 200 column (Cytiva Lifescience) in SEC buffer.

### GBP1:14-3-3s complex formation and purification

For in vitro reconstitution of the GBP1:14-3-3σ complex, purified GBP1, 14-3-3σ and PIM1 were mixed in a 2:4:1 ratio in complex formation buffer (Tris-HCl pH 7.4, 150 mM KCl, 10 mM MgCl_2_) with addition of 1 mM ATP and incubated at 30°C for 1 hour. Reaction samples were cleared by centrifugation at 20,000 × g for 5 minutes at 4°C and ran over a Superdex 200 Increase 10/300 GL column (Cytiva Lifesciences) in SEC buffer. To further polish for structure analysis, complex-containing fractions were pooled, concentrated with a 100 kDa cutoff spin column concentrator (Amicon^®^ Ultra) and re-purified by SEC. To test the effect of 14-3-3σ-binding on GBP1 dimerization, samples were additionally incubated with 1 mM GTP or with 1 mM GDP + 10 mM AlF_3_ (449628, Merck).

### Single particle cryo-electron microscopy and structure modeling

The GBP1:14-3-3σ complex was prepared as described above. Fractions corresponding to a heterotrimeric complex were vitrified in R1.2/R1.3 UltrAuFoil grids with a 300 mesh (Q350AR13A, Electron Microscopy Sciences) that were glow discharged for 20 seconds at 25 mA and 0.3 bar using a Pelco EasyGlow device. The sample concentration was adjusted to 0.25 mg×mL^-1^ and 2 μL drops in SEC buffer were applied to either side of the grid. Excess sample was removed by blotting using a Vitribot MARK IV at 4°C, 100% humidity for 2 seconds with blotting force 10 and subsequently plunge frozen in liquid ethane. The vitrified particles were imaged using a FEI Titan Krios cryo-TEM at 300 kV at EMBL Heidelberg, equipped with a GIF Quantum K2 direct electron detector and a GIF quantum energy filter (Gatan). In total 14,974 movies were collected in counting mode with 7 exposures per hole at a nominal magnification of 215,000x with a pixel size of 0.638 Å^2^. Each movie was exposed for 8 seconds resulting in a total dose of 65.5 e·Å^-2^ over 40 frames.

All data processing steps were performed using cryoSPARC v3.2.0 unless stated otherwise (70). Imported movies were subjected to motion correction using the cryoSPARC multi-patch motion correction followed by multi-patch CTF estimation. Initially, particles were identified by using automated gaussian picking for elliptical particles with 50×120 Å. Picked particles were extracted 4-fold binned and subjected to extensive 2D classification, identifying 4,129 particles corresponding to the 14-3-3σ/GBP1 complex. The particles were used to train Topaz (71) and re-pick on all micrographs, yielding 420,768 particles. 2D classification identified 29,150 particles. *Ab initio* modelling with three models was used to generate preliminary volumes. Re-extraction followed by a heterogenous refinement using the previously determined initial 3D volumes resulted in a similar particle distribution. Class 3 was used to re-train Topaz for automated particle picking. Identified particles were extracted with 4-fold binning. Subsequent 2D classification identified 138,809 particles which were used to generate 5 new initial models. The particles were re-extracted with full pixel size and subjected to heterogenous refinement with the previously determined 3D volumes. Class 3 yielded a model with 40,112 particles. The corresponding particles were further classified into three *ab initio* models. A class with 18,717 particles was identified and subjected to non-uniform refinement yielding a final cryo-EM map at 5.1 Å.

Densities were generated in CryoSPARC, loaded into ChimeraX (version 0.93) (72), and previously reported crystal structures (PDB: 1F5N for GBP1 and PDB: 1YWT for 14-3-3σ dimer) were fitted using the programs own density fitting tools. The final model was deposited on EMDB with accession number EMD-18149 and the rigid body docked model was deposited in the PDB with accession number (8Q4L).

### Evolutionary analysis of *GBP* genes

Position-Specific Iterated-Basic Local Alignment Search Tool (PSI-BLAST) (73) searches using full-length GBP1 as query of the RefSeq Protein sequence database identified >3,300 GBP1-like proteins from >500 species. Protein sequences were aligned with MAFFT (74), and incomplete data was removed with MaxAlign v1.1 (75). The closest homologue of human GBP1 could be identified based on sequence-homology in 484 species and the respective sequences analyzed for presence of the corresponding phospho-serine and PIM1 recognition motif.

### In silico analysis, data handling and statistics

Data was plotted using Prism 8.4.0 (GraphPad Inc.) and presented as means of multiple experiments with error bars as standard deviation (SD), unless stated otherwise. Significance of results was determined by non-parametric one-way ANOVA, unpaired t-test or two-way ANOVA as indicated in the figure legends. Benjamini, Krieger and Yekutieli false-discovery rate (Q = 5%) based correction for multiple comparisons as implemented in Prism was used when making more than 3 comparisons. Structures of GBP1 were obtained from the PDB database: 1F5N, 1DG3 (76, 77) and images rendered with MacPymol v.1.74 which was also used for B-factor analysis. Molecular graphics and analyses were performed with UCSF ChimeraX. GBP1 phosphorylation-site data was obtained from phosphosite.org (accessed on 4. January 2019). 14-3-3 binding site identification used 14-3-3-Pred (78). Kinase predictions were performed using NetPhos Server3.1 (79) and GPS5.0 (80) and recognition sequence phosphorylation-likelihood values were obtained from PhosphoNET: Kinexus (http://www.phosphonet.ca/). All open-source KNIME workflows used in this work can be found at: https://github.com/HRMAn-Org/HRMAn and on the homepage hrman.org.

## Supplementary Material

Data S1

Data S2

Data S3

Data S4

Data S5

Data S6

Supplementary Material

## Data Availability

All data are available in the main text or the supplementary materials. For underlying data see Data S1-S6. The proteomics raw data has been deposited to PRIDE (PXD029463 & PXD030010). The cryo-EM data has been deposited on EMDB (EMD-18149). All unique/stable reagents generated in this study are available from the lead contact without restriction.

## One Sentence Summary

Phosphorylation of IFNγ-induced GBP1 by the kinase PIM1 guards IFNγ-signaling integrity and promotes bystander cell protection.

An IFNγ-signaling guard mechanism controls antimicrobial GBP1 activity via the kinase PIM1 and protects uninfected bystander cells.
