# Supplementary material for: PIM1 controls GBP1 activity to limit self-damage and to guard against pathogen infection
Source: Science. Author manuscript; Available in PMC 2023 Oct 12. (PMC7615196; doi:10.1126/science.adg2253)

Fig. 1C

Figure as in manuscript:

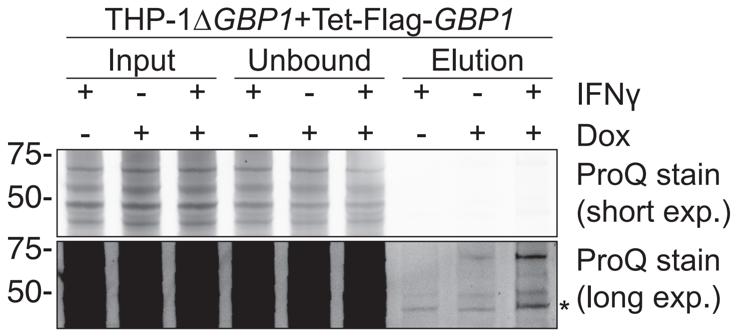

Uncropped gels with indicated size marker and crop area (lane annotation, if excluded from figure):

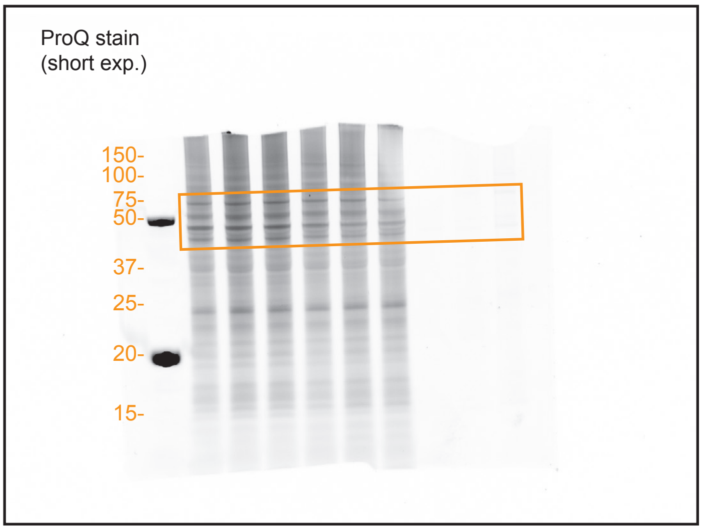

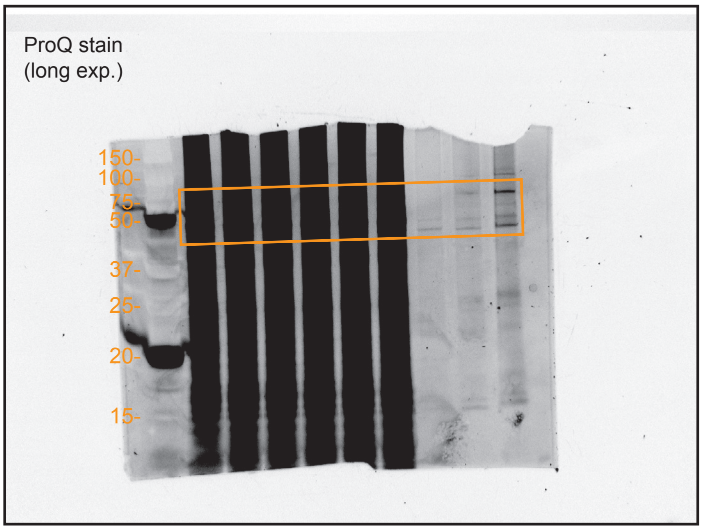

SDS-PAGE gel stained with ProQ diamond gel stain for phospho-protein detection

Fig. 1D

Figure as in manuscript:

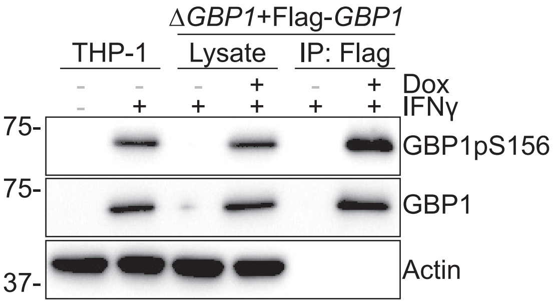

Uncropped gels with indicated size marker and crop area (lane annotation, if excluded from figure):

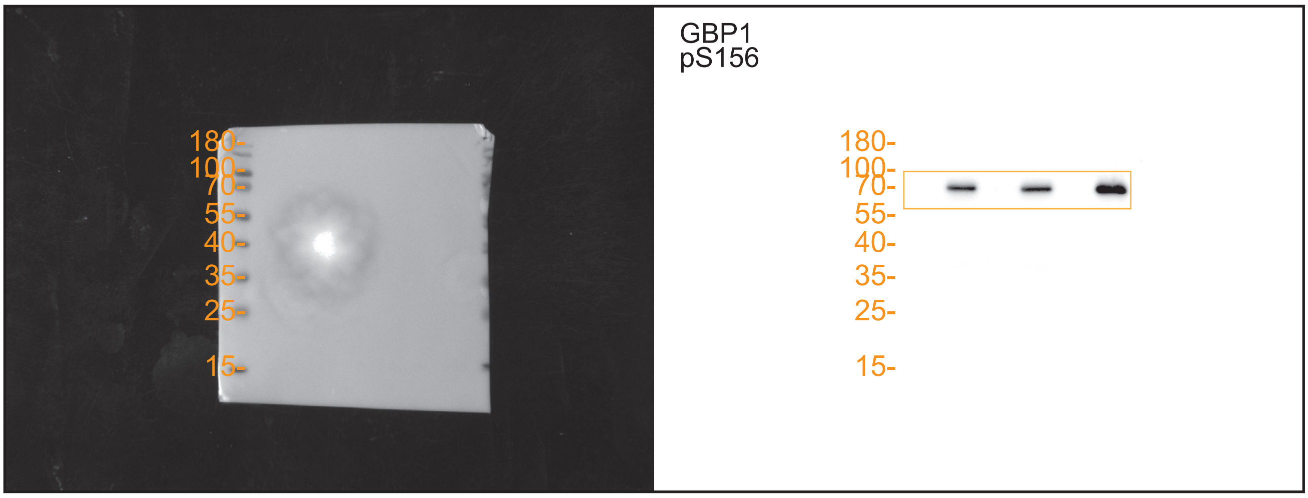

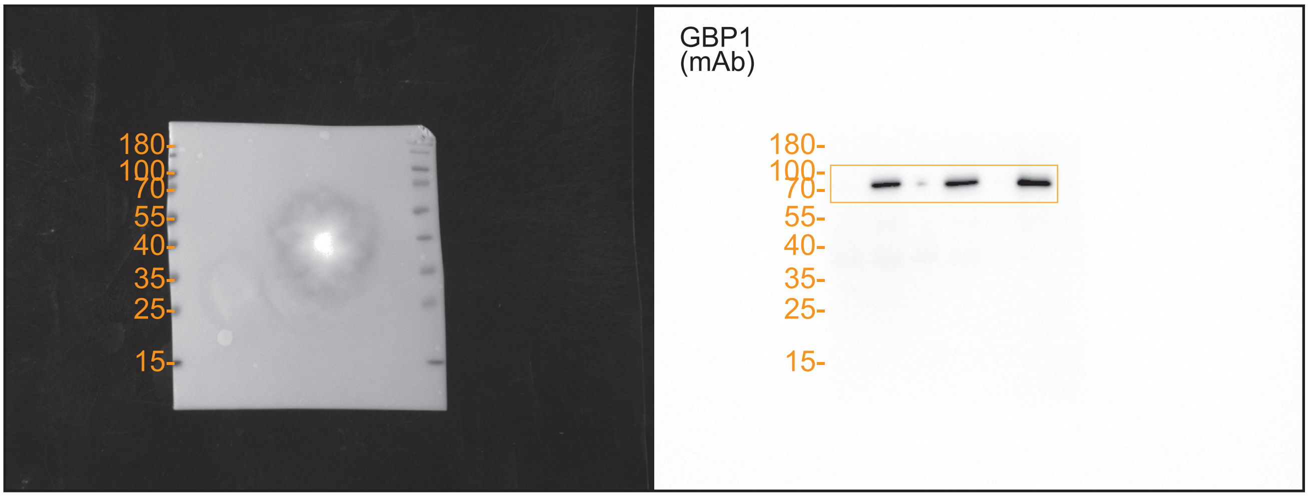

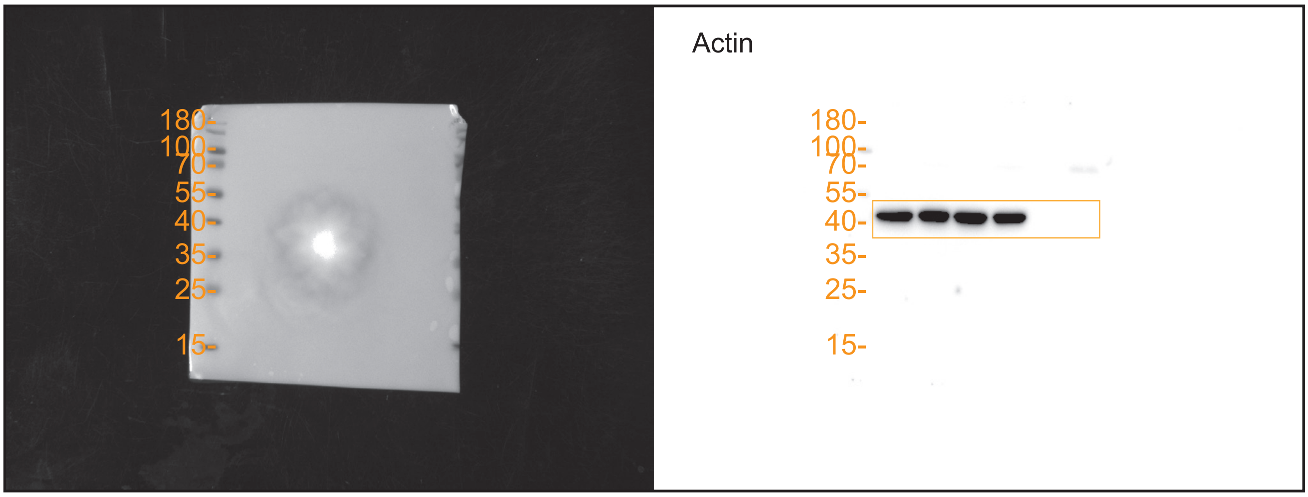

Fig. 1F

Figure as in manuscript:

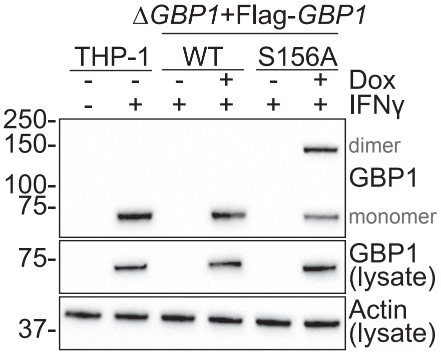

Uncropped gels with indicated size marker and crop area (lane annotation, if excluded from figure):

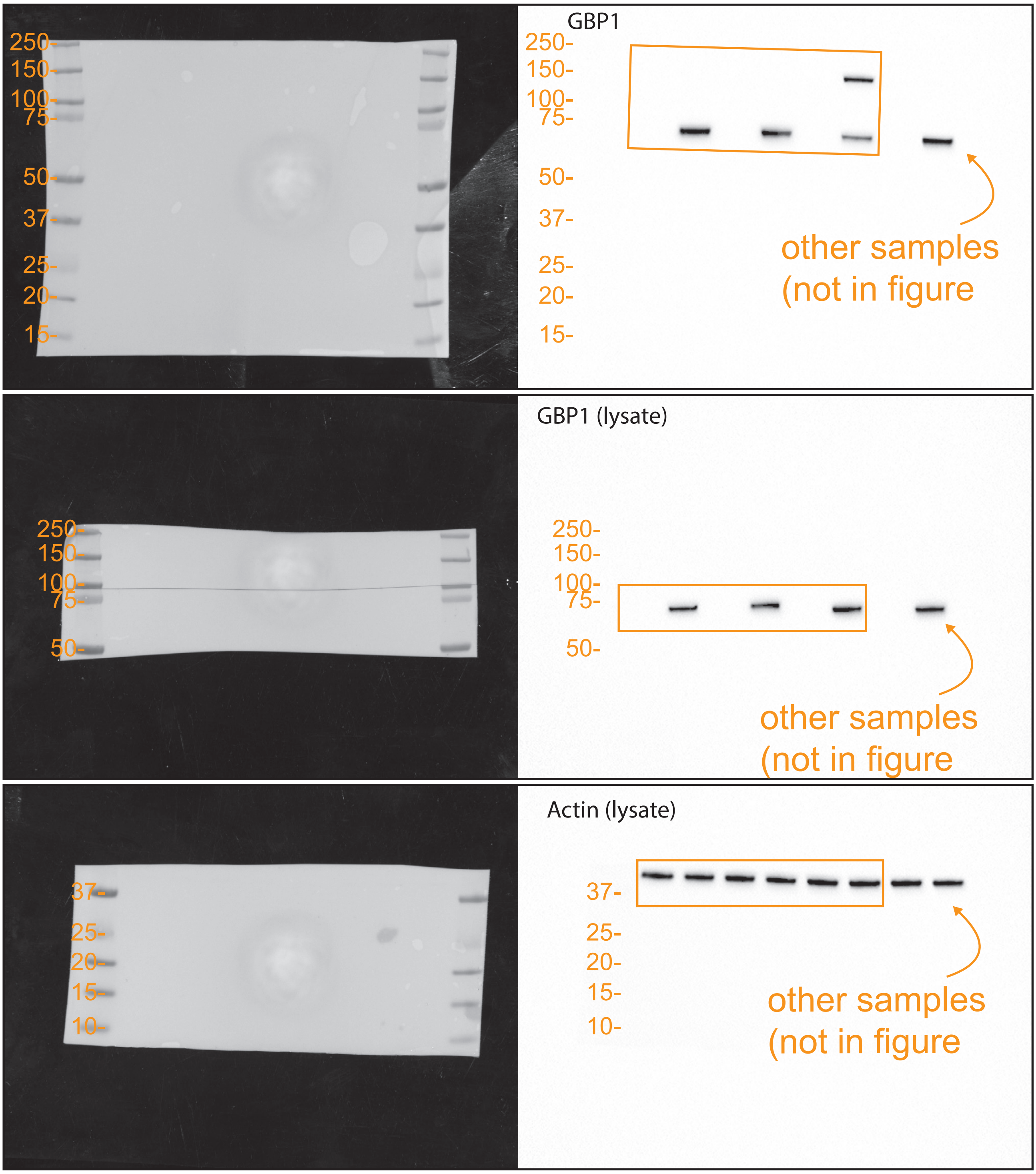

Fig. 1G

Figure as in manuscript:

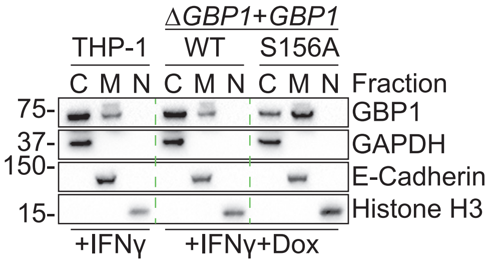

Uncropped gels with indicated size marker and crop area (lane annotation, if excluded from figure):

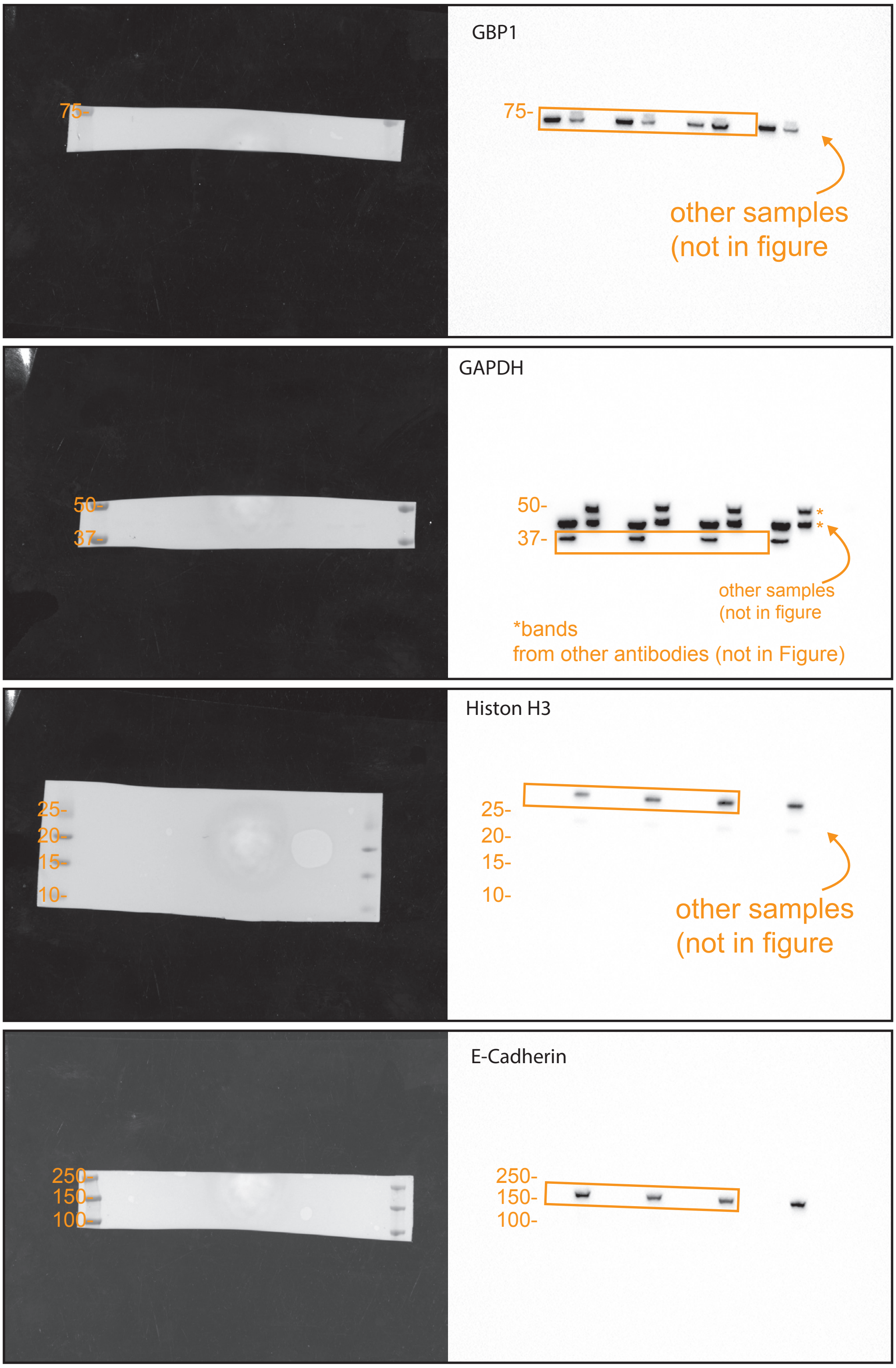

Fig. 2A

Figure as in manuscript:
(Quantifications removed)

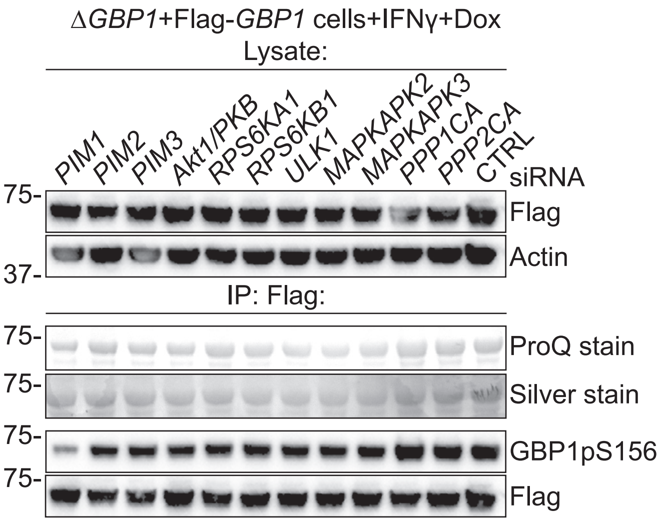

Uncropped gels with indicated size marker and crop area (lane annotation, if excluded from figure):

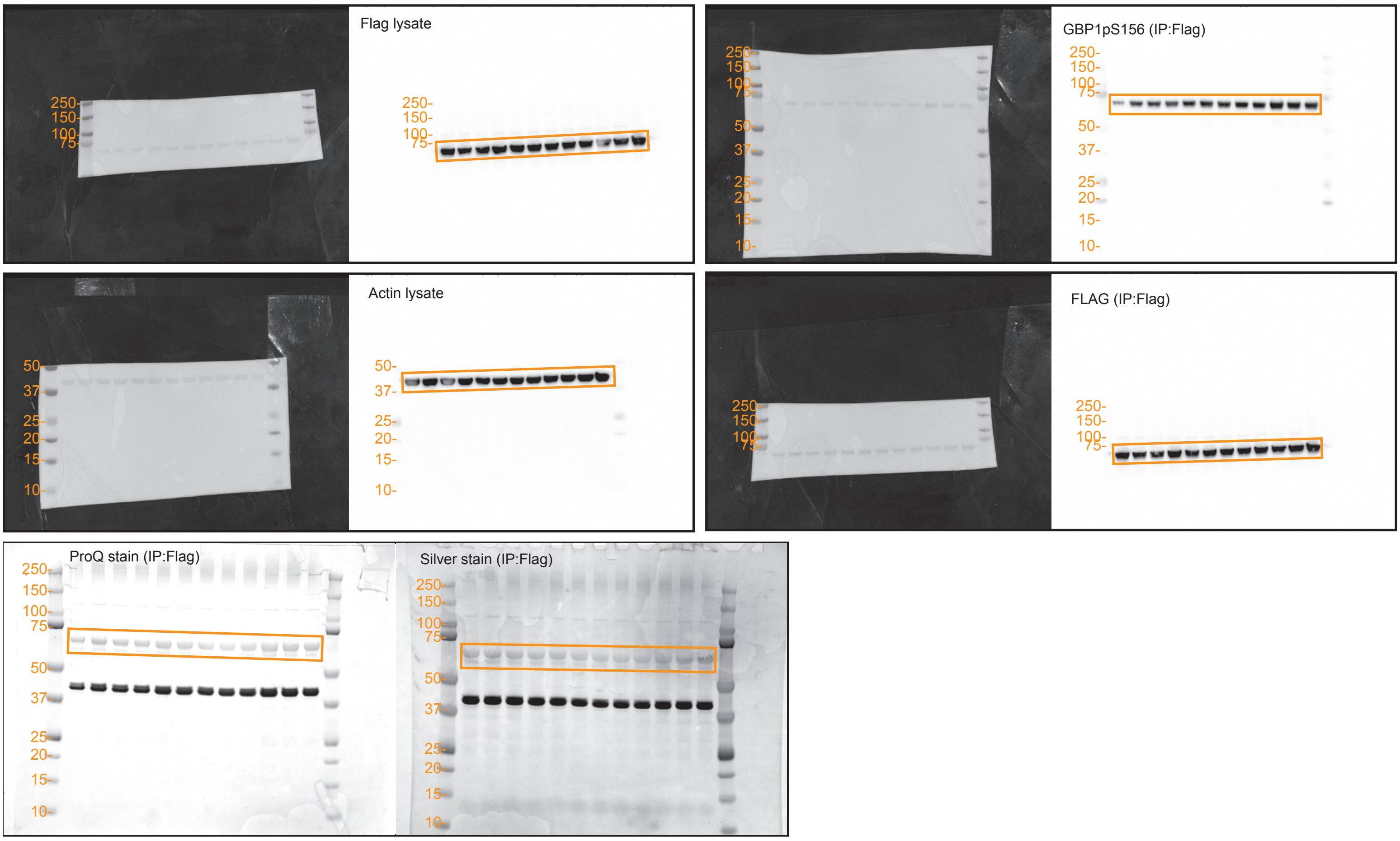

Fig. 2B

Figure as in manuscript:

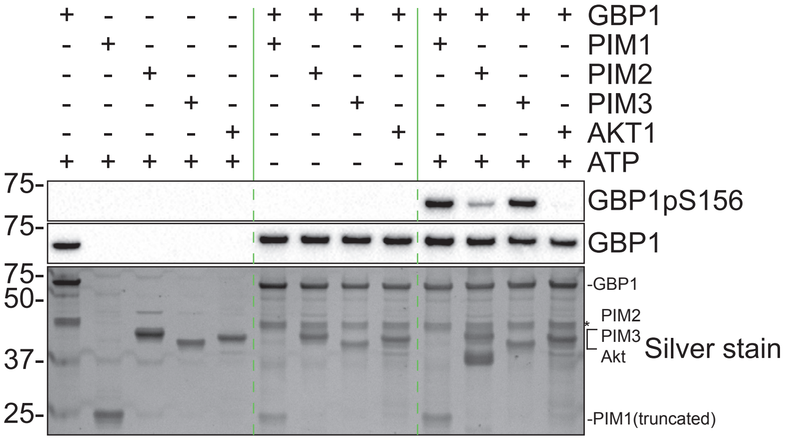

Uncropped gels with indicated size marker and crop area (lane annotation, if excluded from figure):

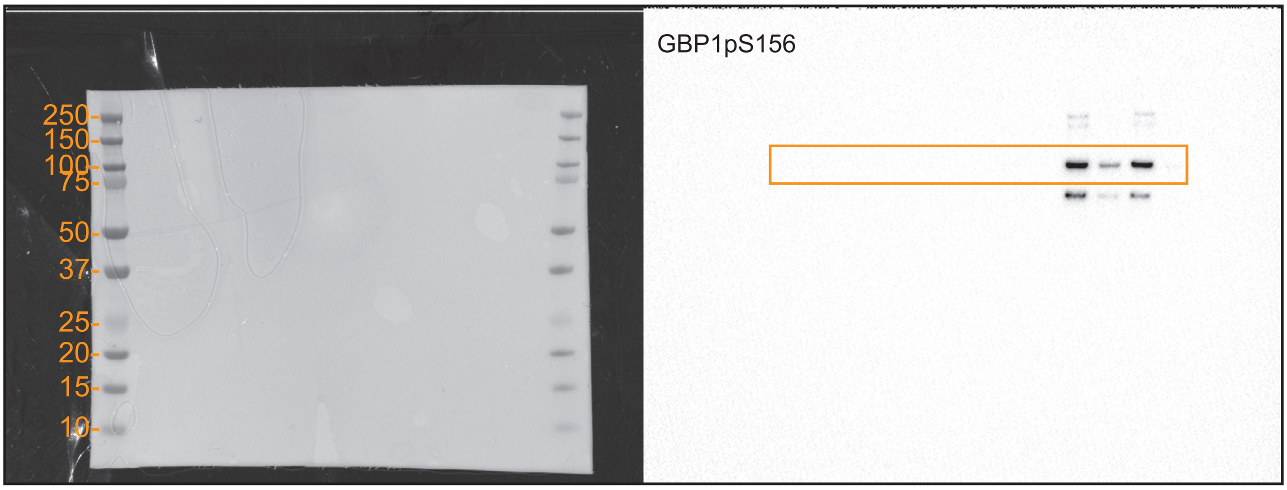

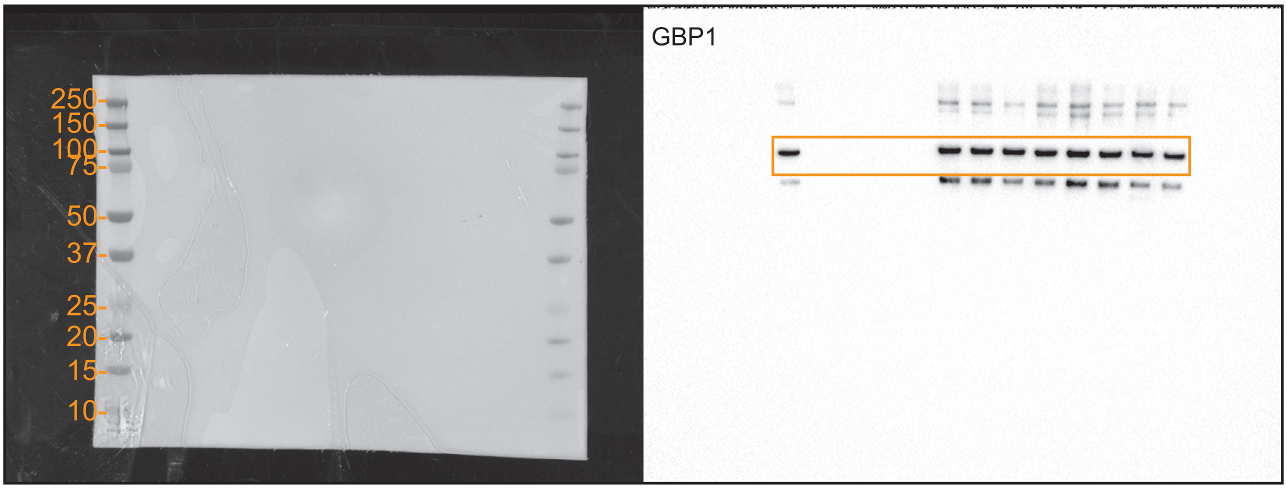

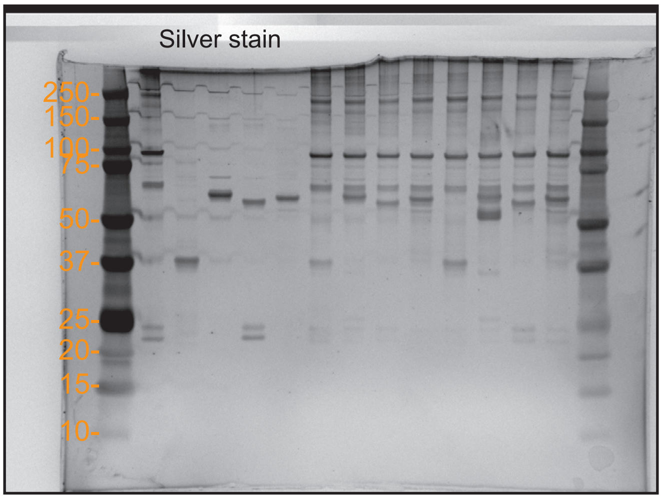

Fig. 2C

Figure as in manuscript:

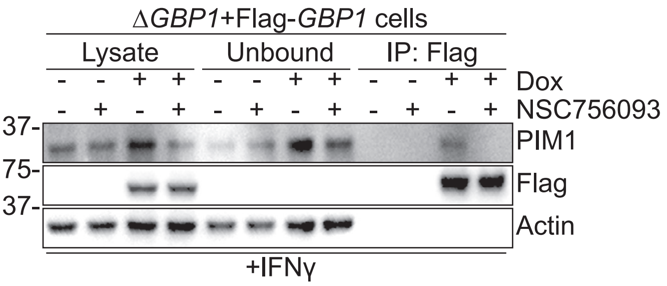

Uncropped gels with indicated size marker and crop area (lane annotation, if excluded from figure):

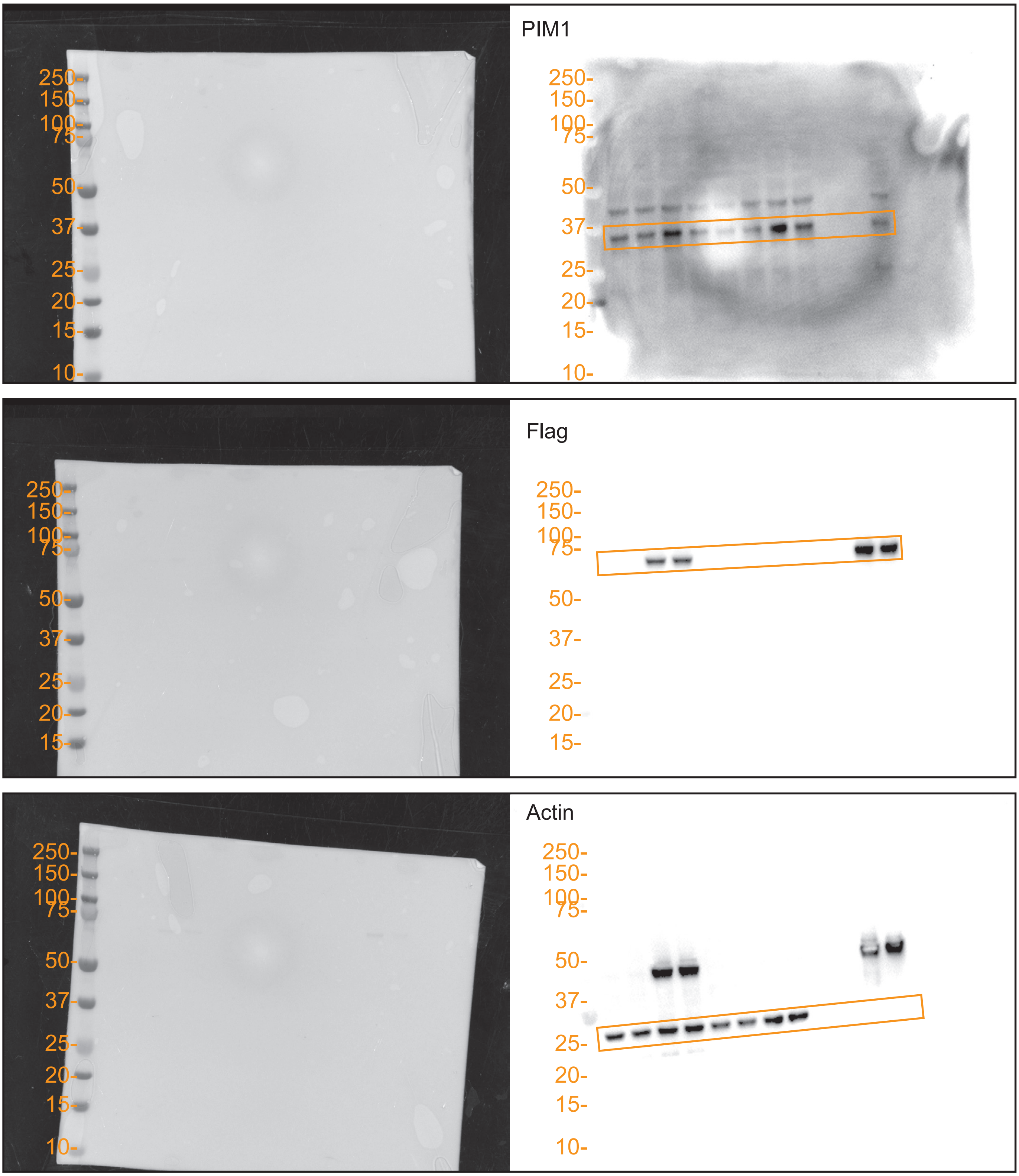

Fig. 2D

Figure as in manuscript:

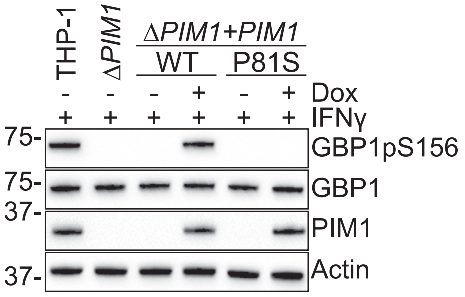

Uncropped gels with indicated size marker and crop area (lane annotation, if excluded from figure):

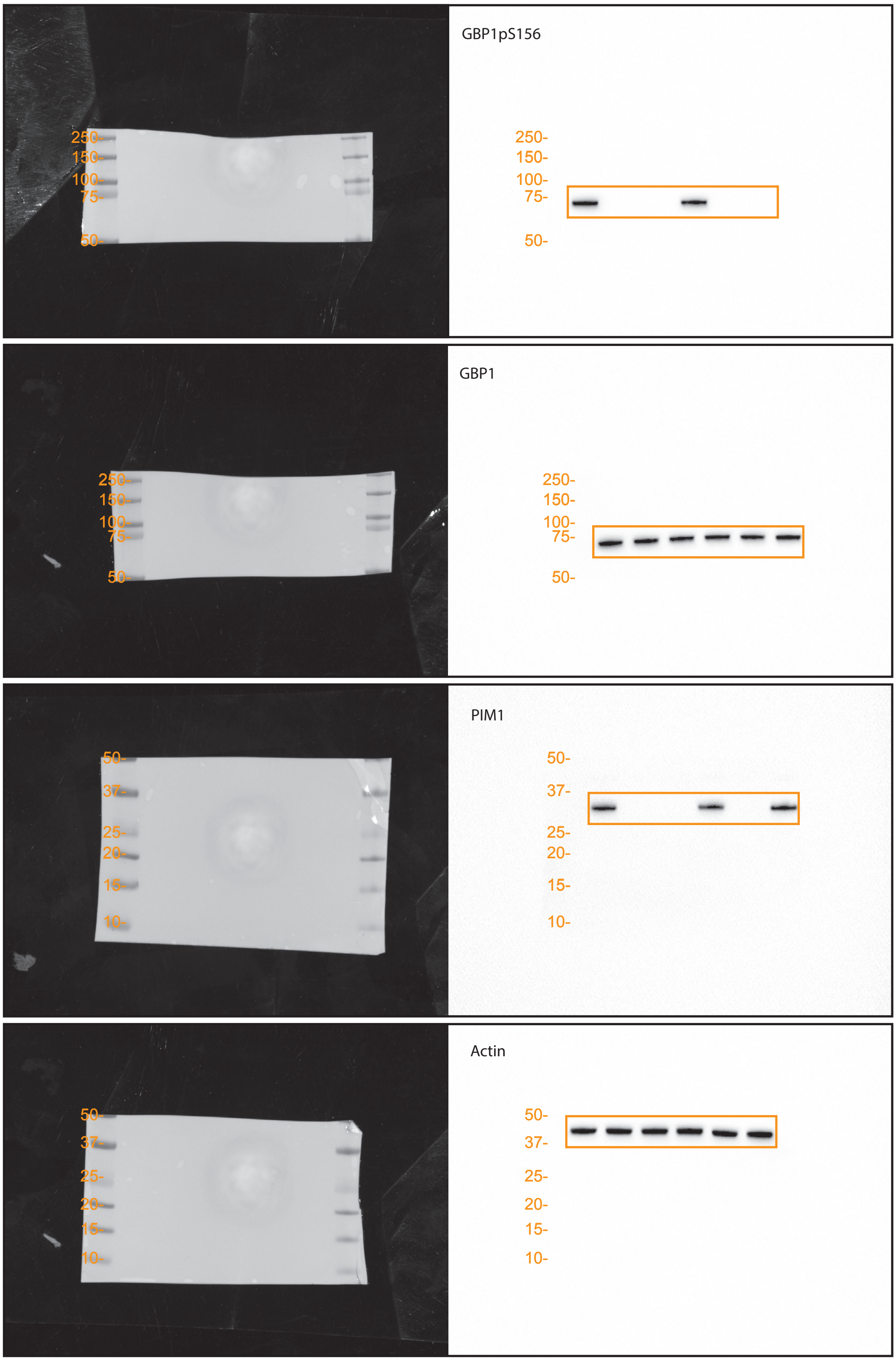

Fig. 2E

Figure as in manuscript:

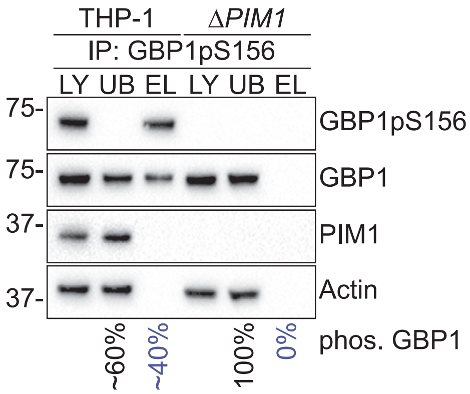

Uncropped gels with indicated size marker and crop area (lane annotation, if excluded from figure):

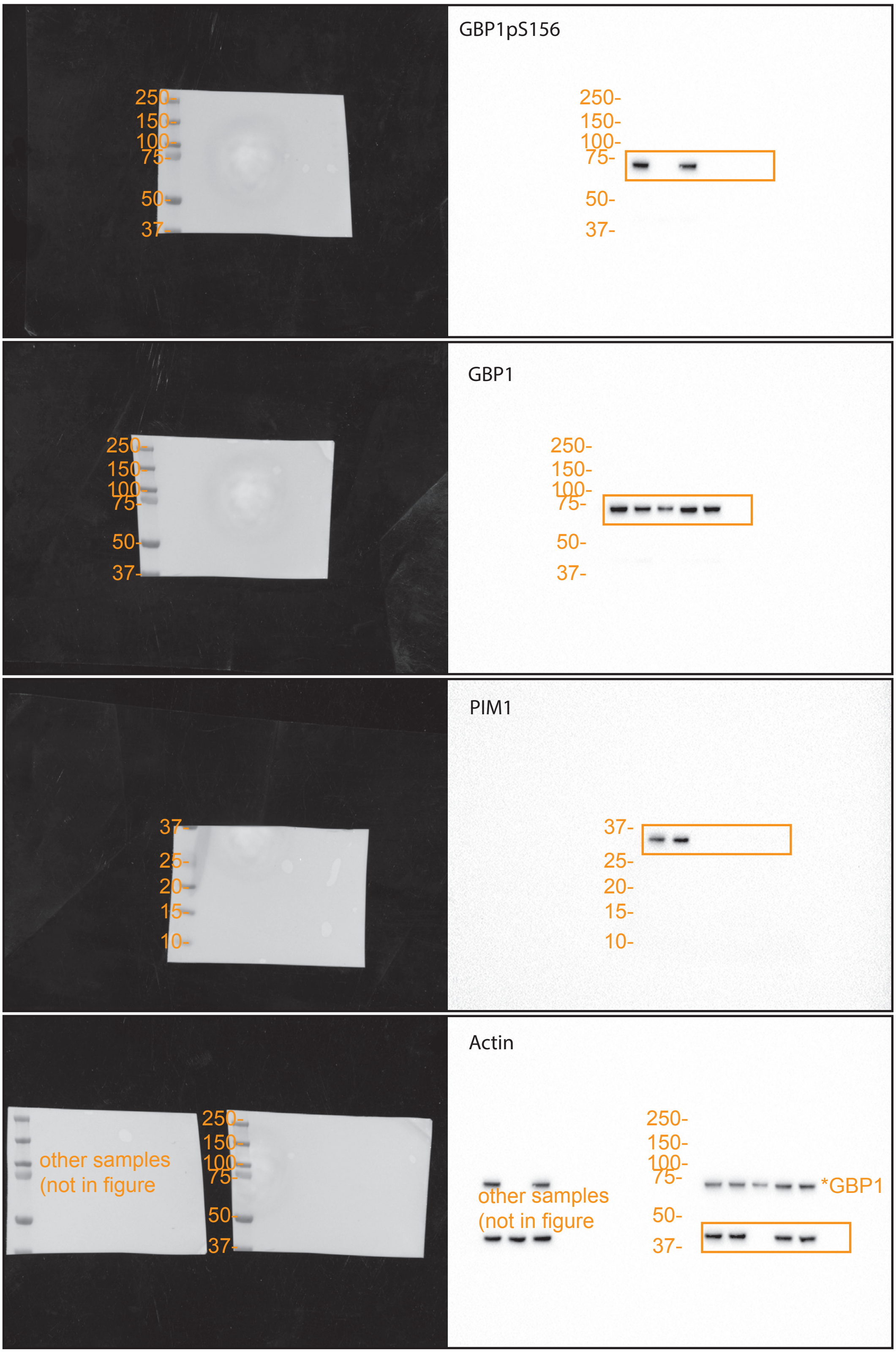

Fig. 3B

Figure as in manuscript:

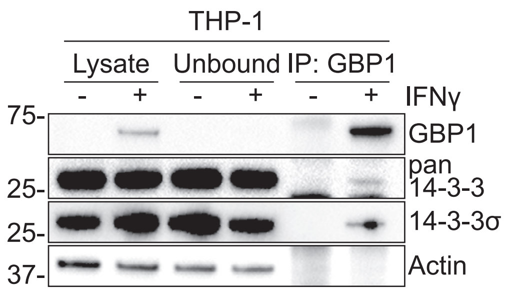

Uncropped gels with indicated size marker and crop area (lane annotation, if excluded from figure):

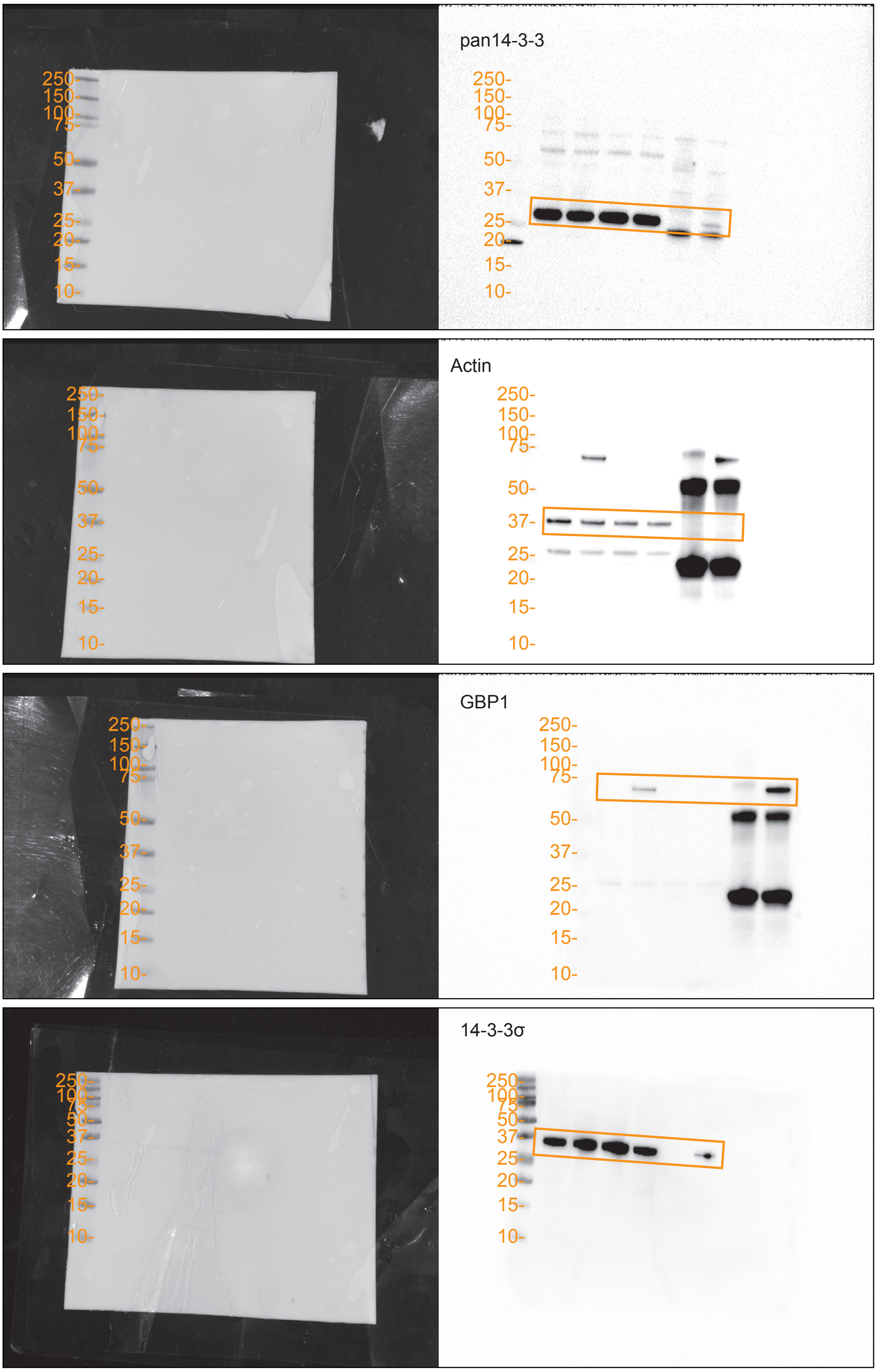

Fig. 4C

Figure as in manuscript:

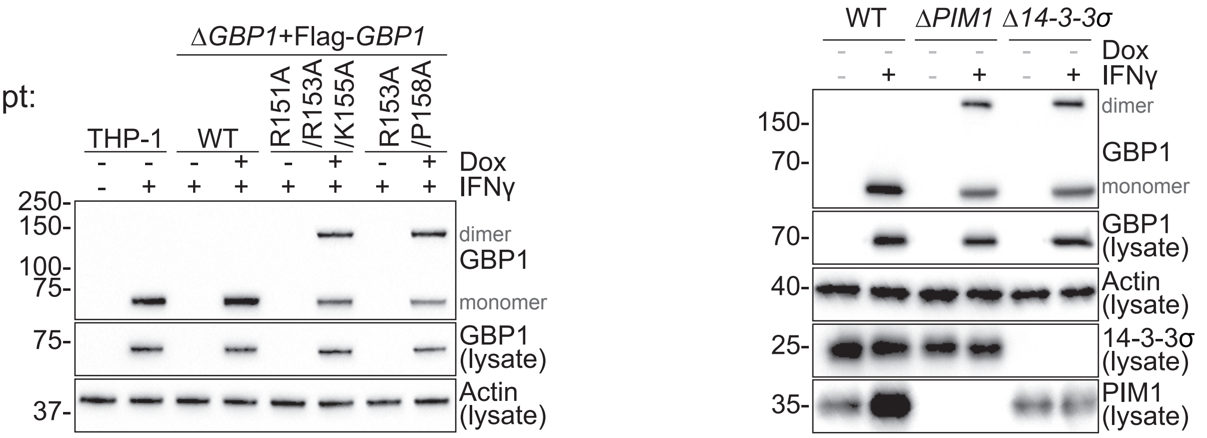

Uncropped gels with indicated size marker and crop area (lane annotation, if excluded from figure):

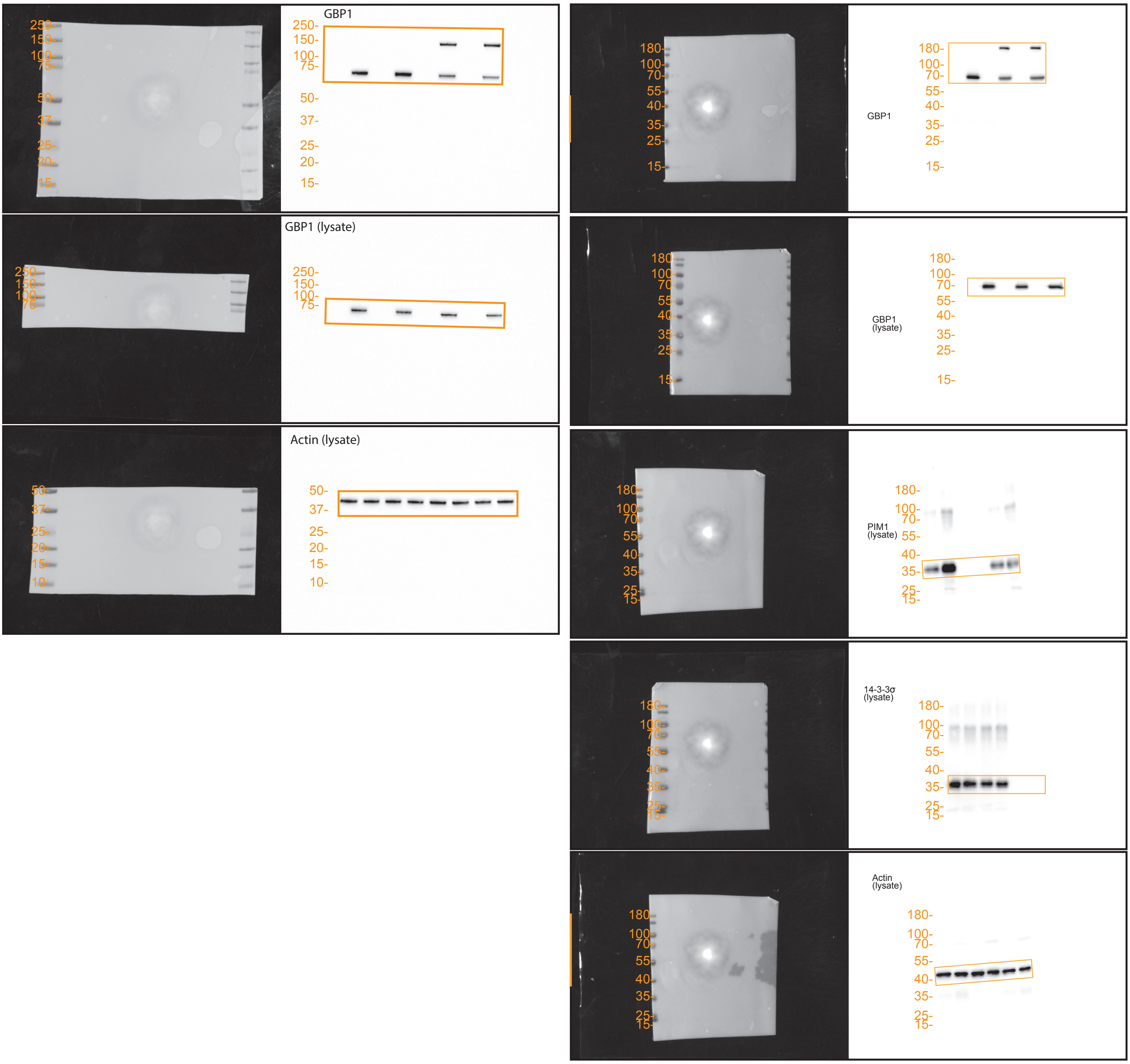

Fig. 4D

Figure as in manuscript:

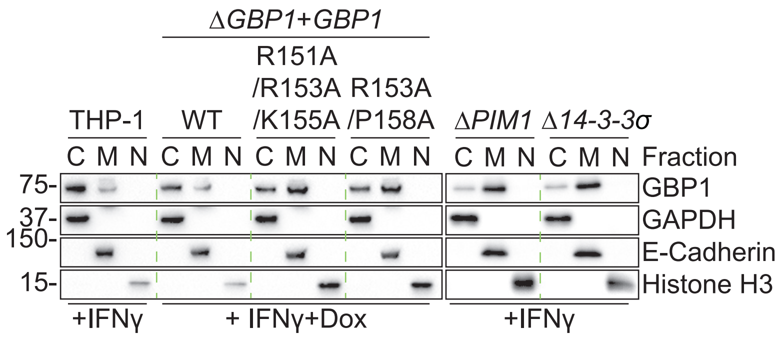

Uncropped gels with indicated size marker and crop area (lane annotation, if excluded from figure):

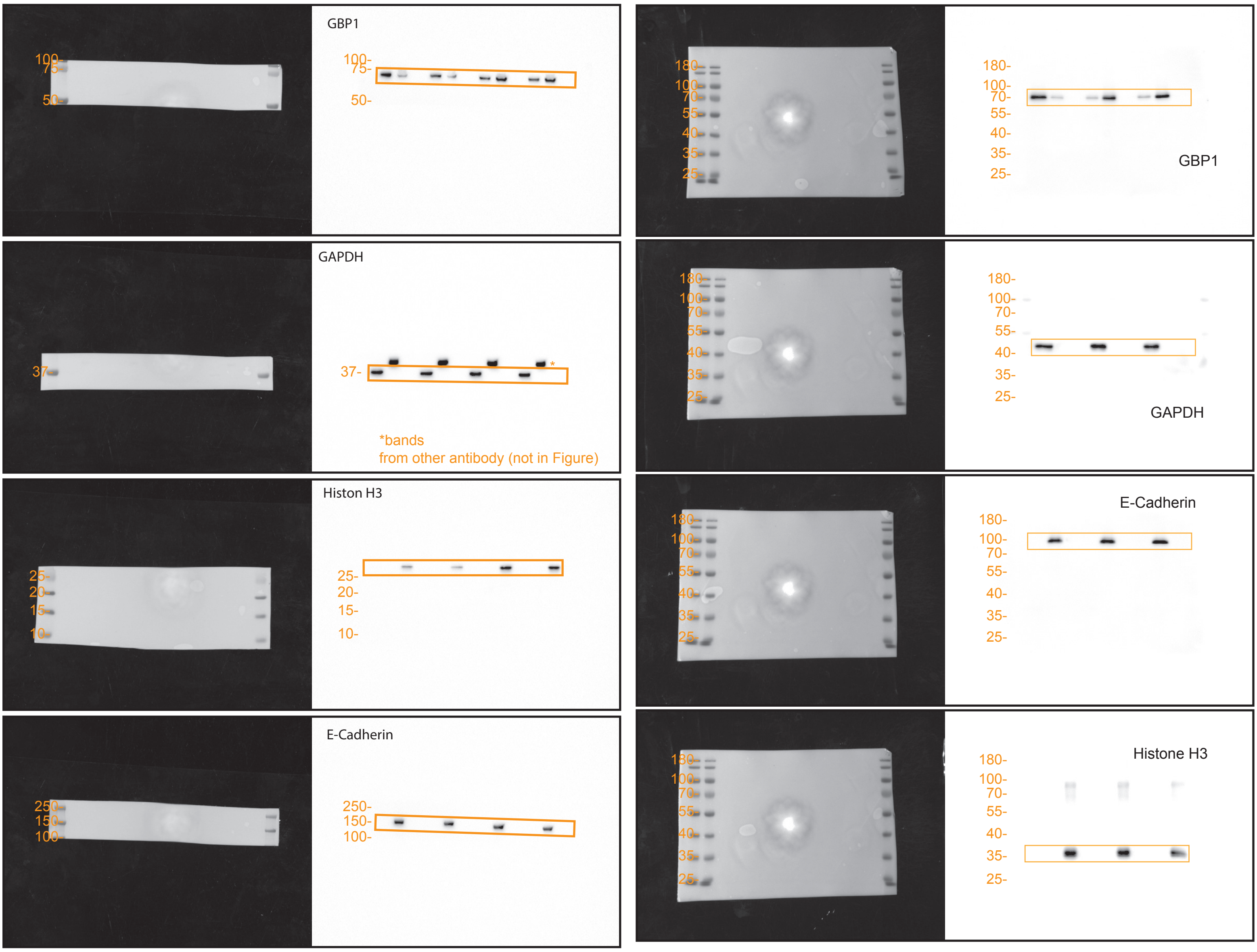

Fig. 5B

Figure as in manuscript:

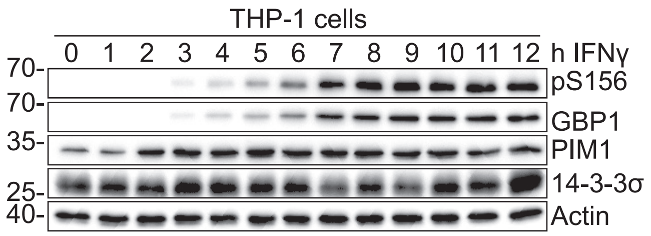

Uncropped gels with indicated size marker and crop area (lane annotation, if excluded from figure):

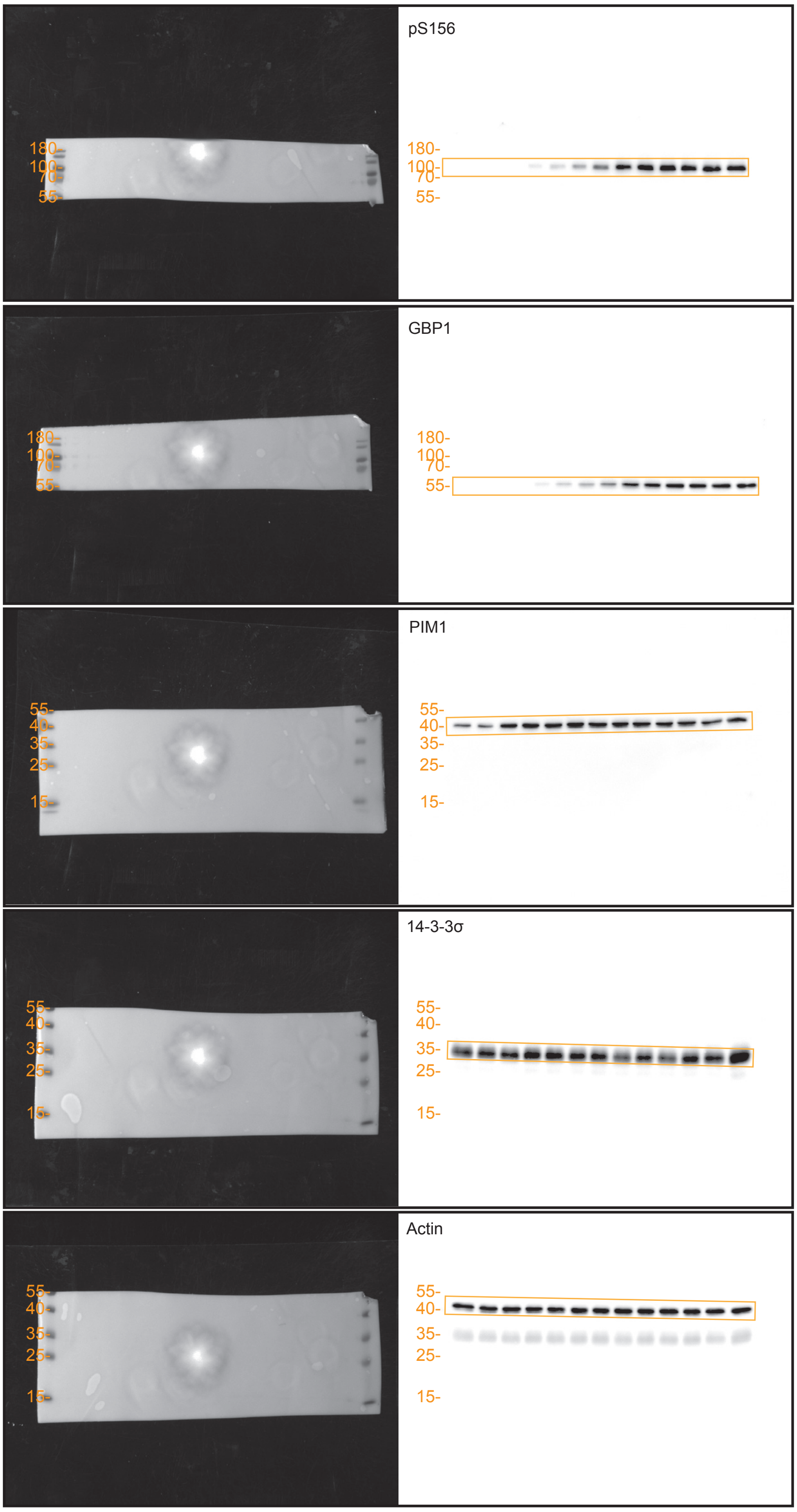

Fig. 5C

Figure as in manuscript:

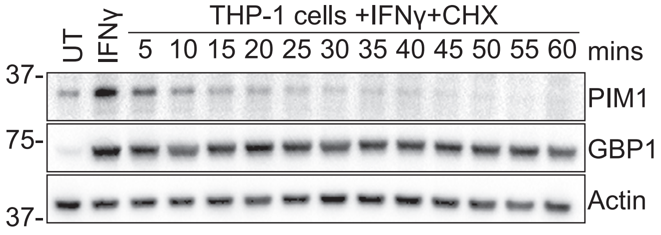

Uncropped gels with indicated size marker and crop area (lane annotation, if excluded from figure):

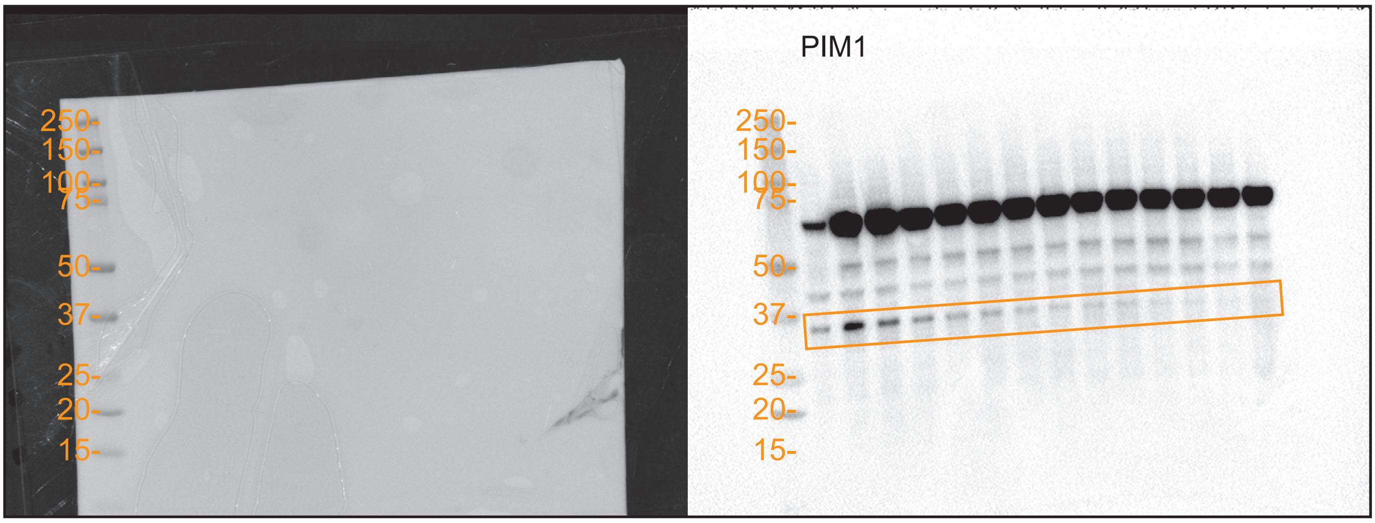

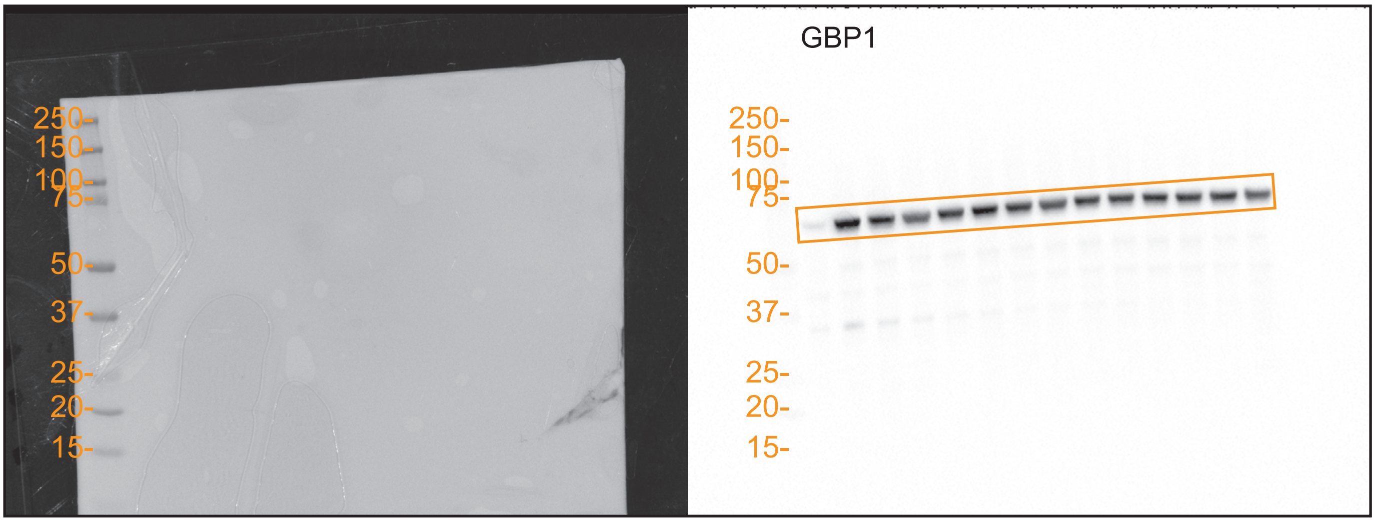

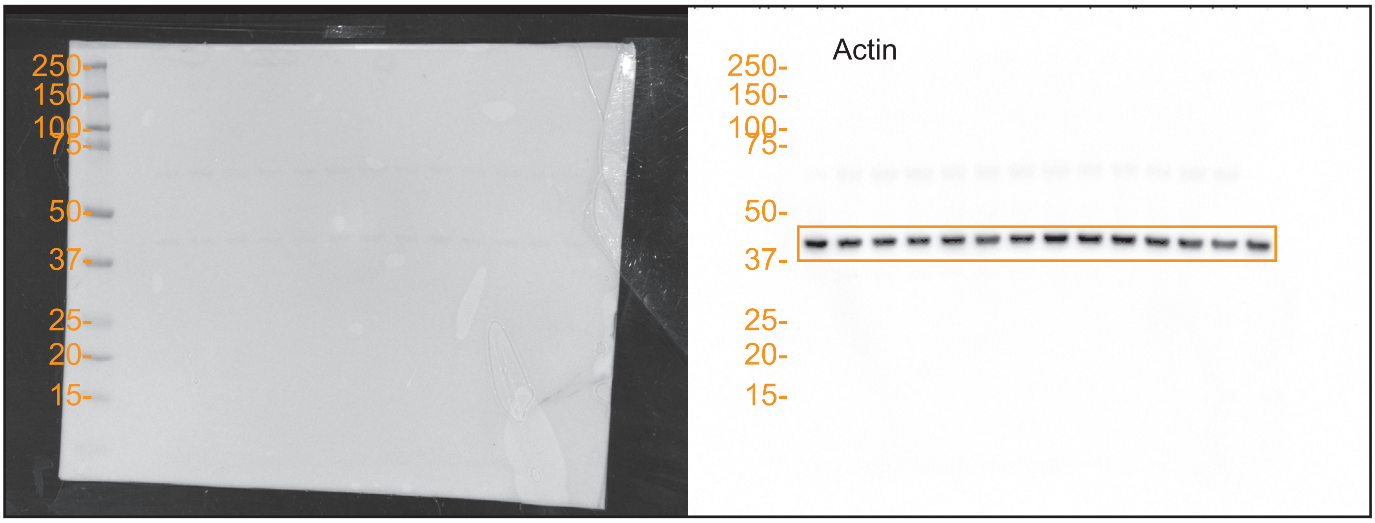

Fig. 5E

Figure as in manuscript:

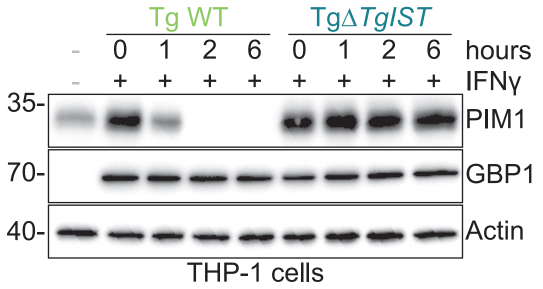

Uncropped gels with indicated size marker and crop area (lane annotation, if excluded from figure):

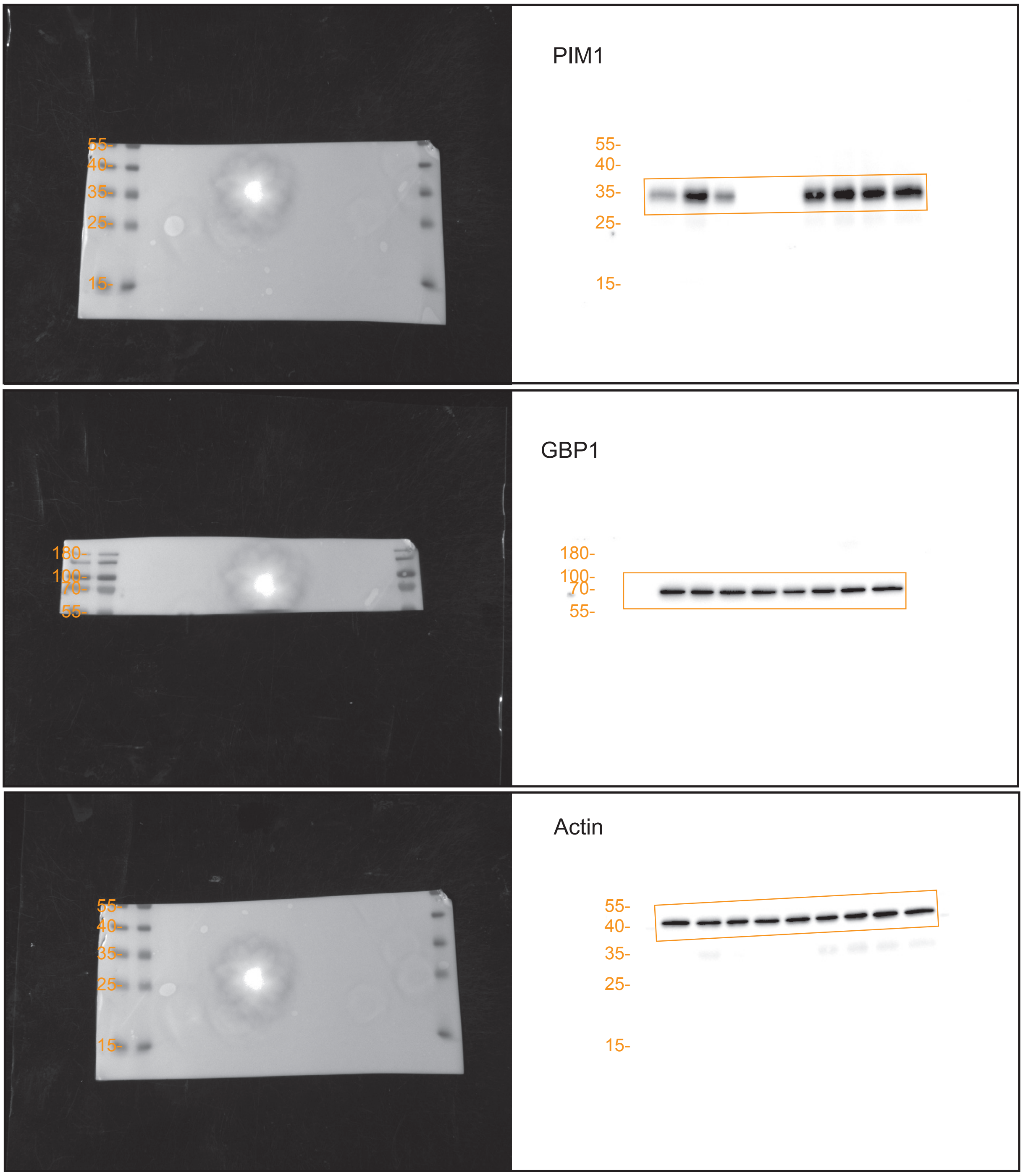

Fig. 5F

Figure as in manuscript:

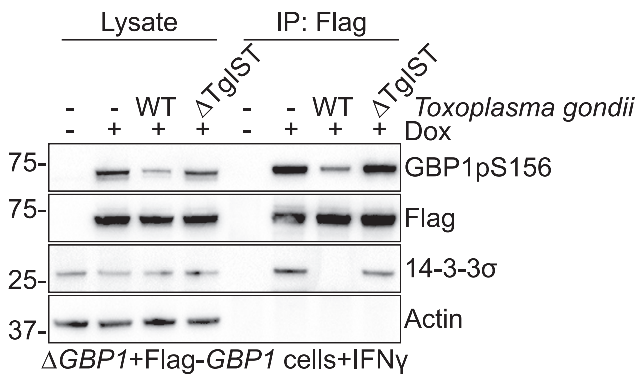

Uncropped gels with indicated size marker and crop area (lane annotation, if excluded from figure):

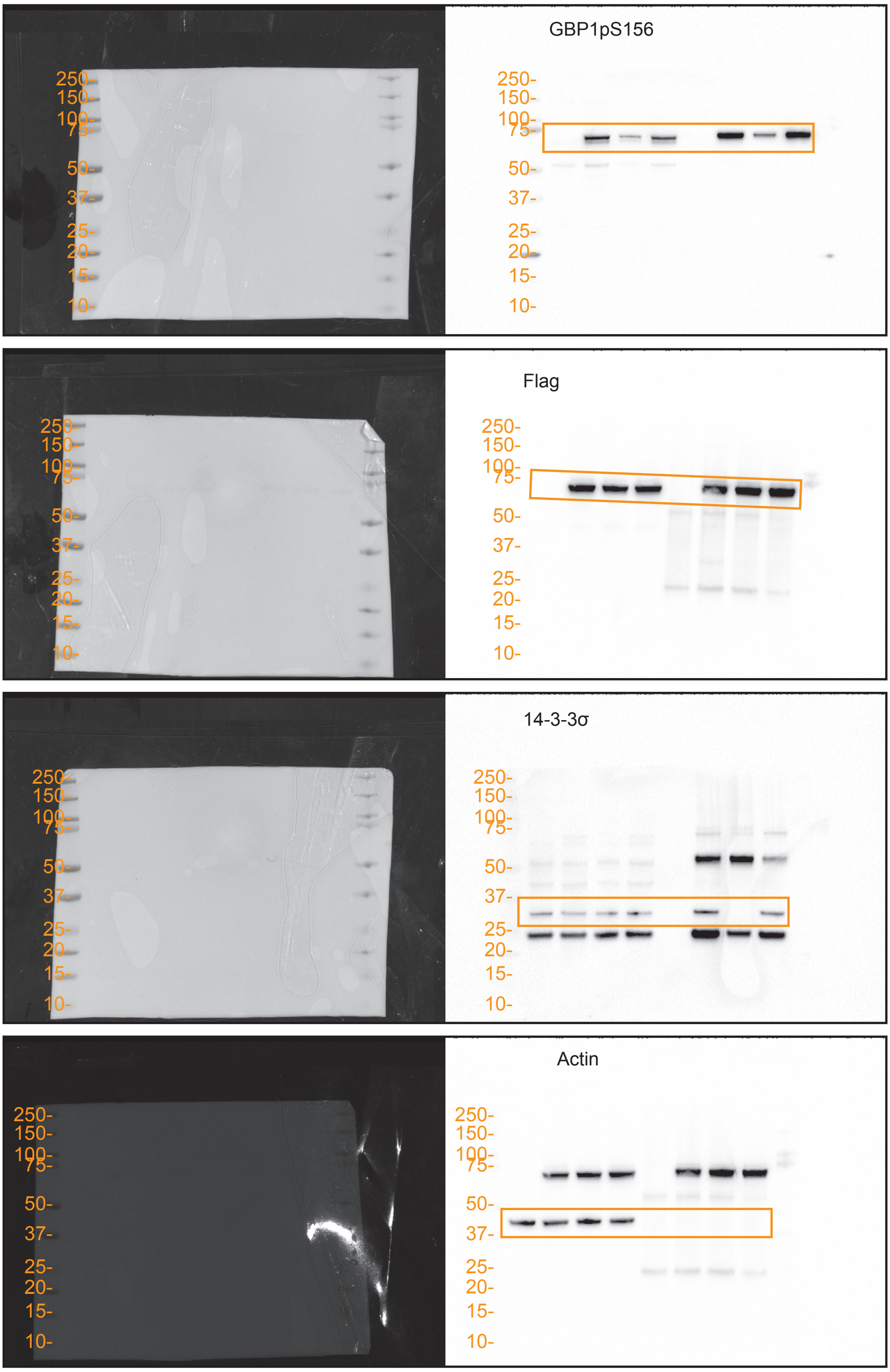

Fig. S1 - part1

Figure as in manuscript:

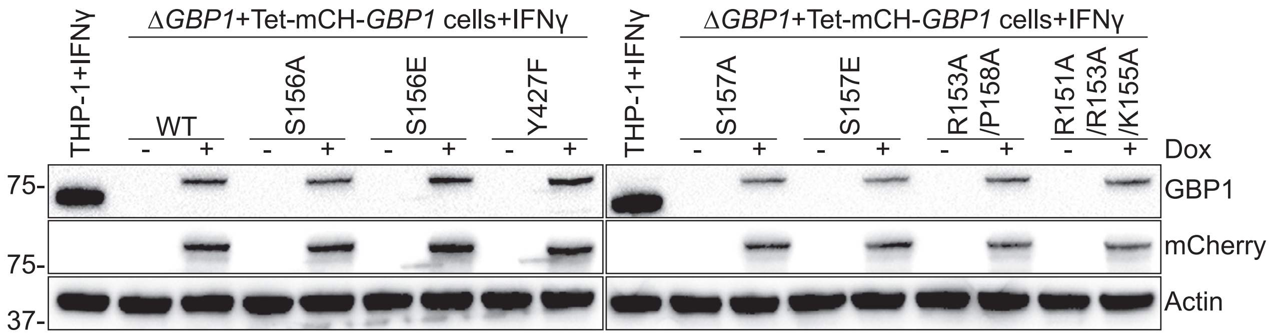

Uncropped gels with indicated size marker and crop area (lane annotation, if excluded from figure):

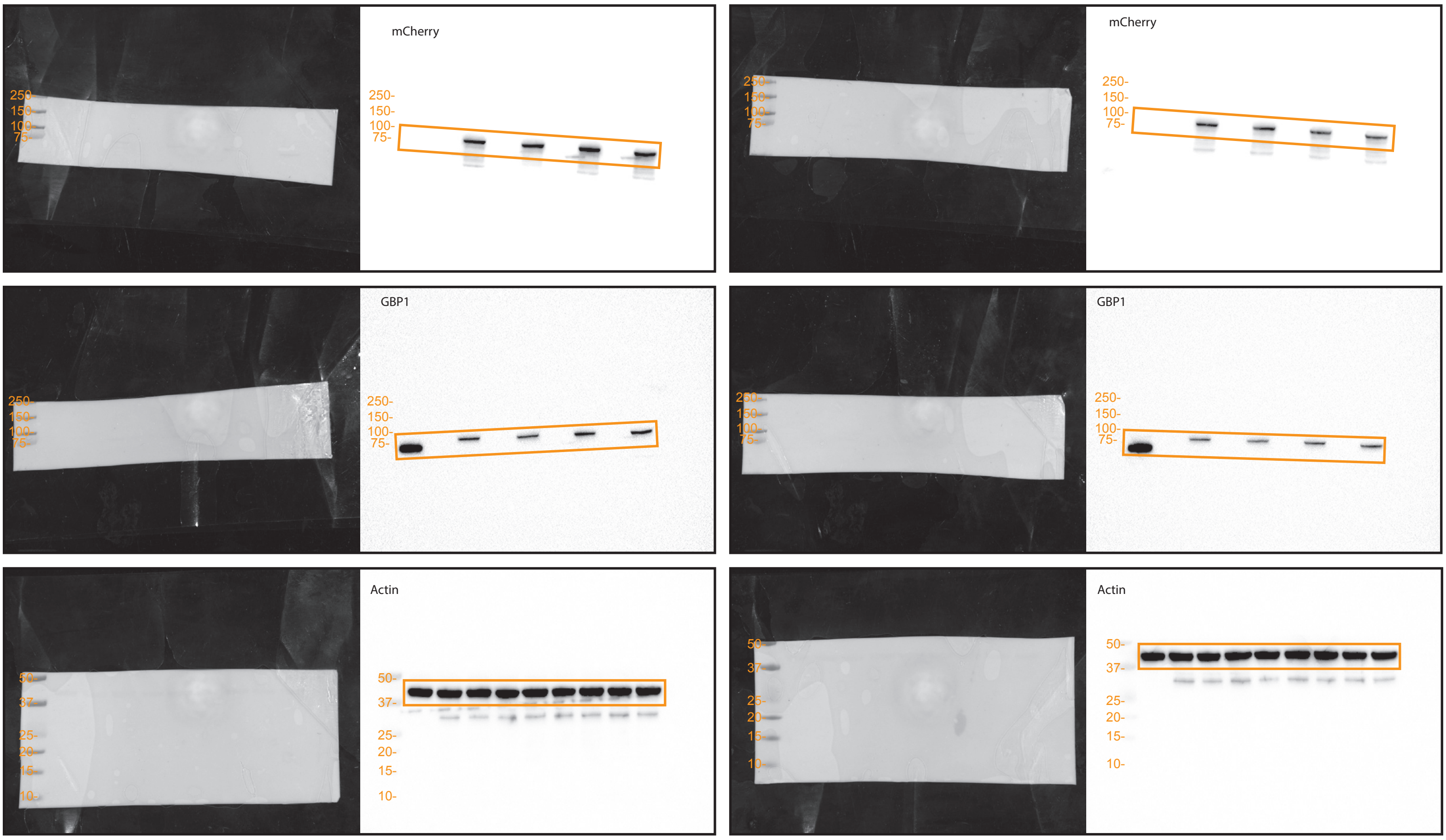

Fig. S1 - part 2

Figure as in manuscript:

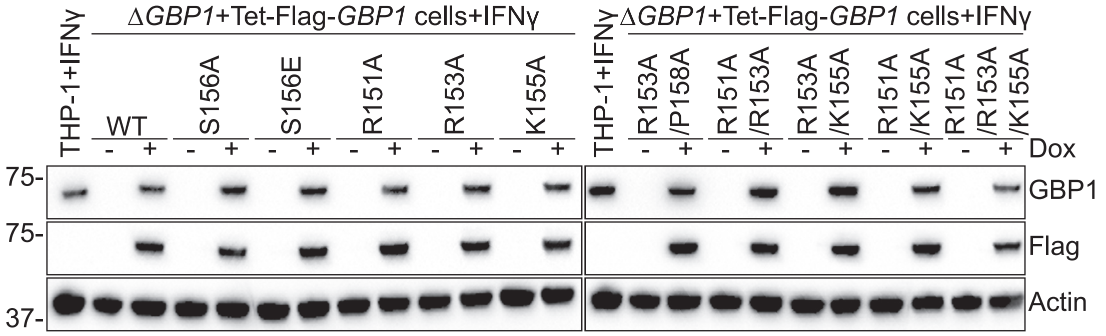

Uncropped gels with indicated size marker and crop area (lane annotation, if excluded from figure):

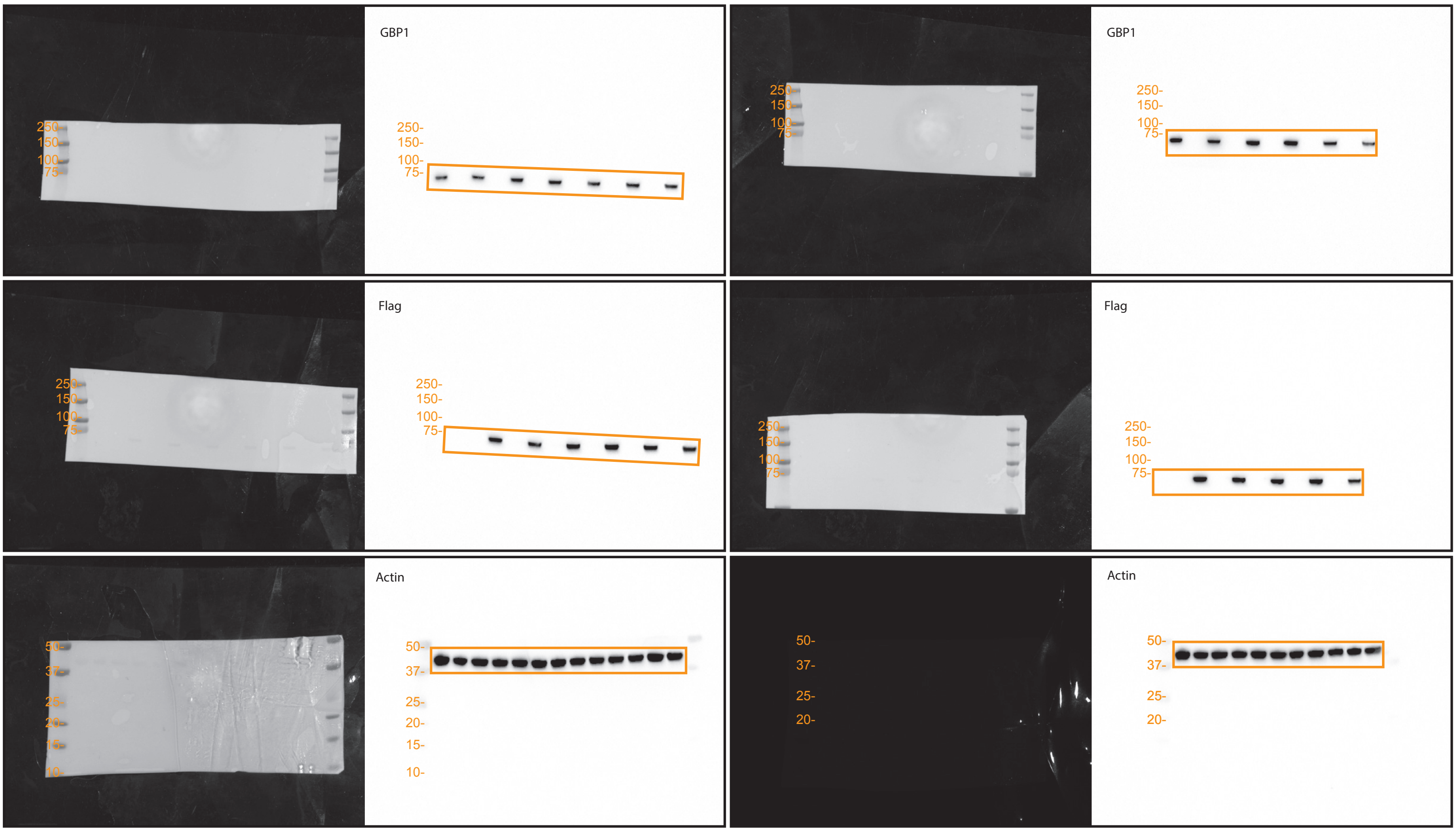

Fig. S3B

Figure as in manuscript:

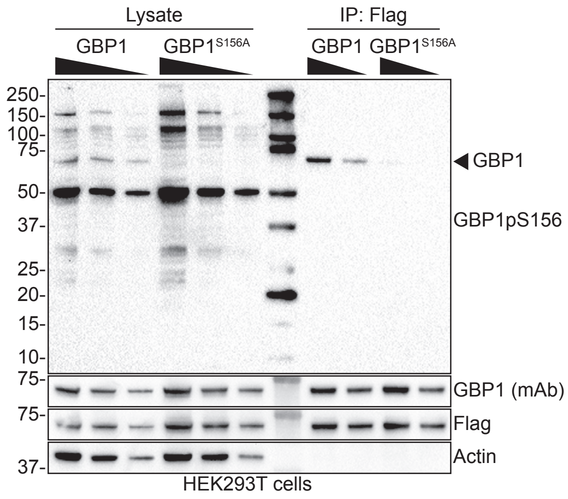

Uncropped gels with indicated size marker and crop area (lane annotation, if excluded from figure):

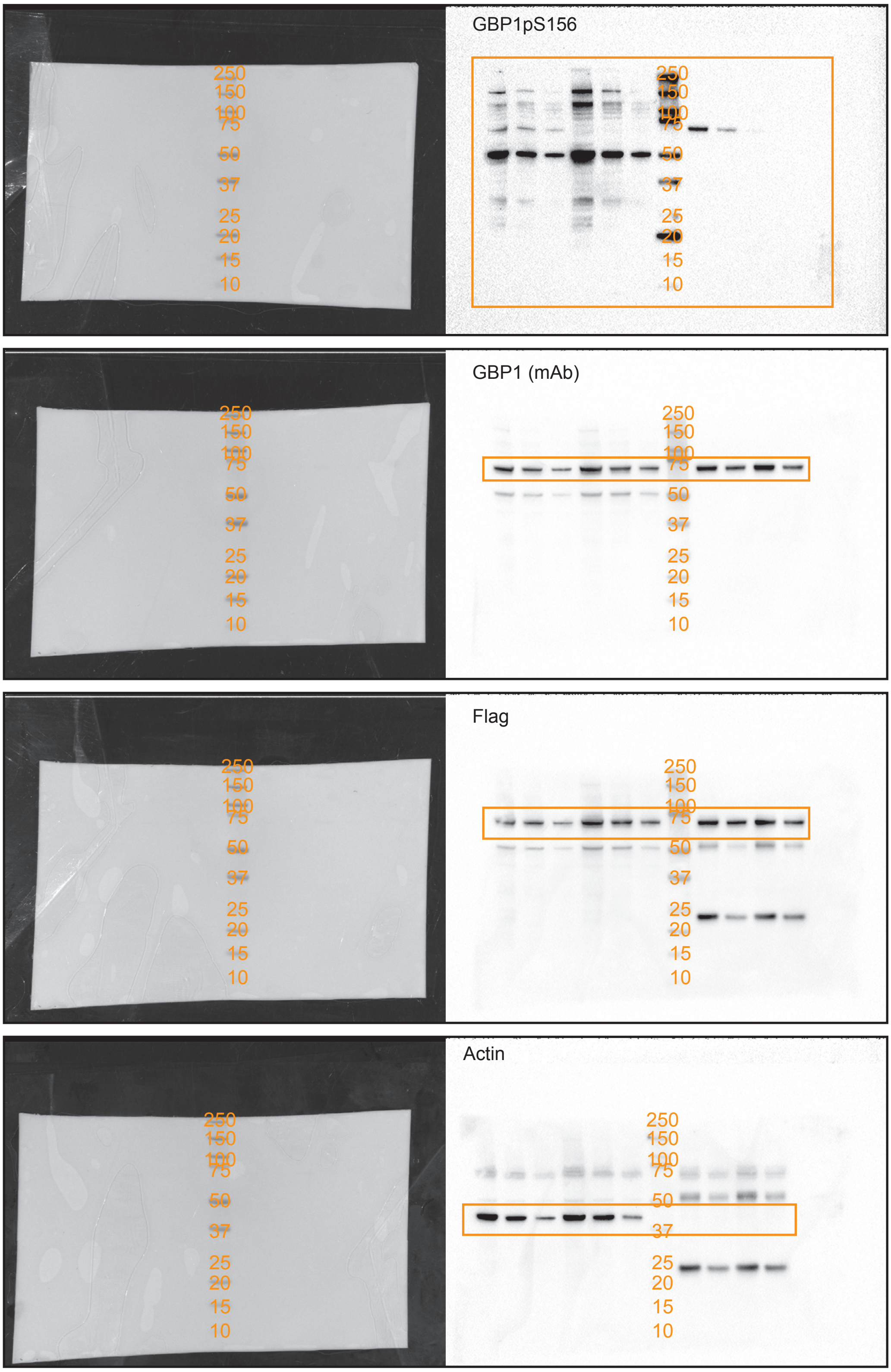

Fig. S3C

Figure as in manuscript:

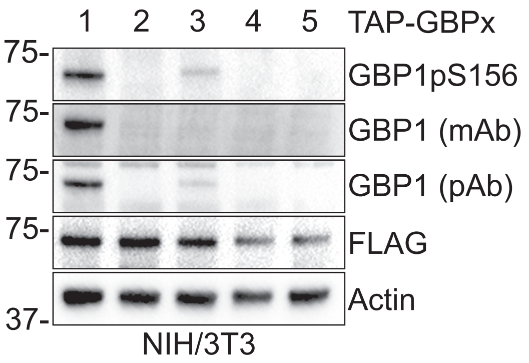

Uncropped gels with indicated size marker and crop area (lane annotation, if excluded from figure):

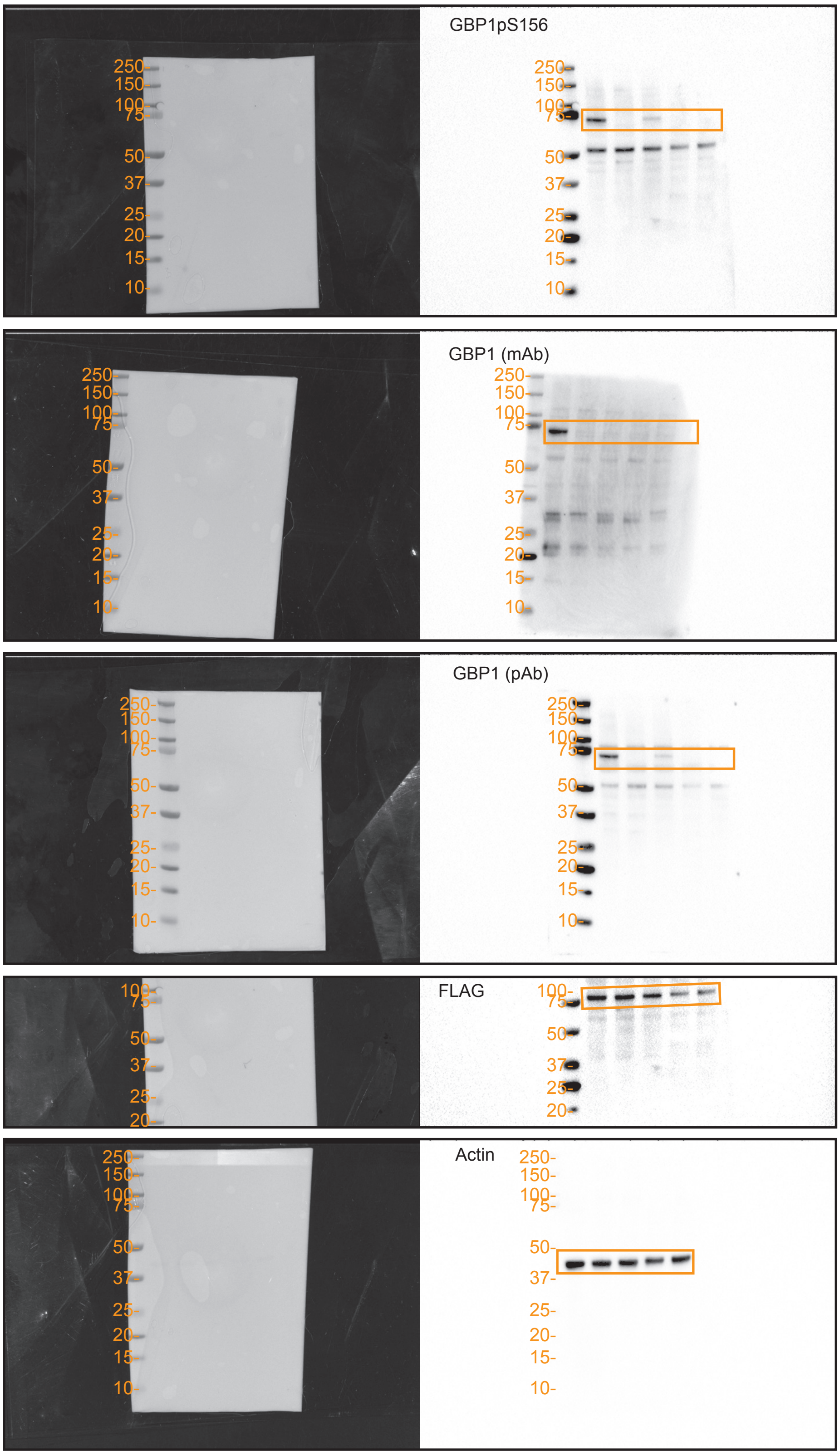

Fig. S4B - part 1

Figure as in manuscript:

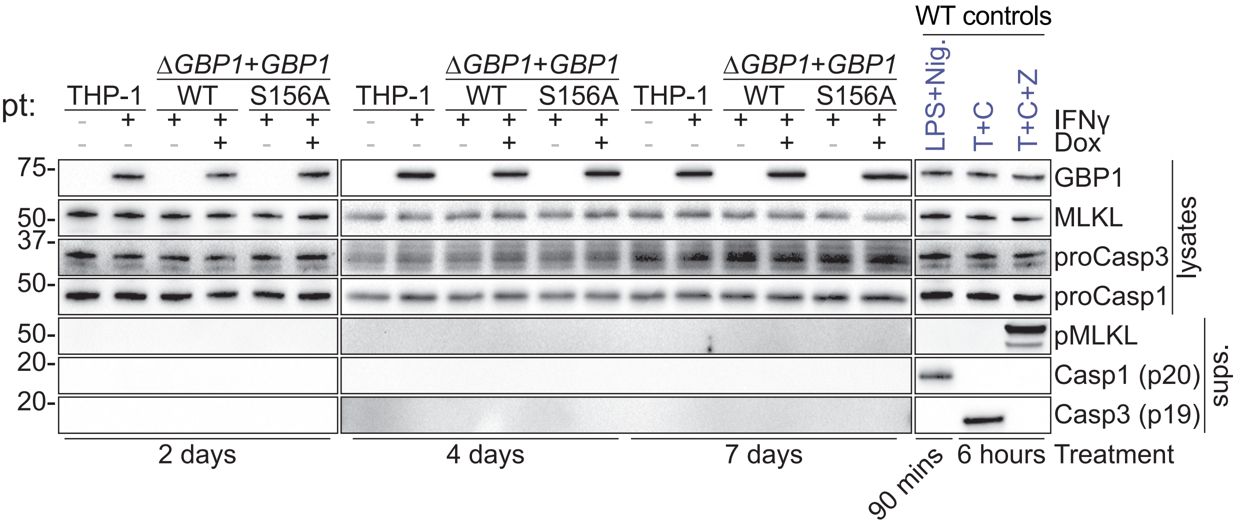

Uncropped gels with indicated size marker and crop area (lane annotation, if excluded from figure):

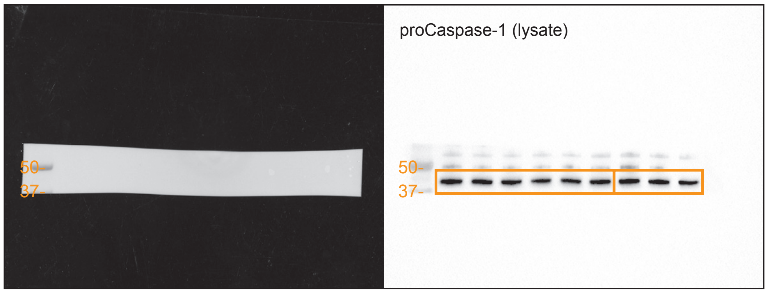

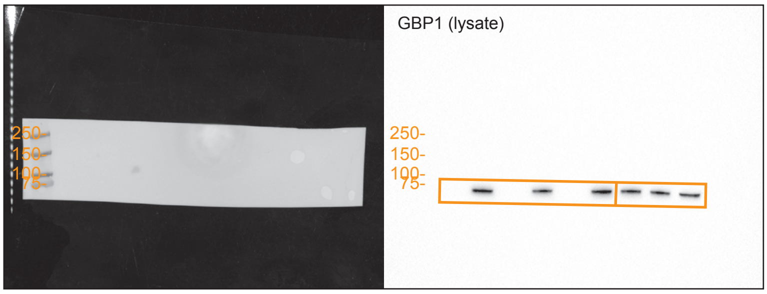

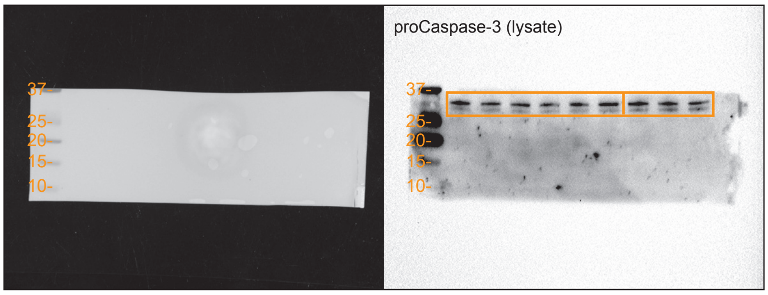

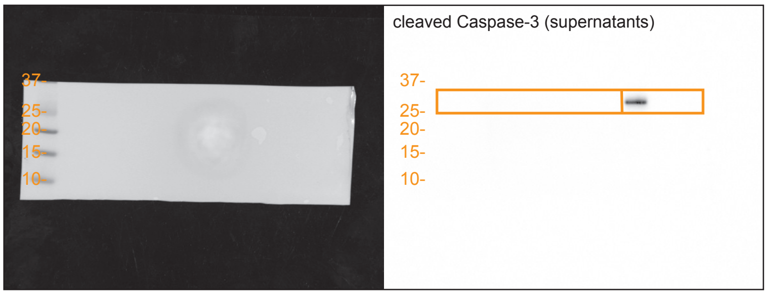

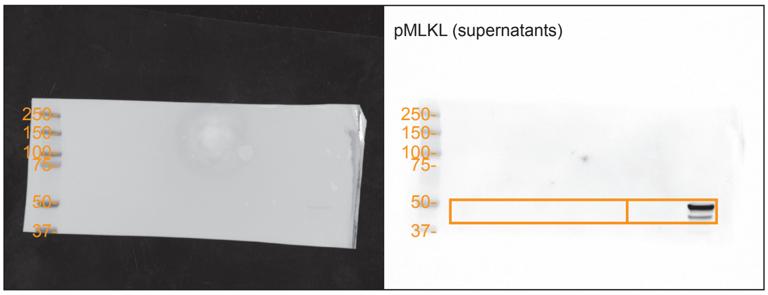

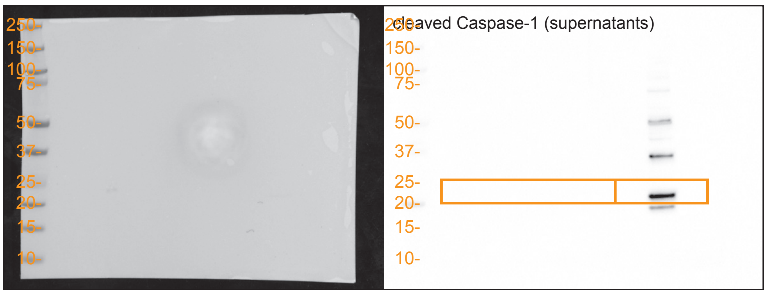

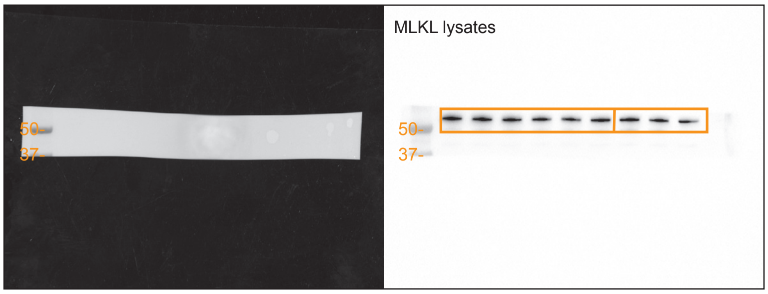

Fig. S4B - part2

Figure as in manuscript:

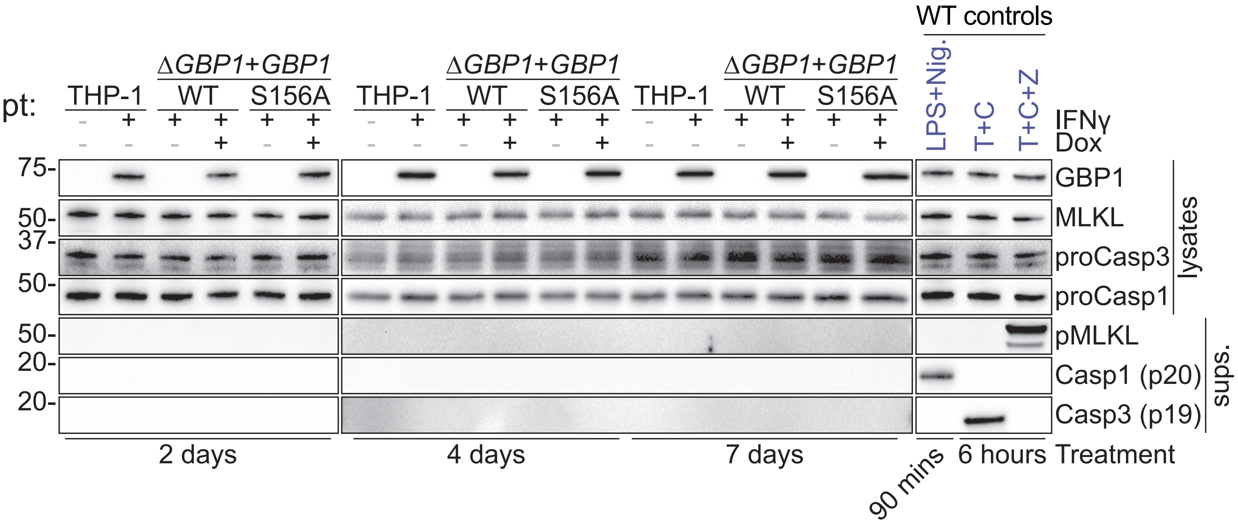

Uncropped gels with indicated size marker and crop area (lane annotation, if excluded from figure):

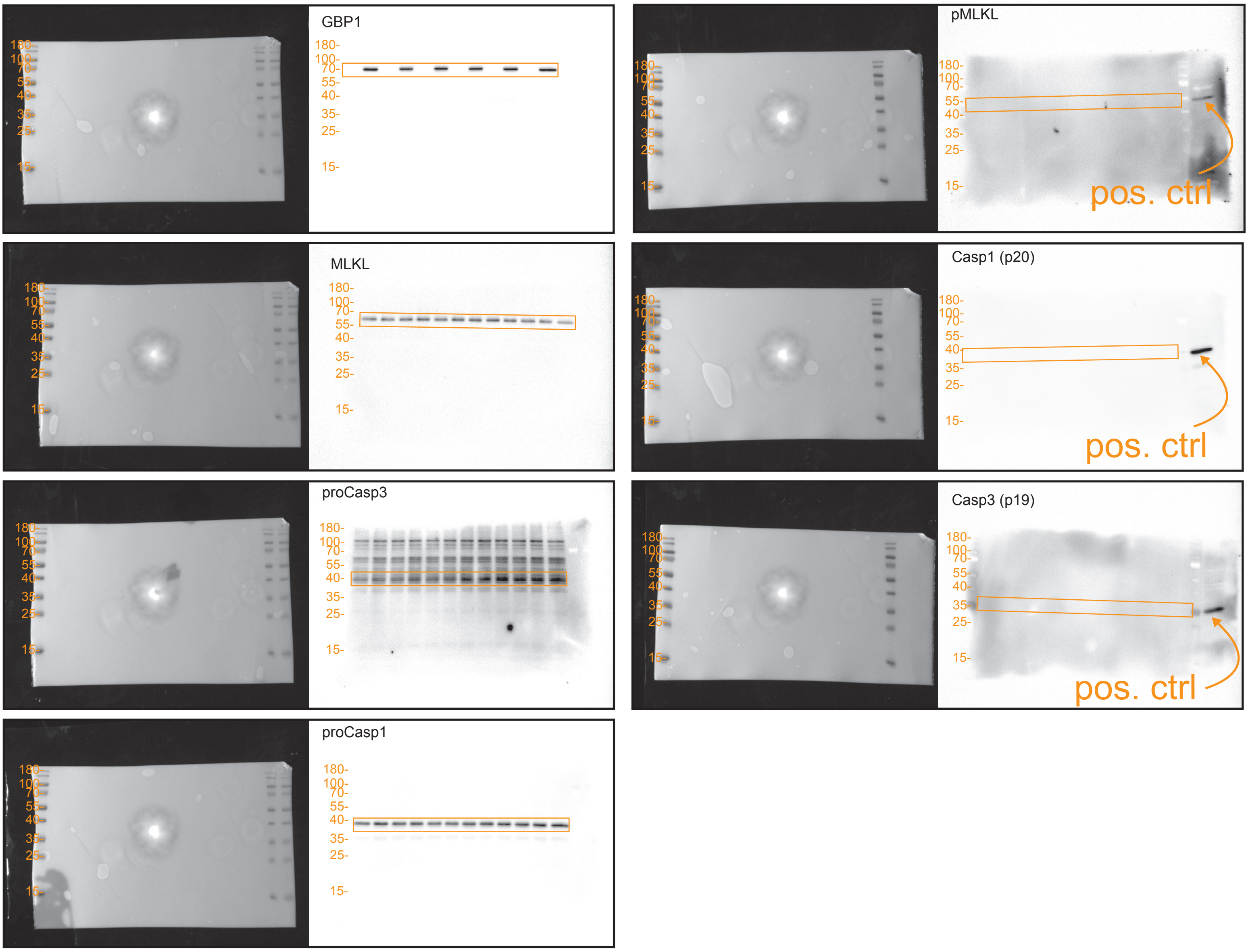

Fig. S6B

Figure as in manuscript:

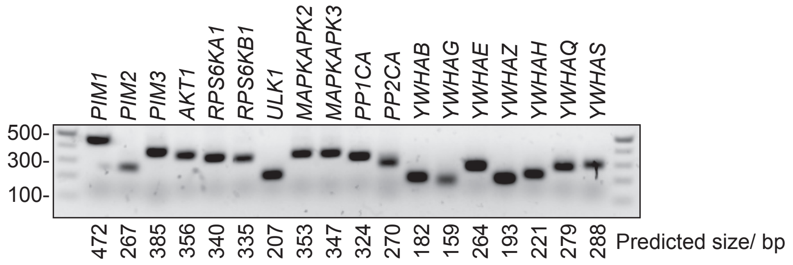

Uncropped gels with indicated size marker and crop area (lane annotation, if excluded from figure):

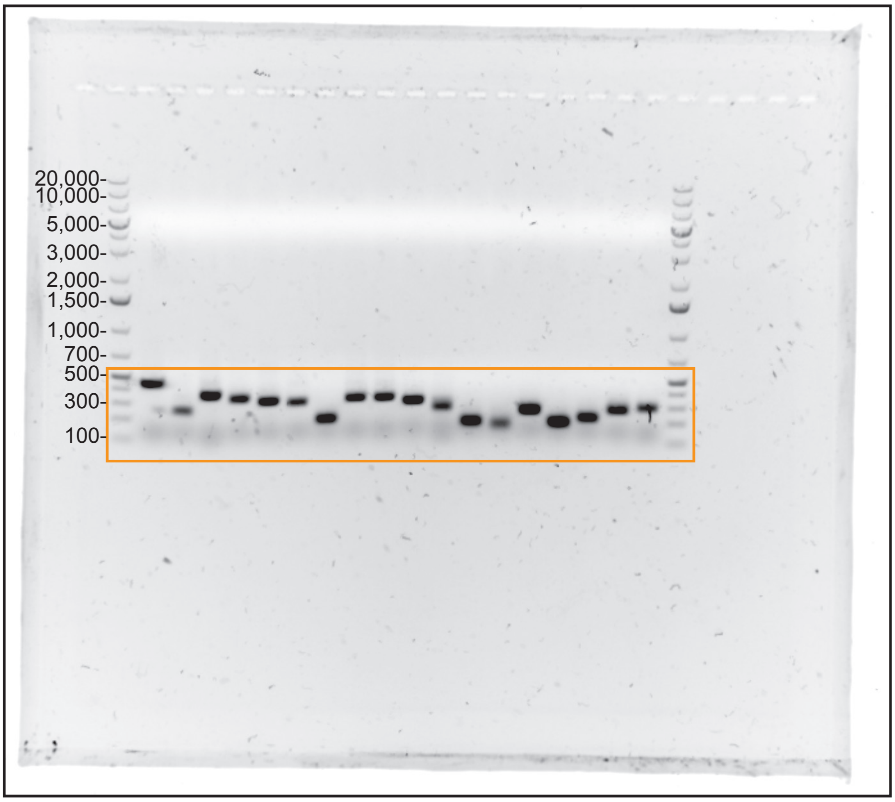

Fig. S7A - part 1

Figure as in manuscript:

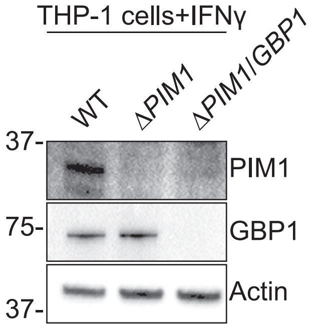

Uncropped gels with indicated size marker and crop area (lane annotation, if excluded from figure):

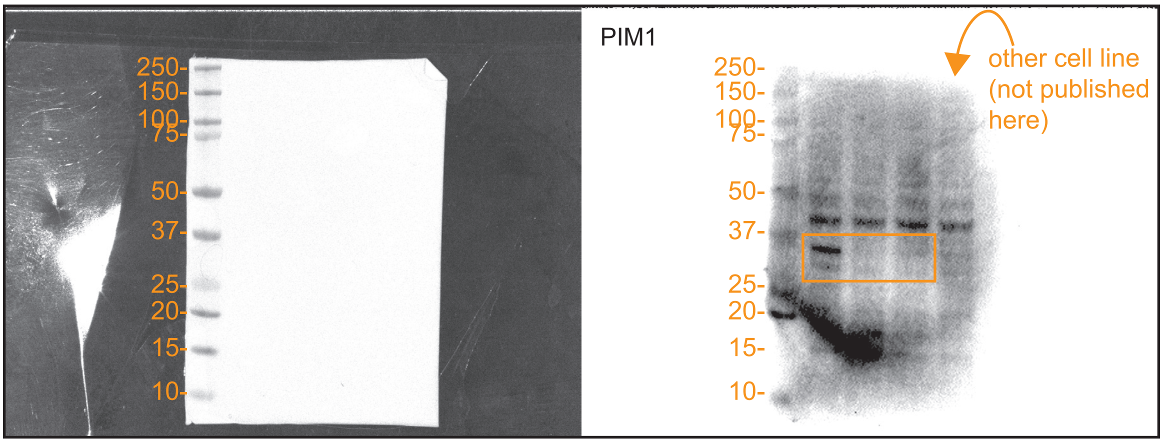

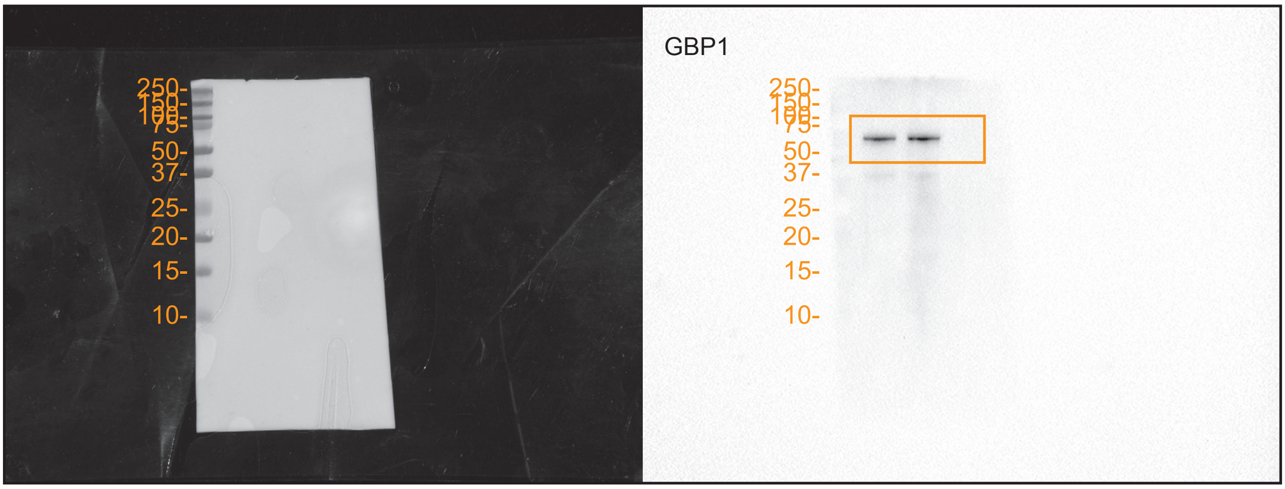

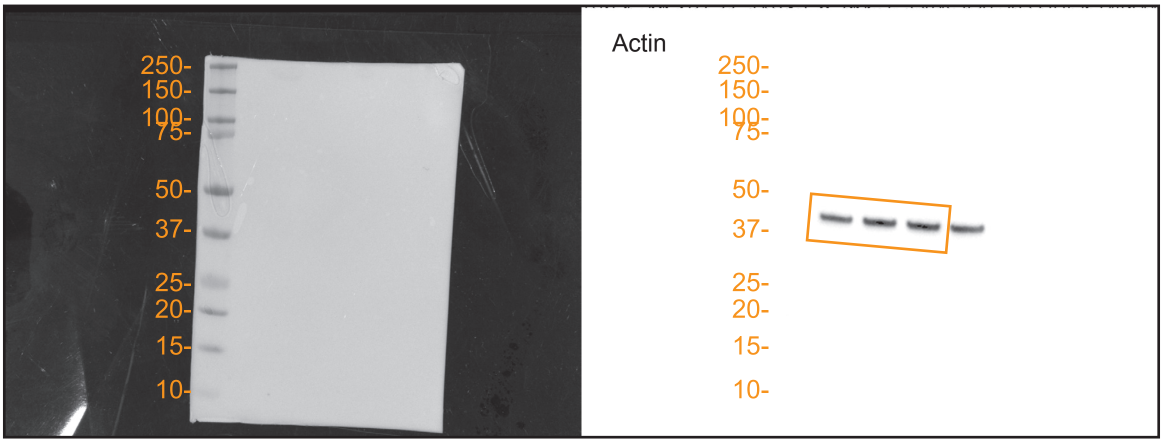

Fig. S7A - part 2

Figure as in manuscript:

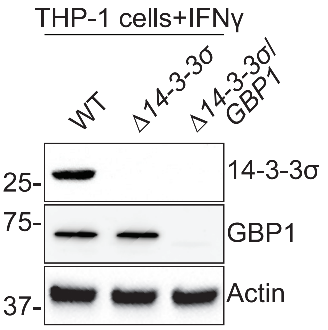

Uncropped gels with indicated size marker and crop area (lane annotation, if excluded from figure):

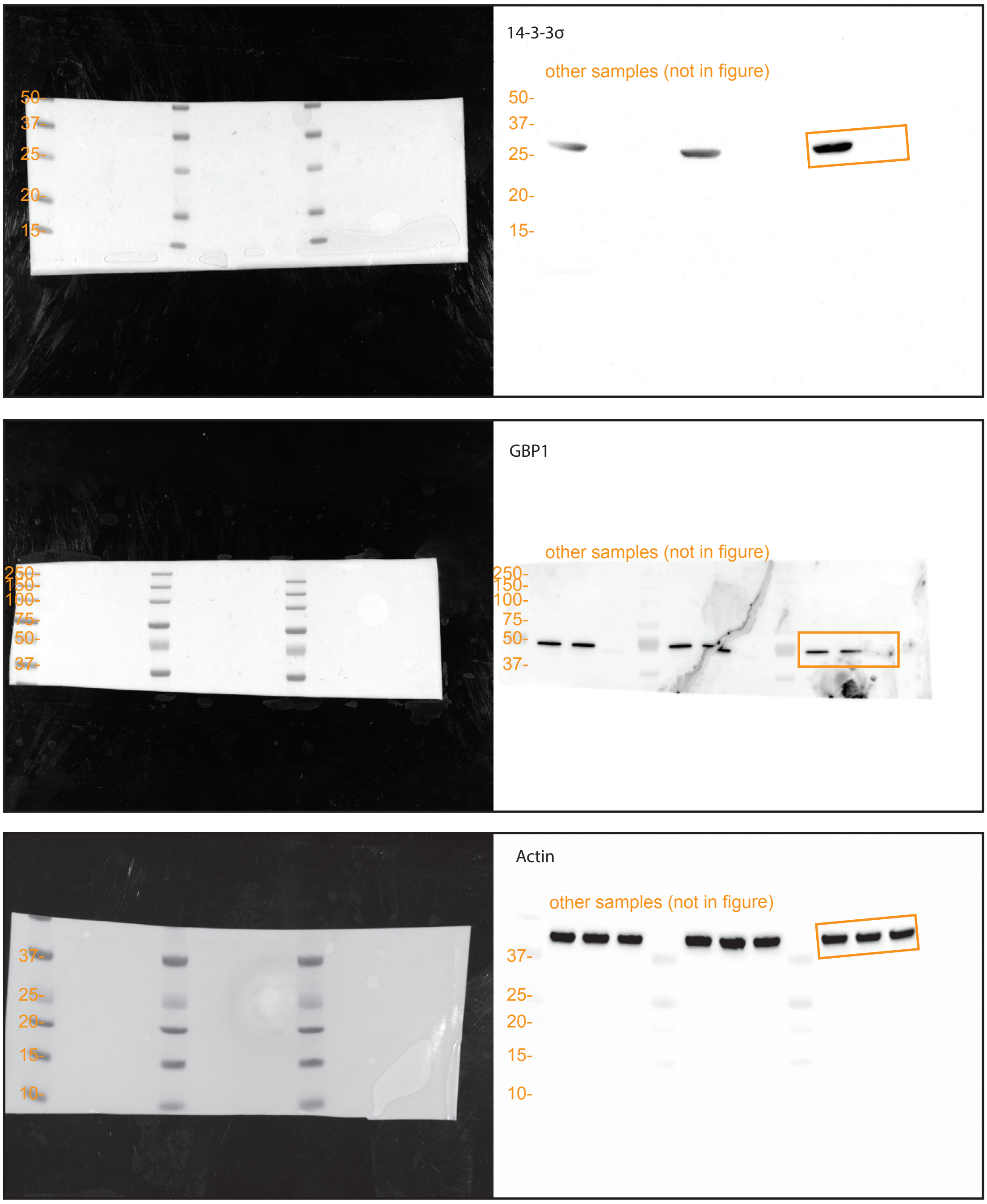

Fig. S7B

Figure as in manuscript:

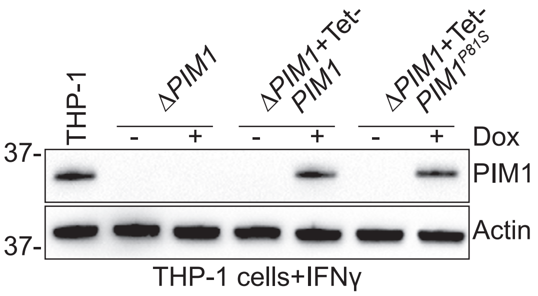

Uncropped gels with indicated size marker and crop area (lane annotation, if excluded from figure):

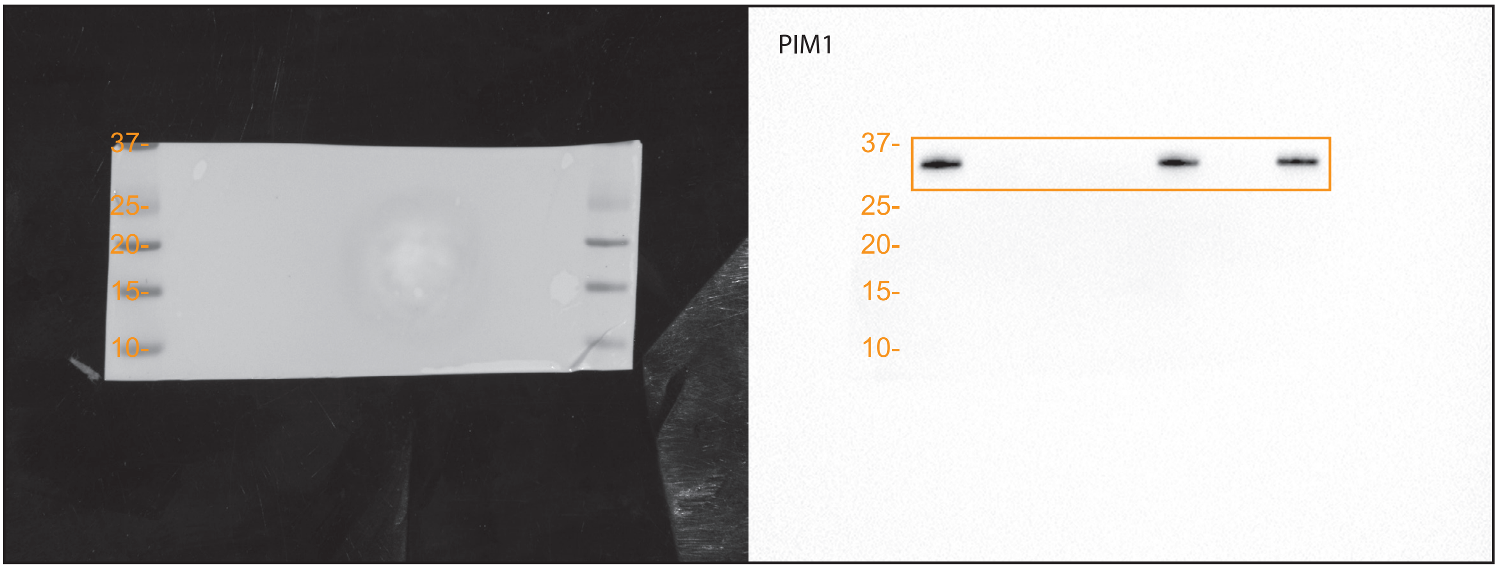

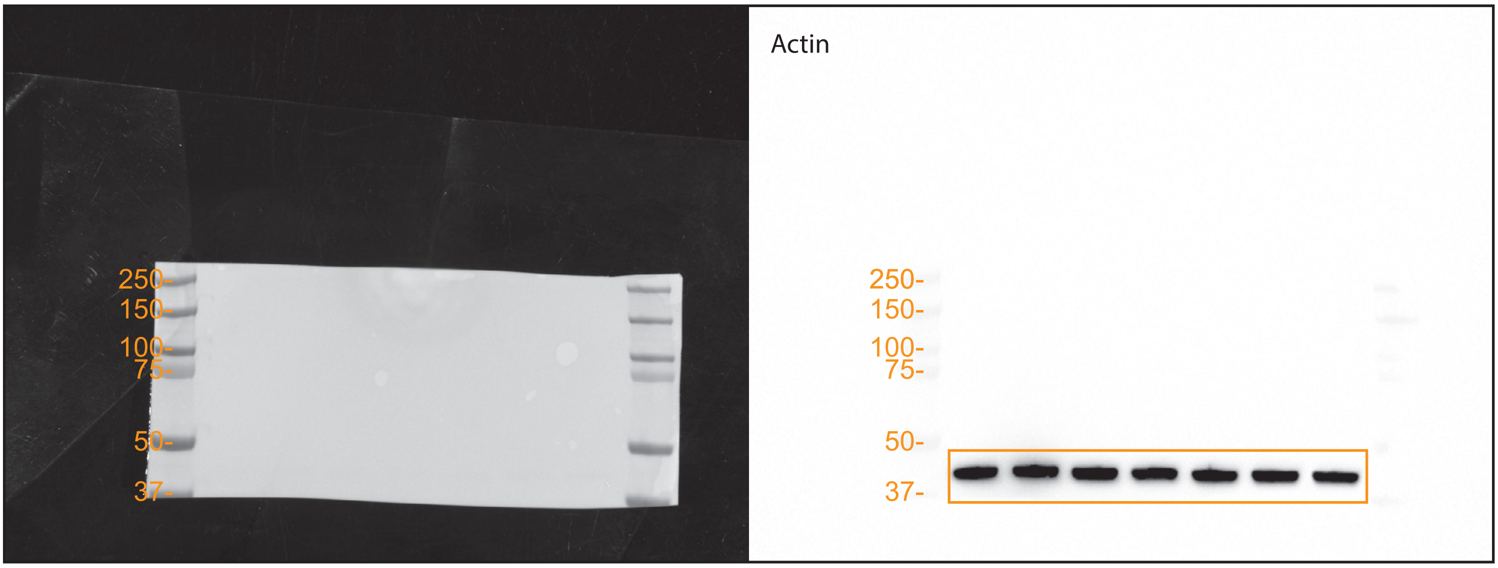

Fig. S7C

Figure as in manuscript:

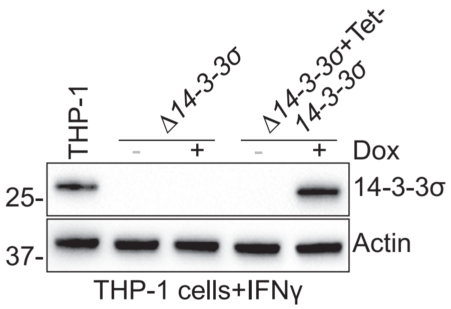

Uncropped gels with indicated size marker and crop area (lane annotation, if excluded from figure):

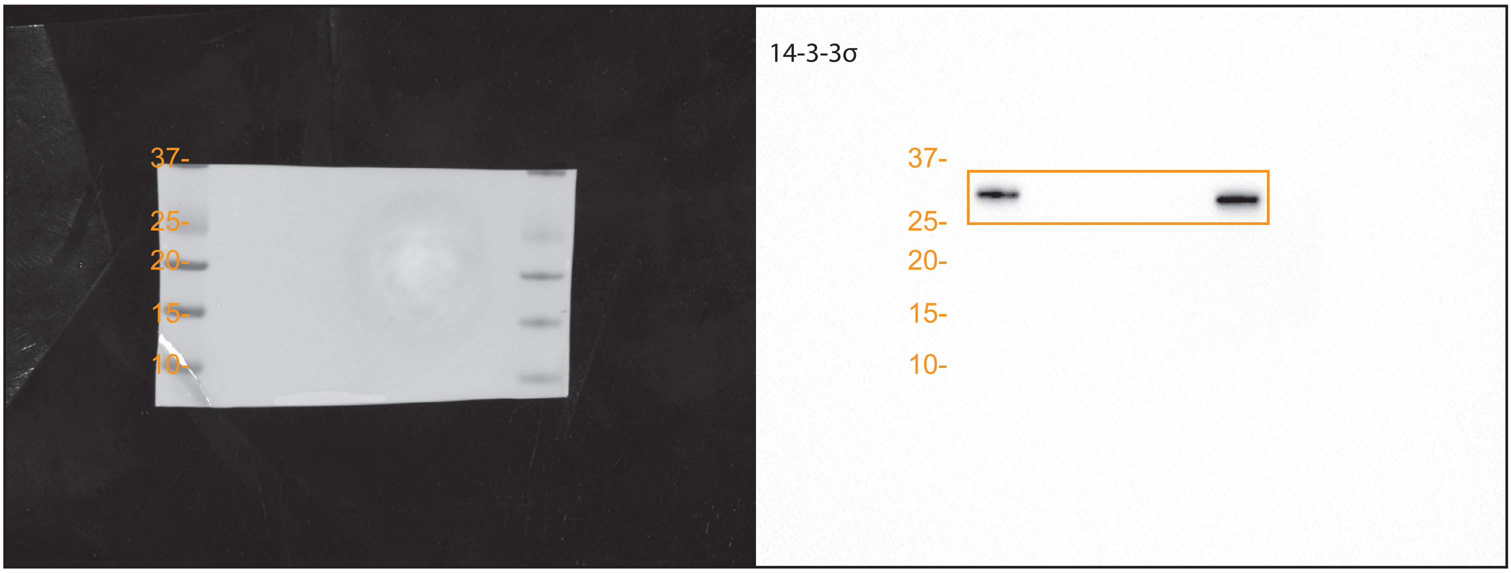

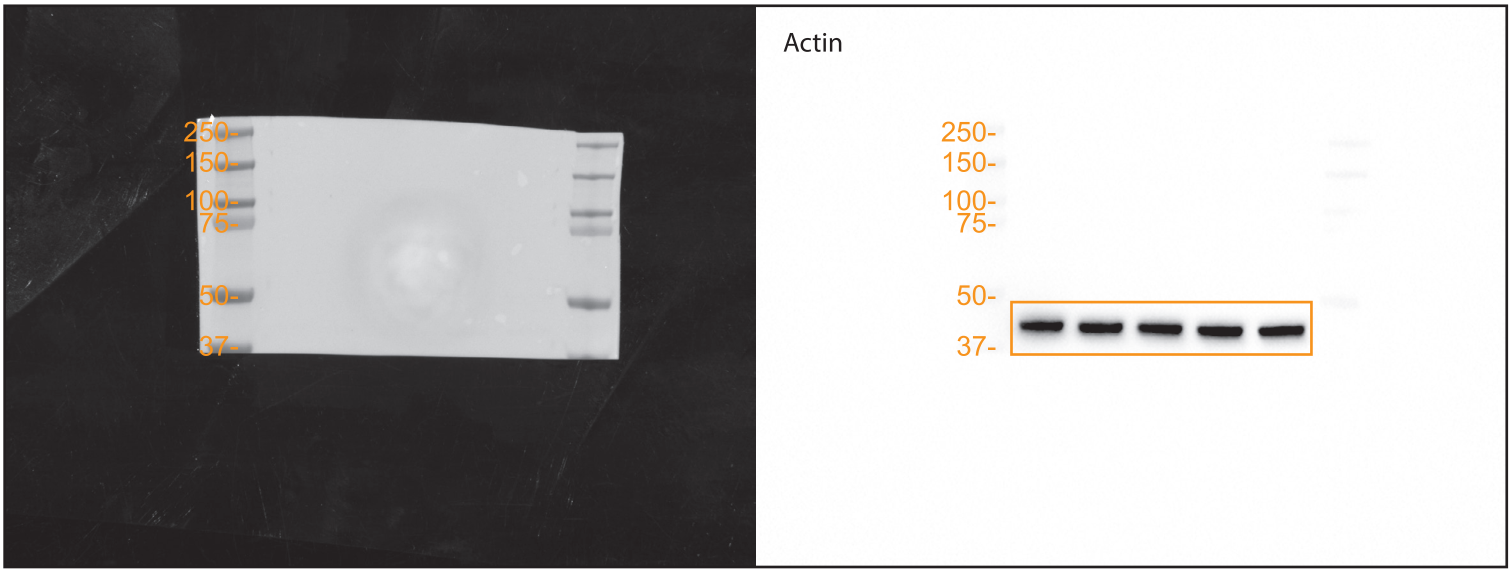

Fig. S8A

Figure as in manuscript:

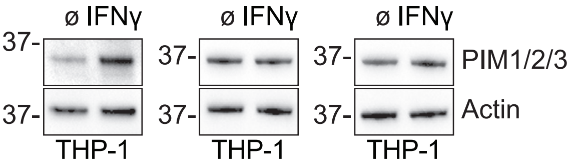

Uncropped gels with indicated size marker and crop area (lane annotation, if excluded from figure):

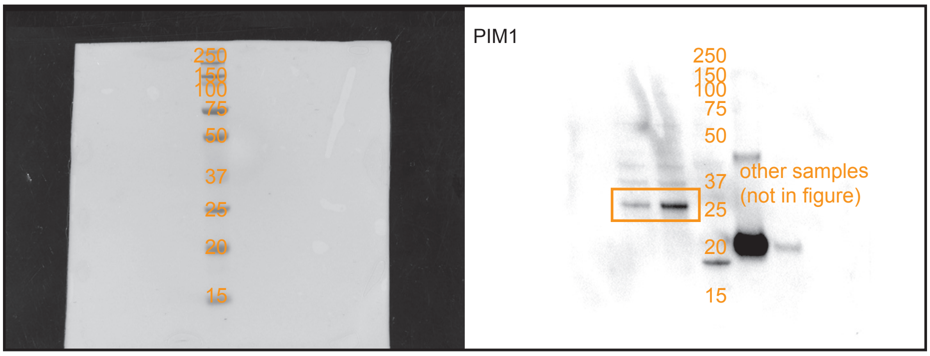

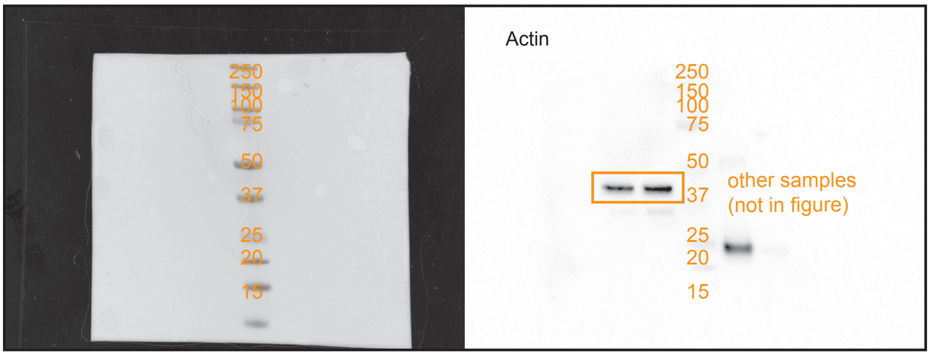

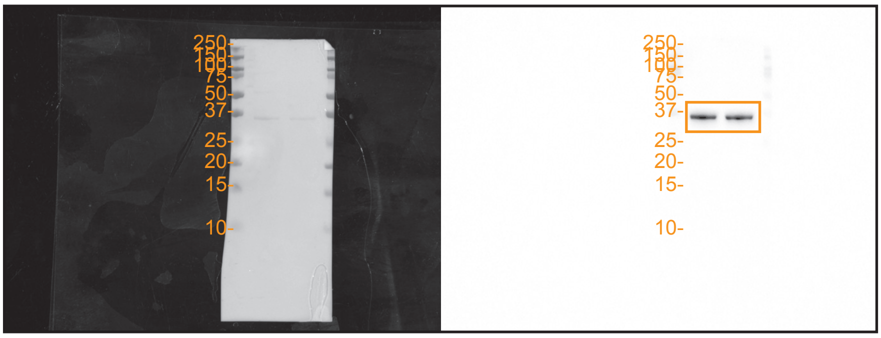

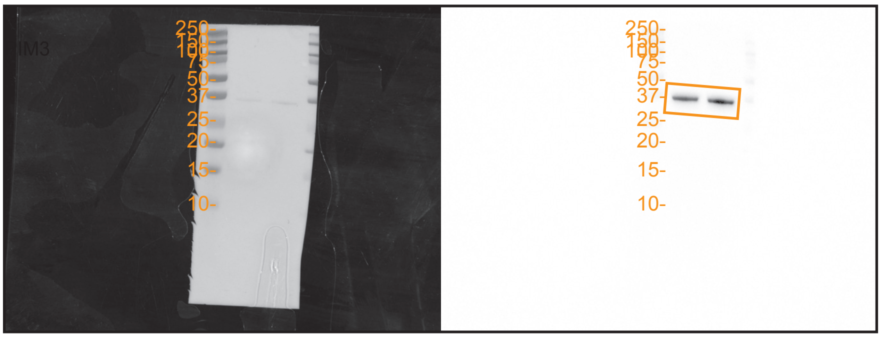

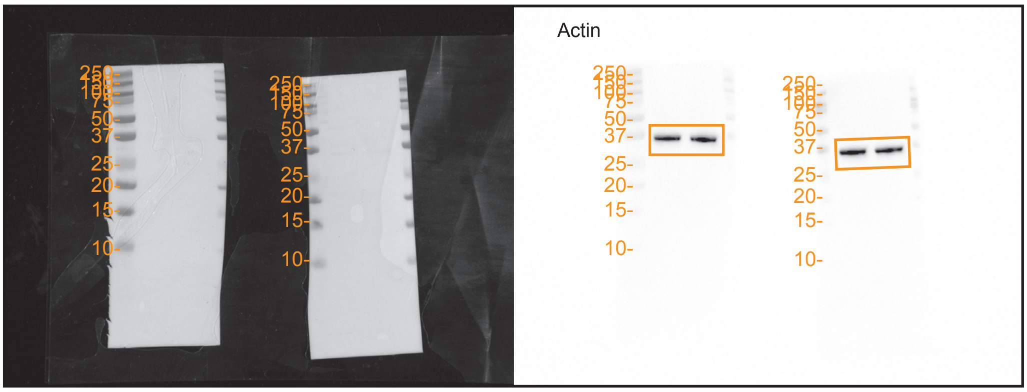

Fig. S8C

Figure as in manuscript:

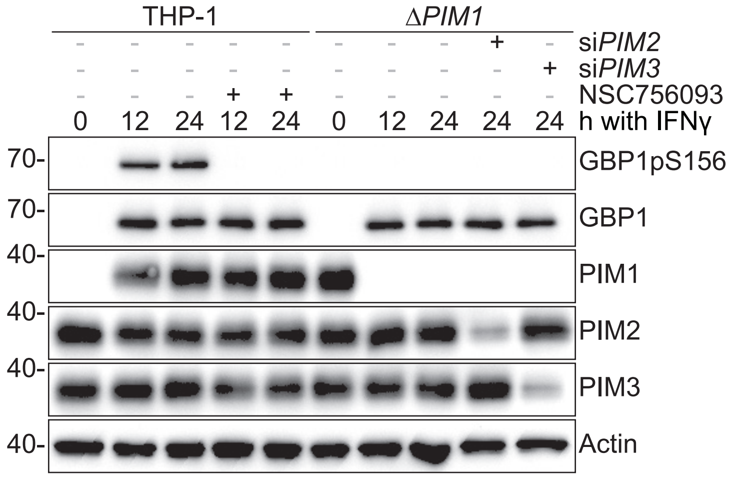

Uncropped gels with indicated size marker and crop area (lane annotation, if excluded from figure):

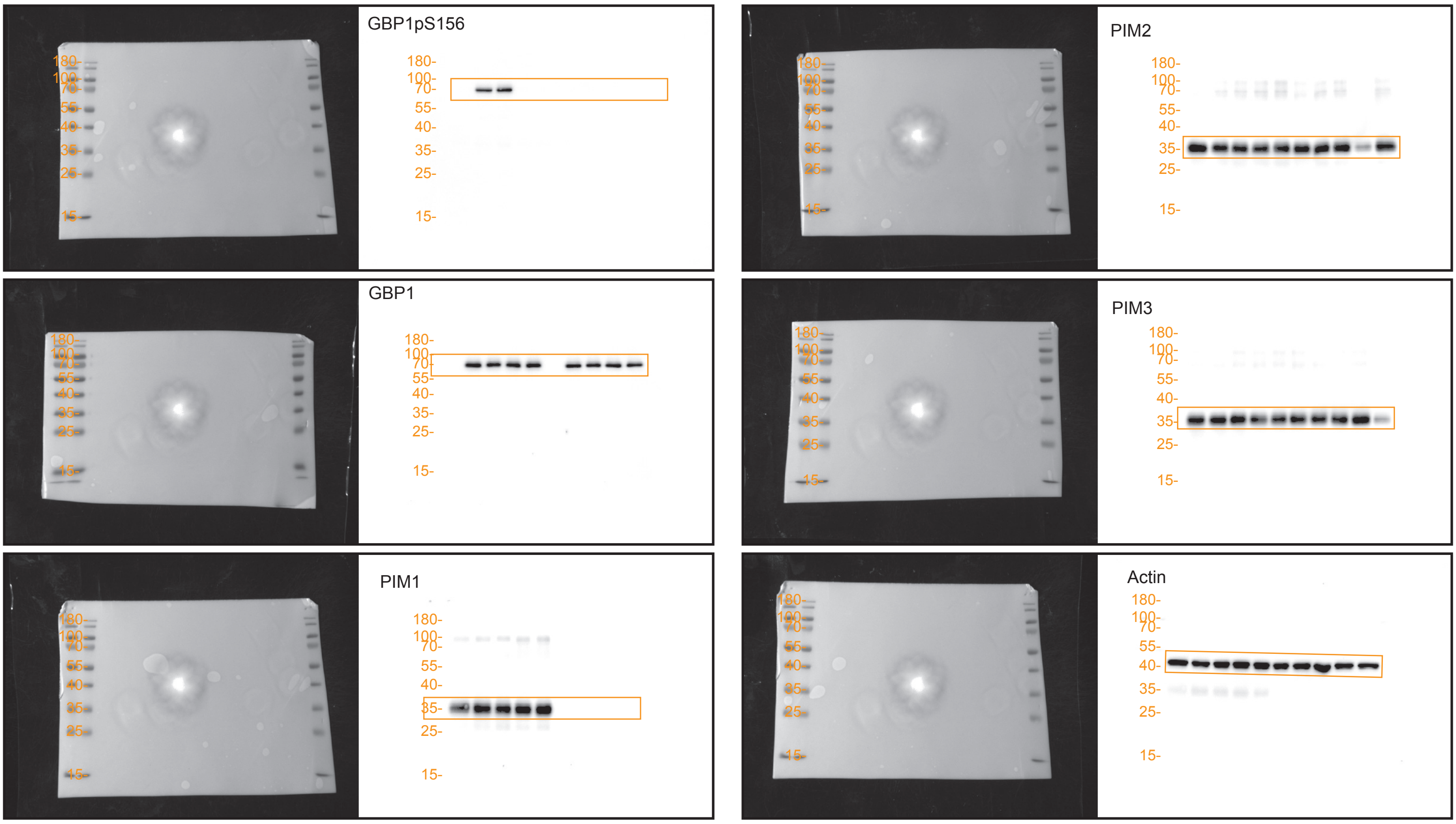

Fig. S8D

Figure as in manuscript:

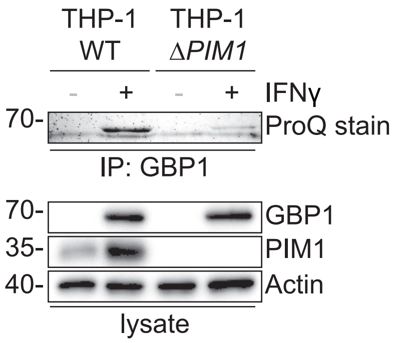

Uncropped gels with indicated size marker and crop area (lane annotation, if excluded from figure):

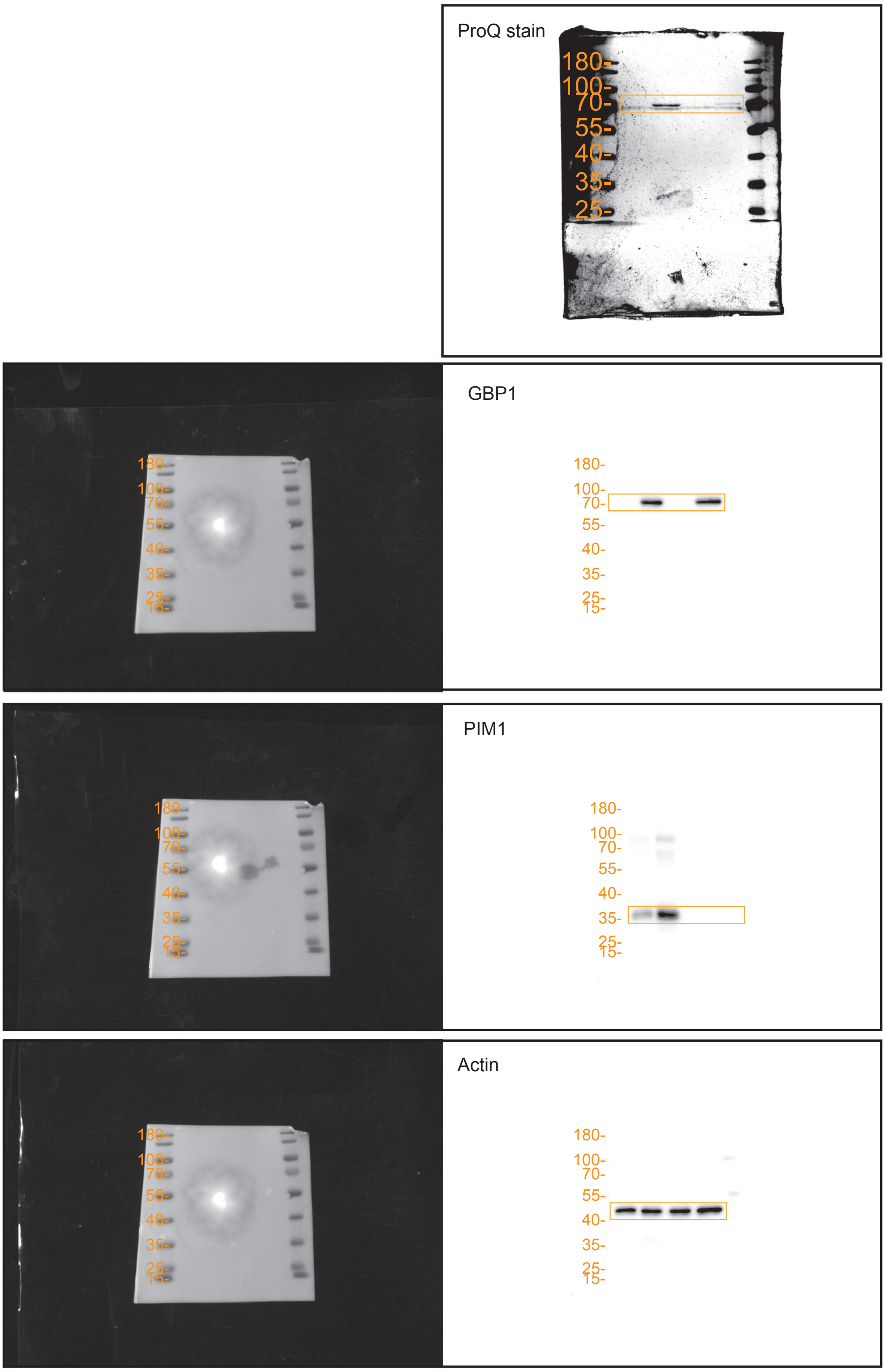

Fig. S8E

Figure as in manuscript:

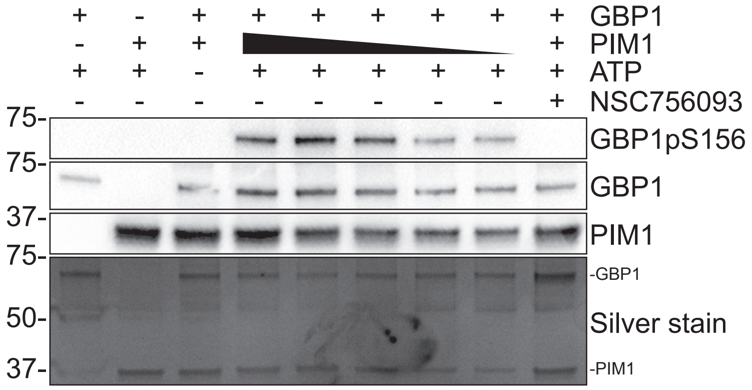

Uncropped gels with indicated size marker and crop area (lane annotation, if excluded from figure):

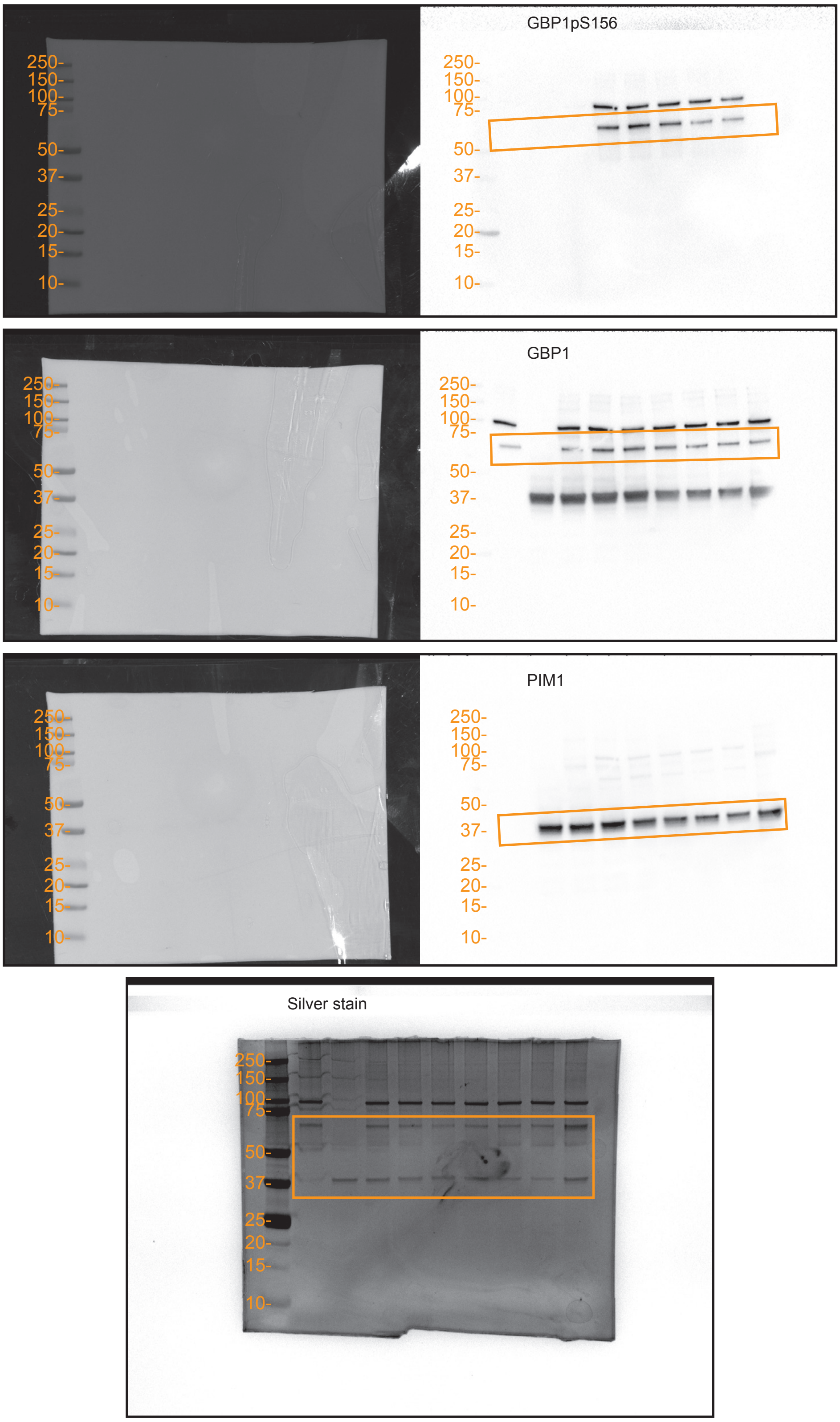

Fig. S9B

Figure as in manuscript:

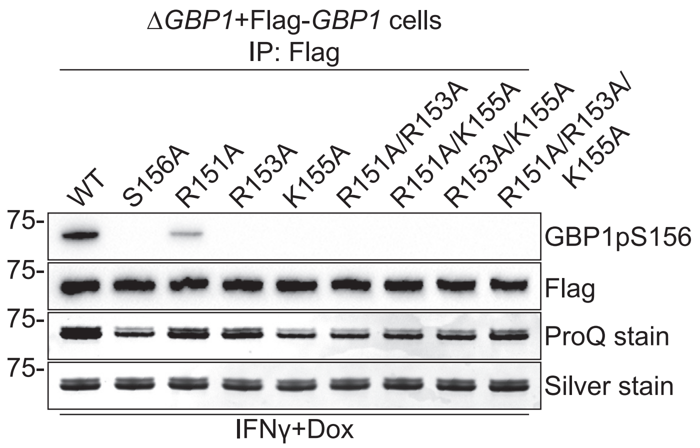

Uncropped gels with indicated size marker and crop area (lane annotation, if excluded from figure):

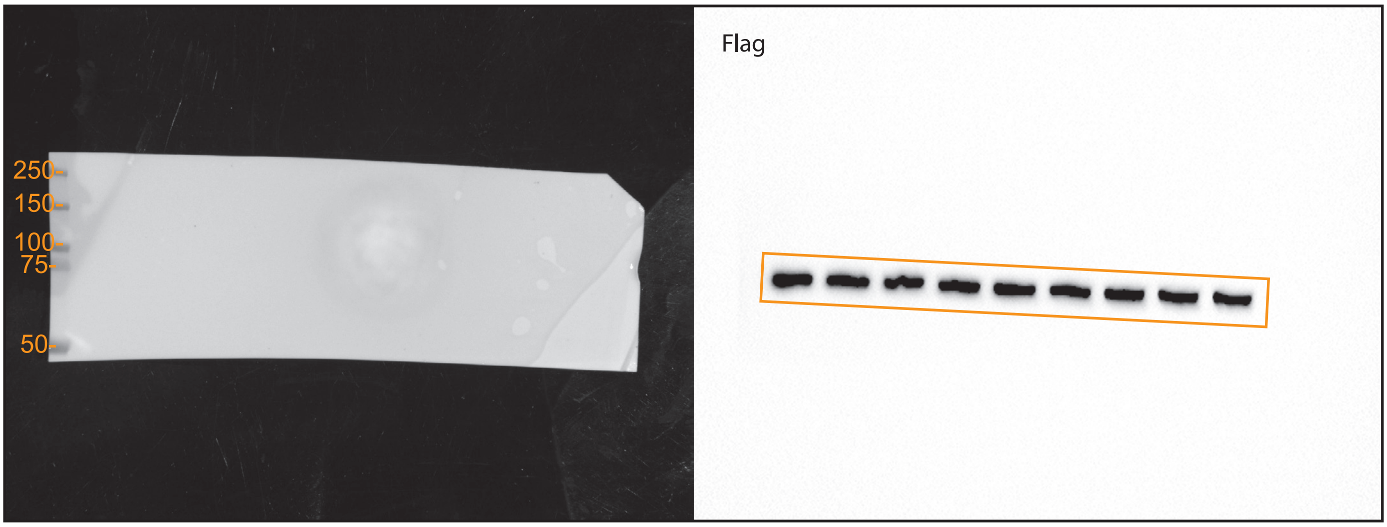

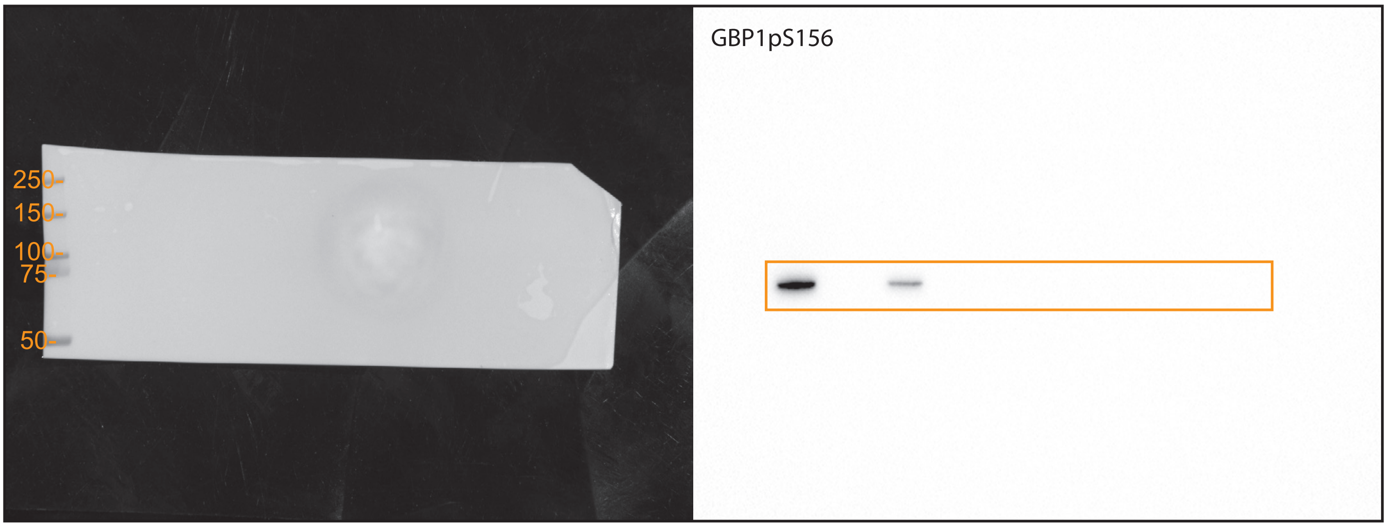

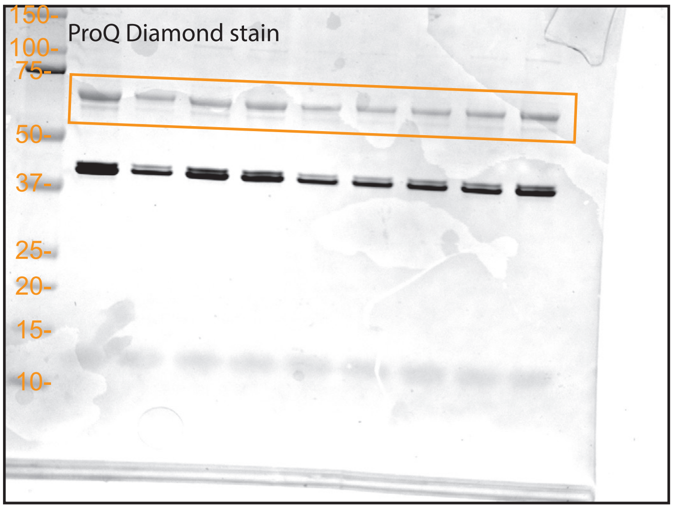

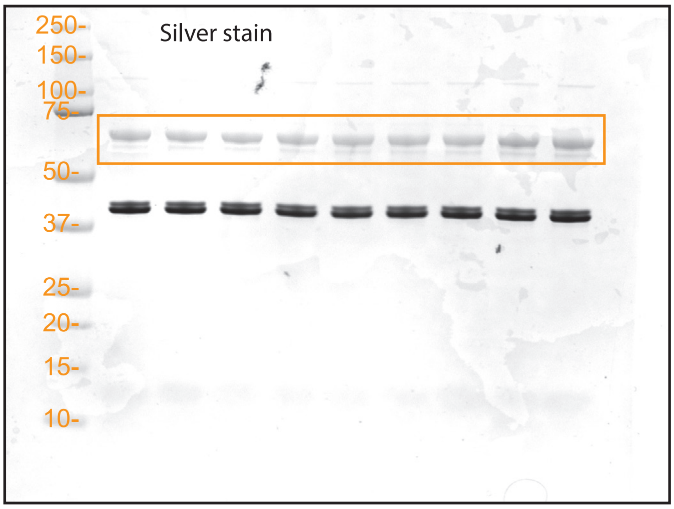

Fig. S10B - part 1

Figure as in manuscript:

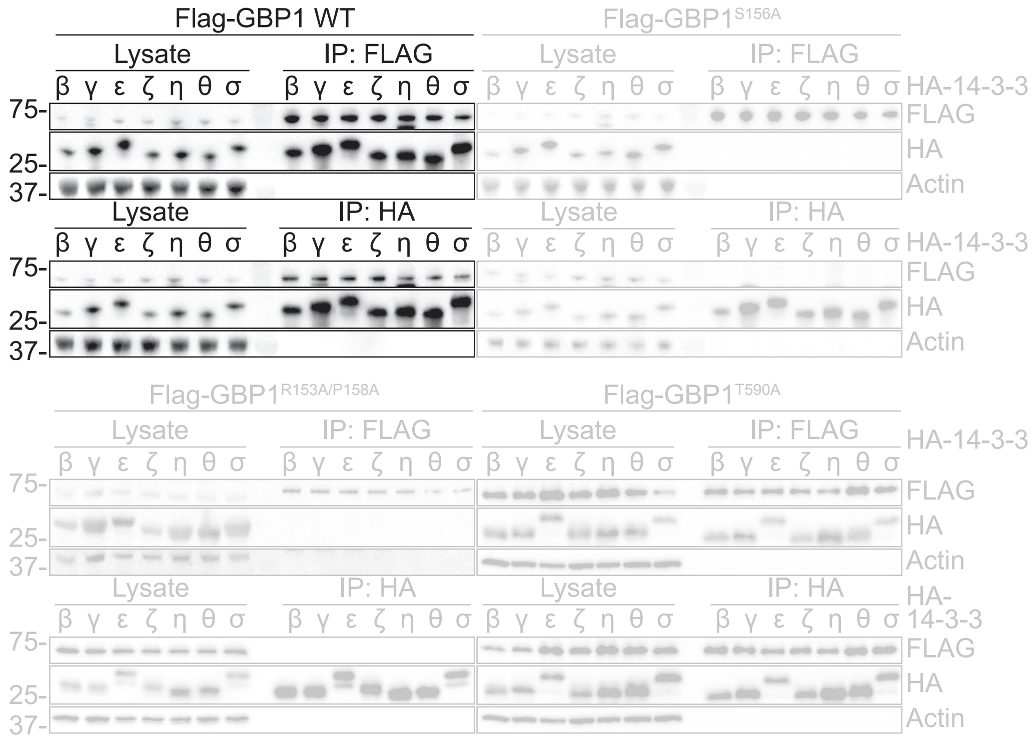

Uncropped gels with indicated size marker and crop area (lane annotation, if excluded from figure):

Flag IP GBP1 WT

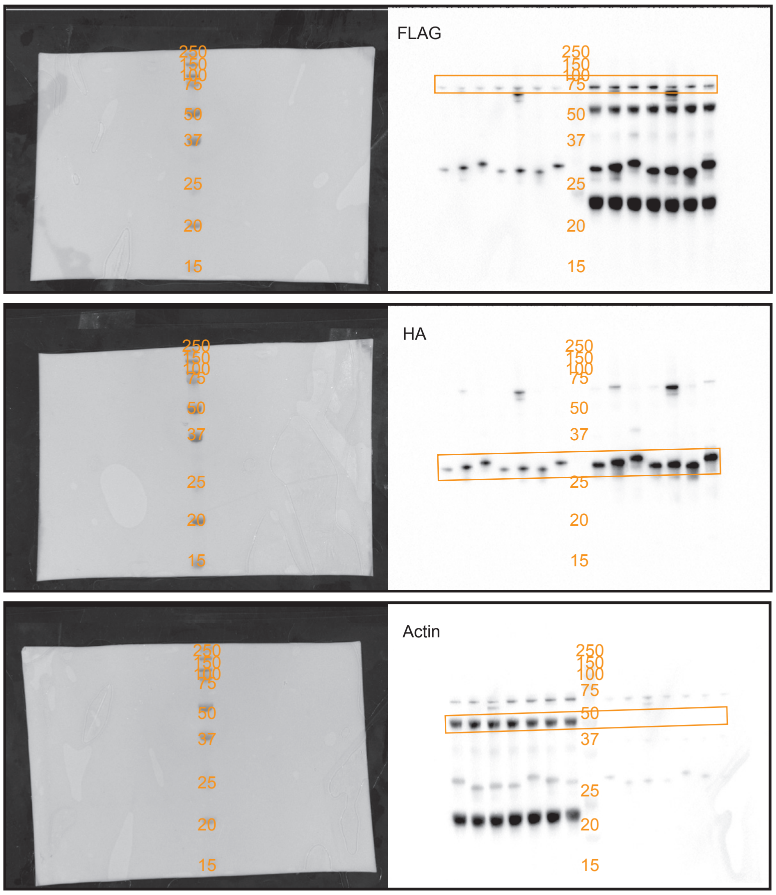

HA IP GBP1 WT

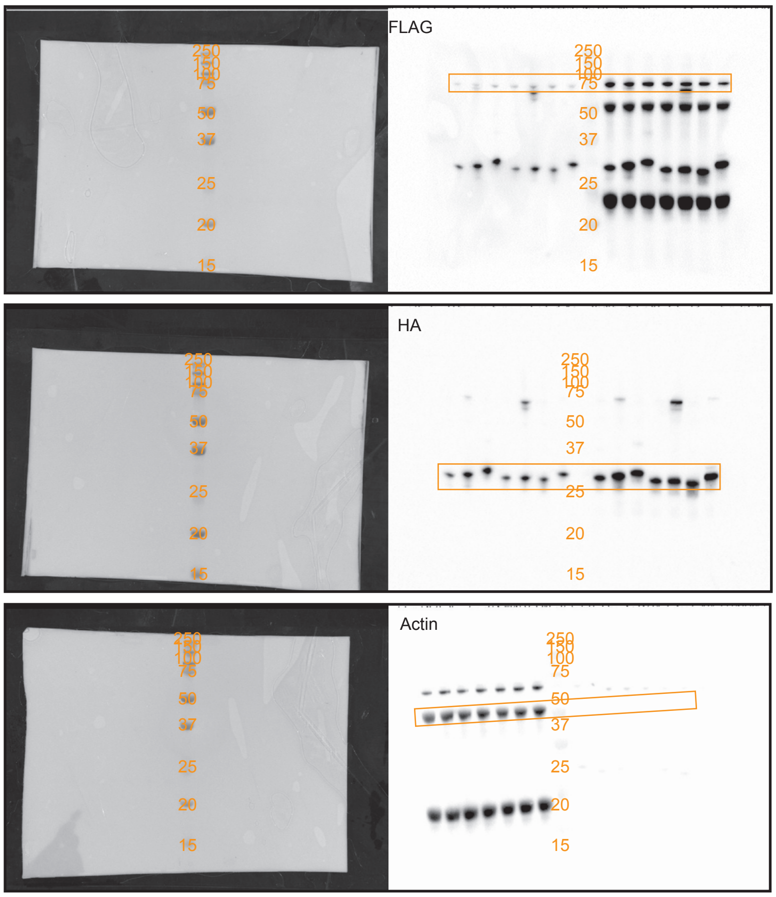

Fig. S10B - part 2

Figure as in manuscript:

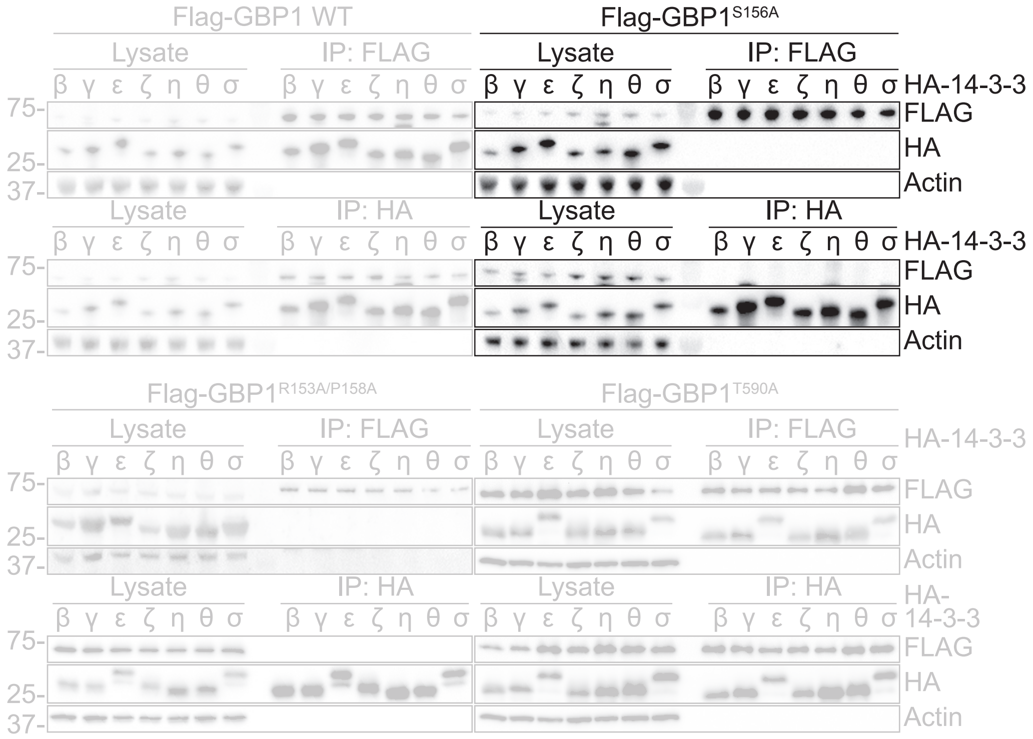

Uncropped gels with indicated size marker and crop area (lane annotation, if excluded from figure):

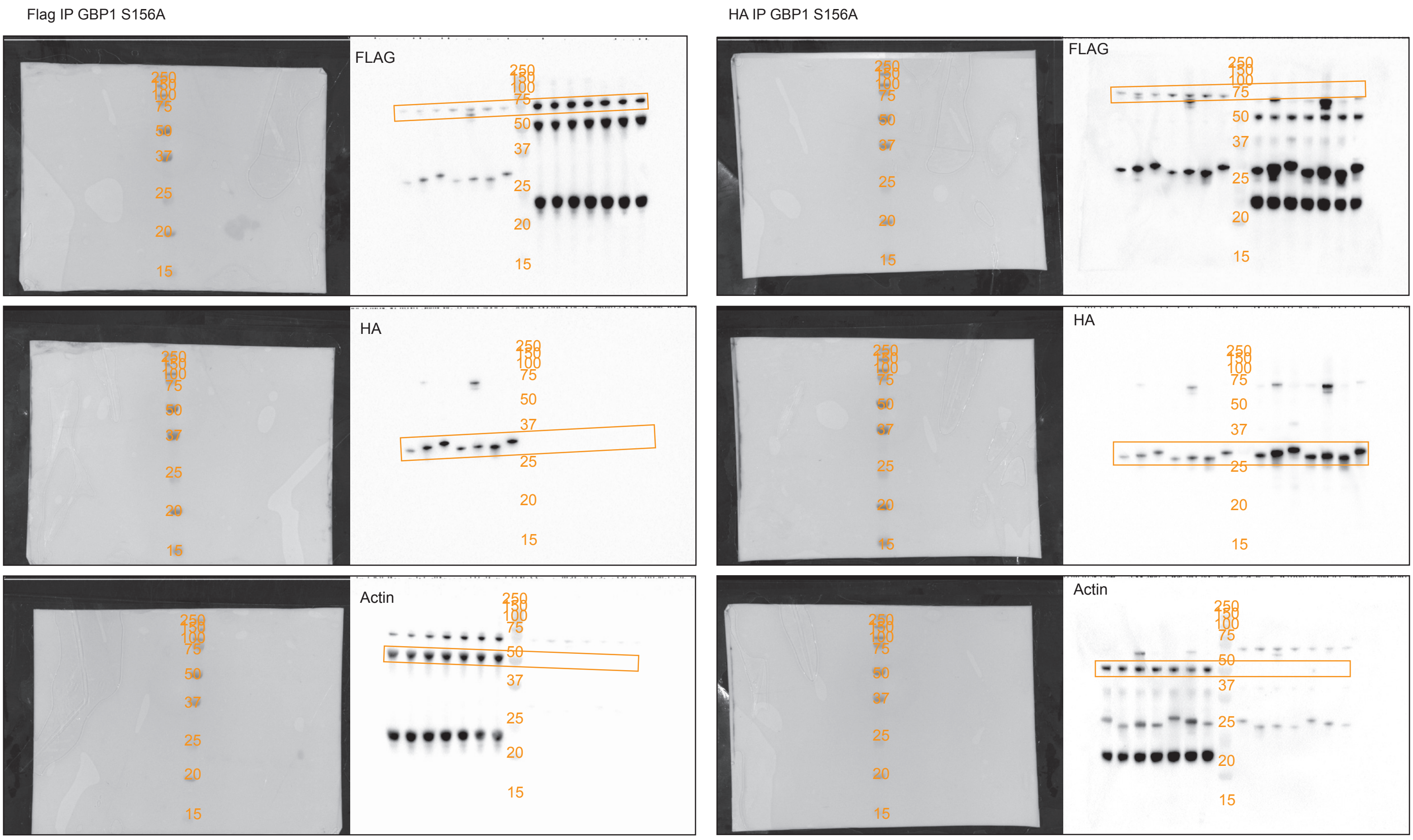

Fig. S10B - part 3

Figure as in manuscript:

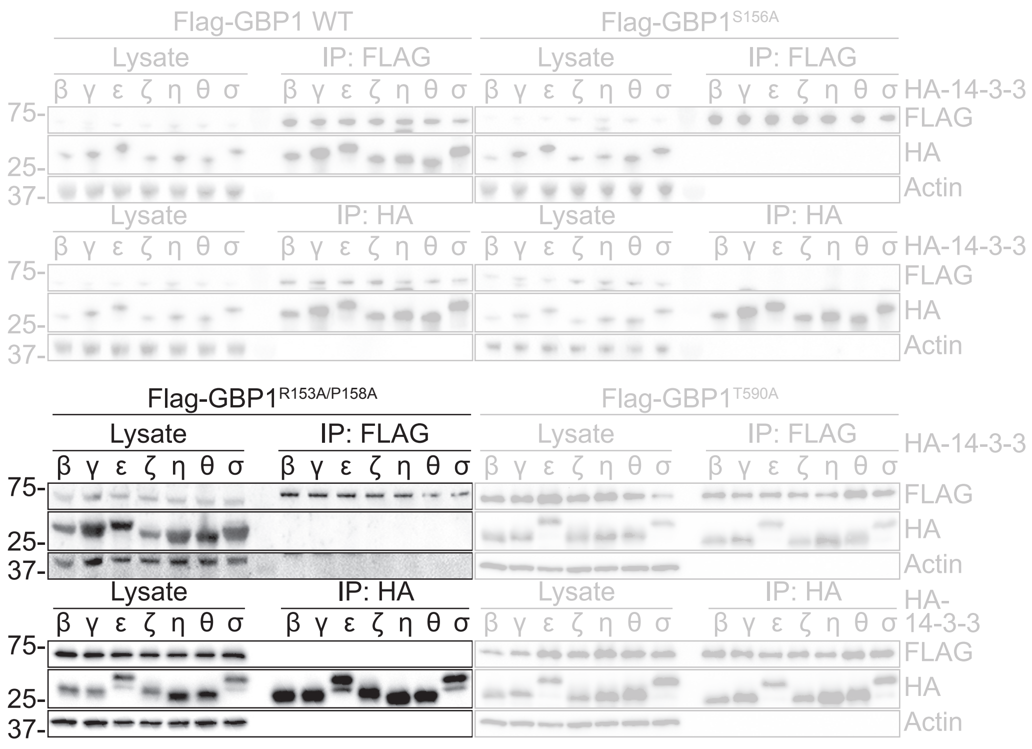

Uncropped gels with indicated size marker and crop area (lane annotation, if excluded from figure):

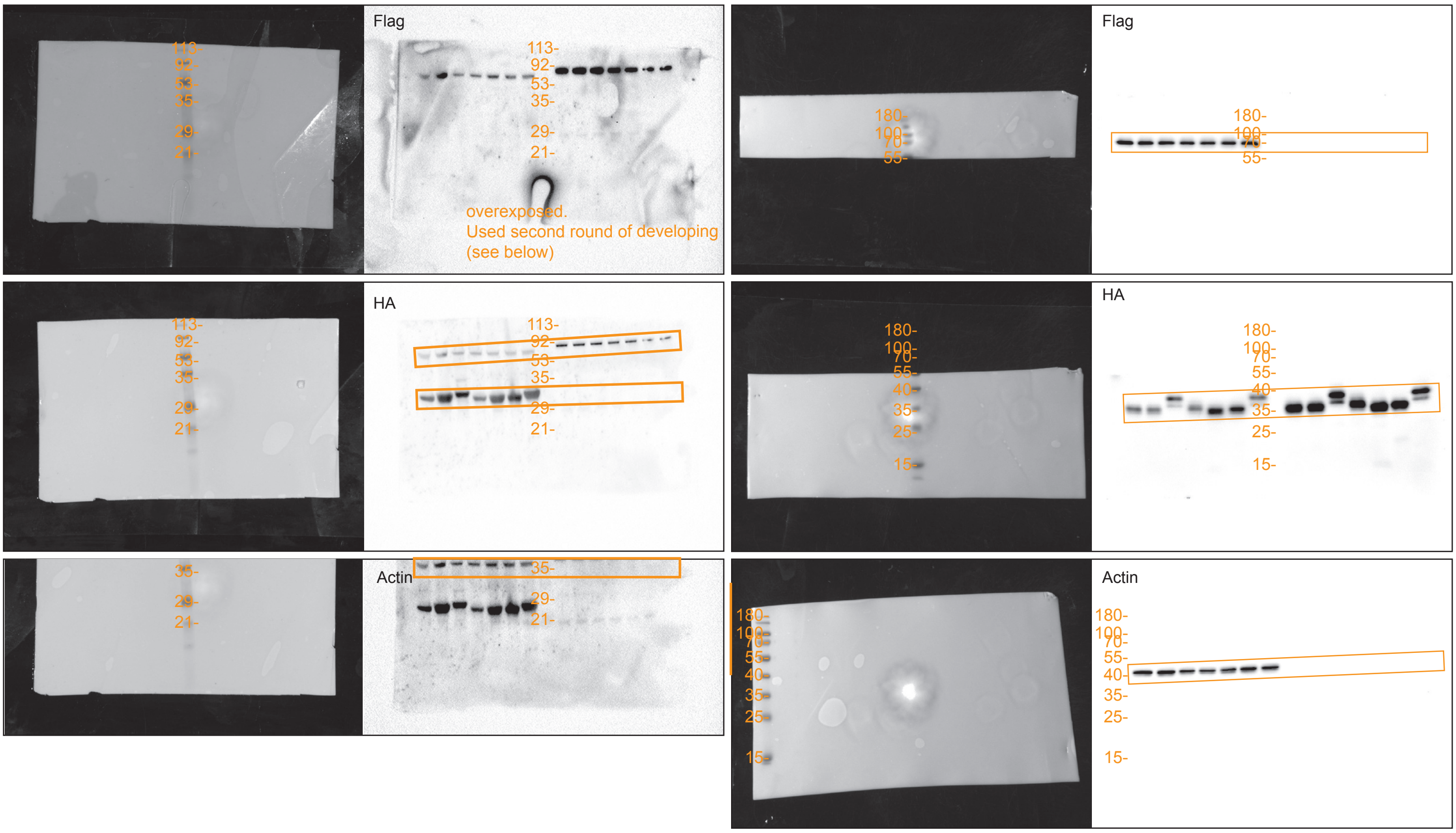

Fig. S10B - part 4

Figure as in manuscript:

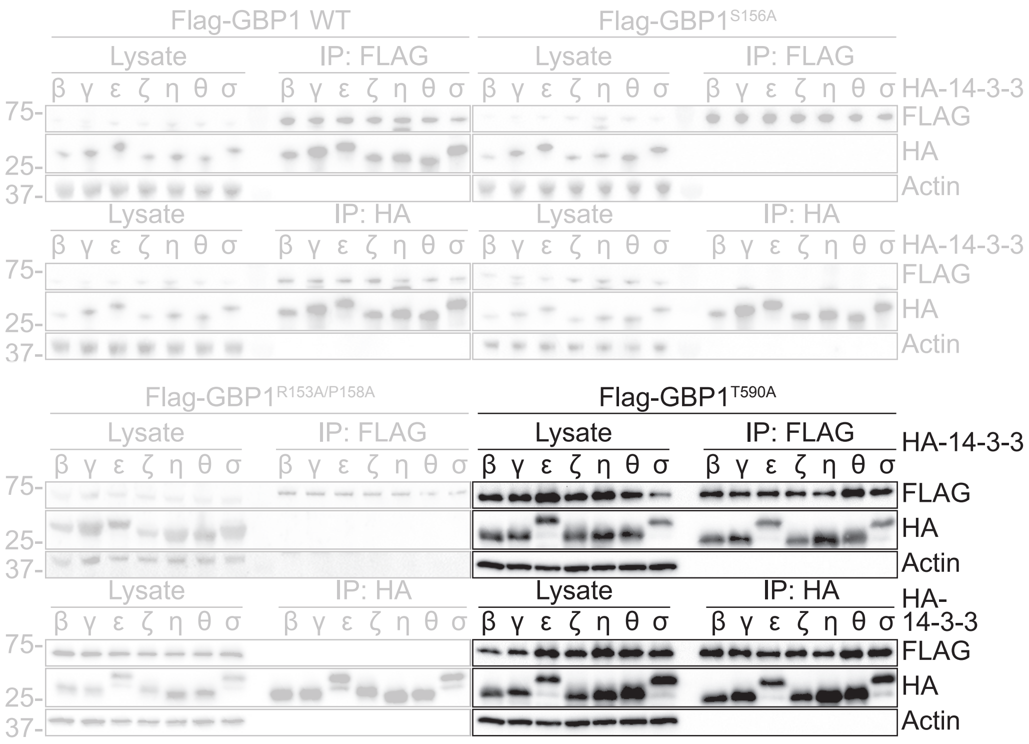

Uncropped gels with indicated size marker and crop area (lane annotation, if excluded from figure):

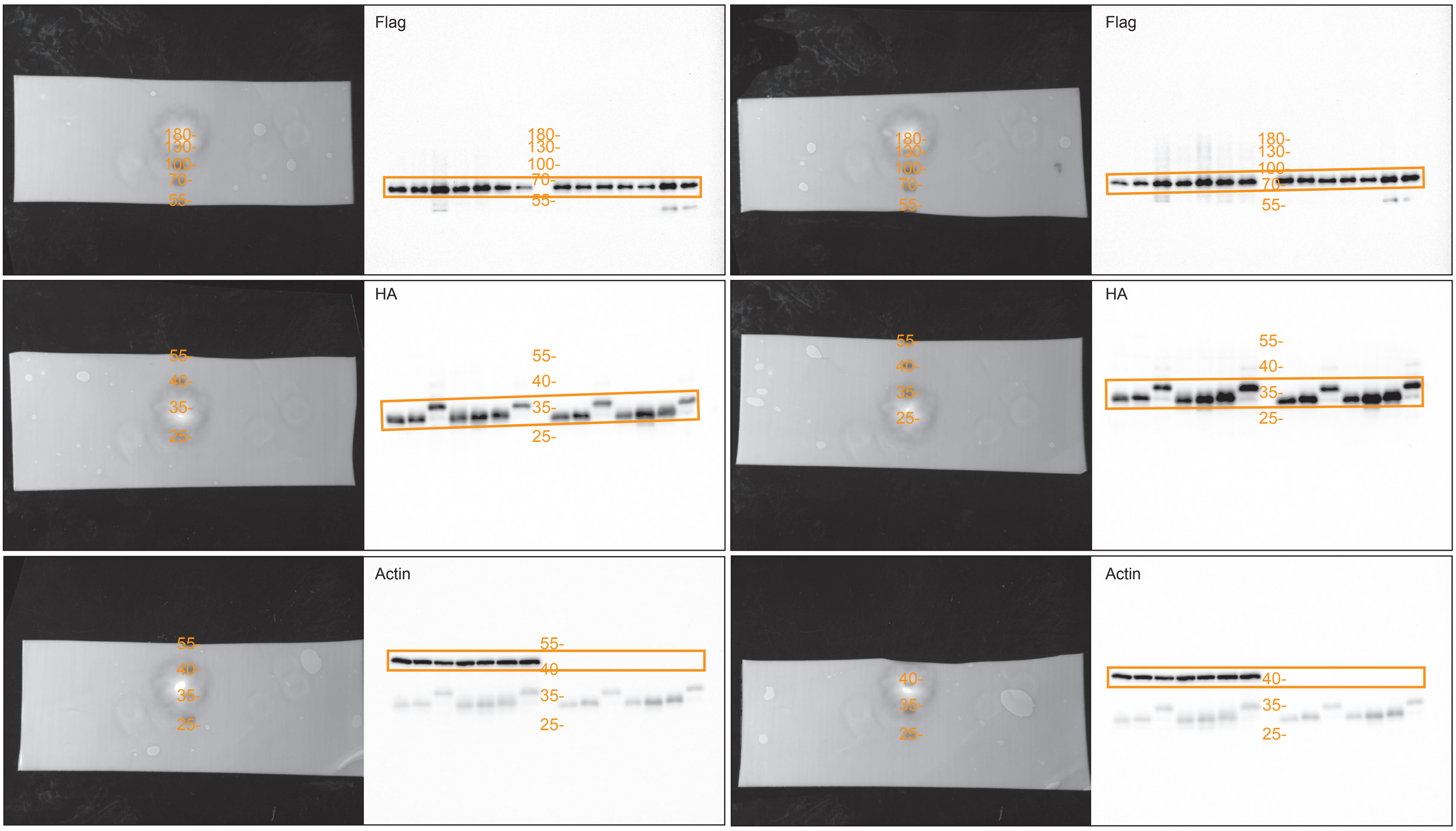

Fig. S11 - part 1

Figure as in manuscript:

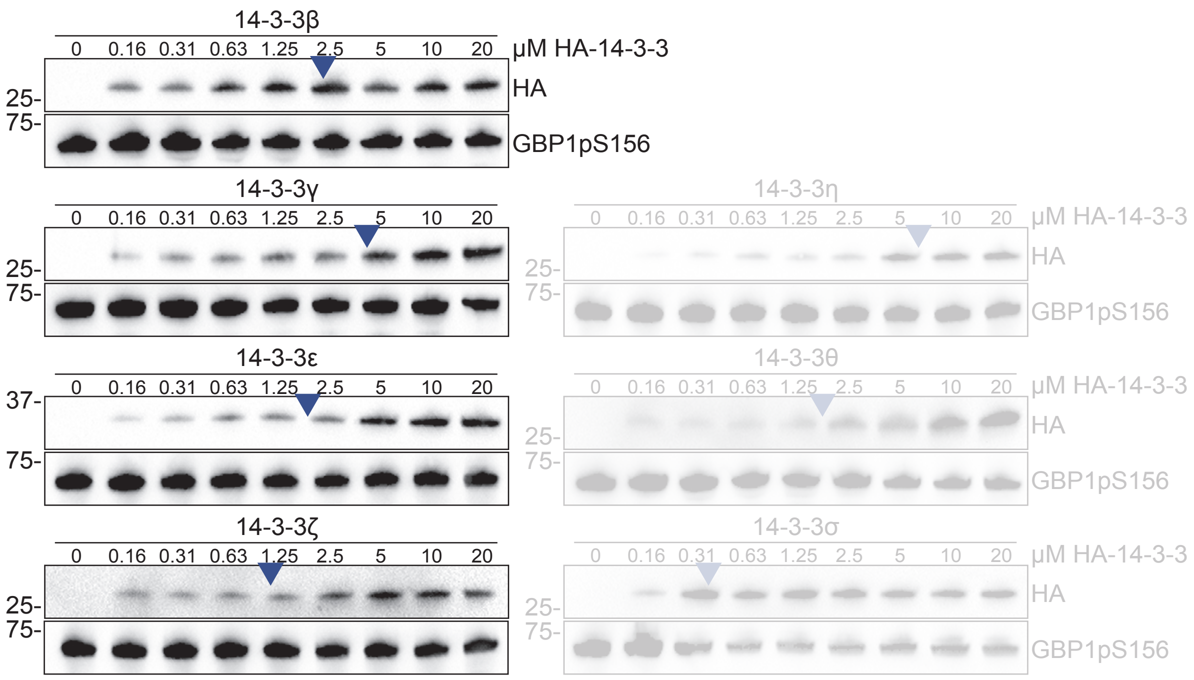

Uncropped gels with indicated size marker and crop area (lane annotation, if excluded from figure):

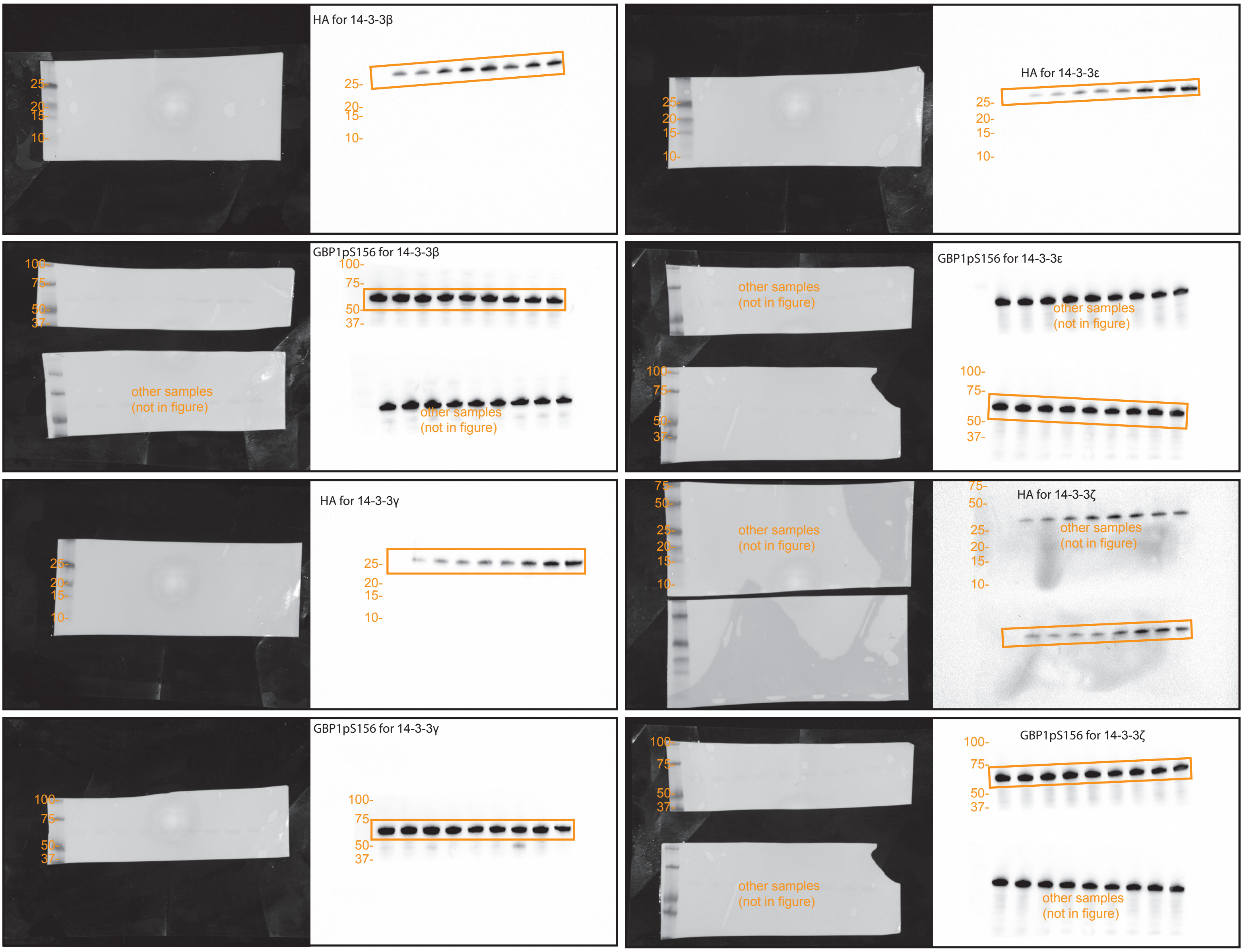

Fig. S11 - part 2

Figure as in manuscript:

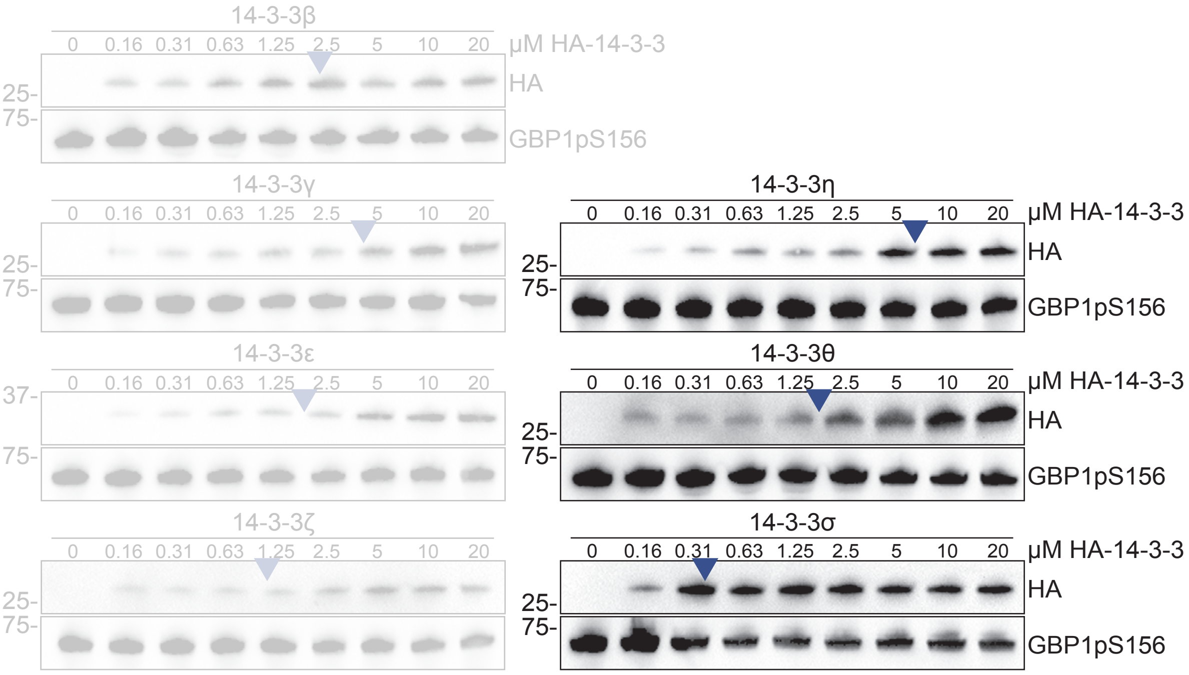

Uncropped gels with indicated size marker and crop area (lane annotation, if excluded from figure):

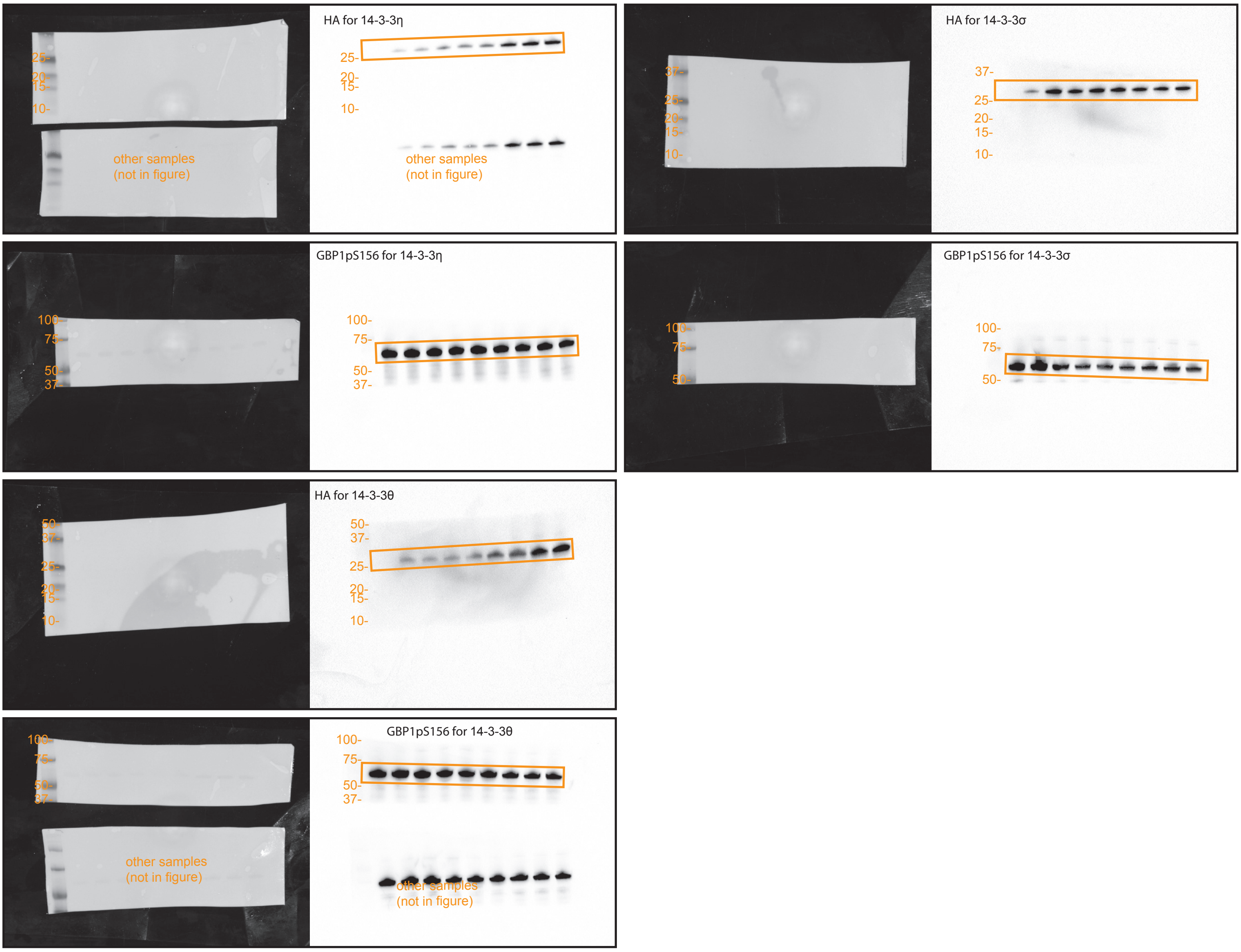

Fig. S12A

Figure as in manuscript:

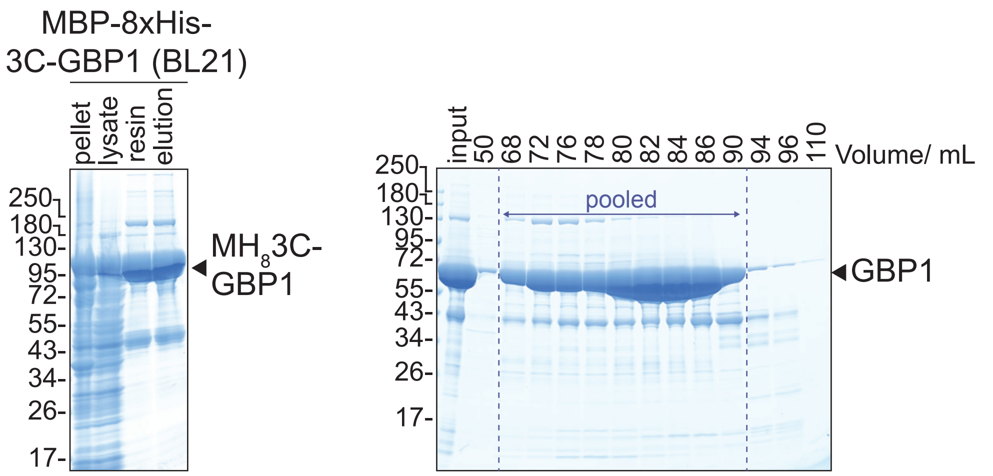

Uncropped gels with indicated size marker and crop area (lane annotation, if excluded from figure):

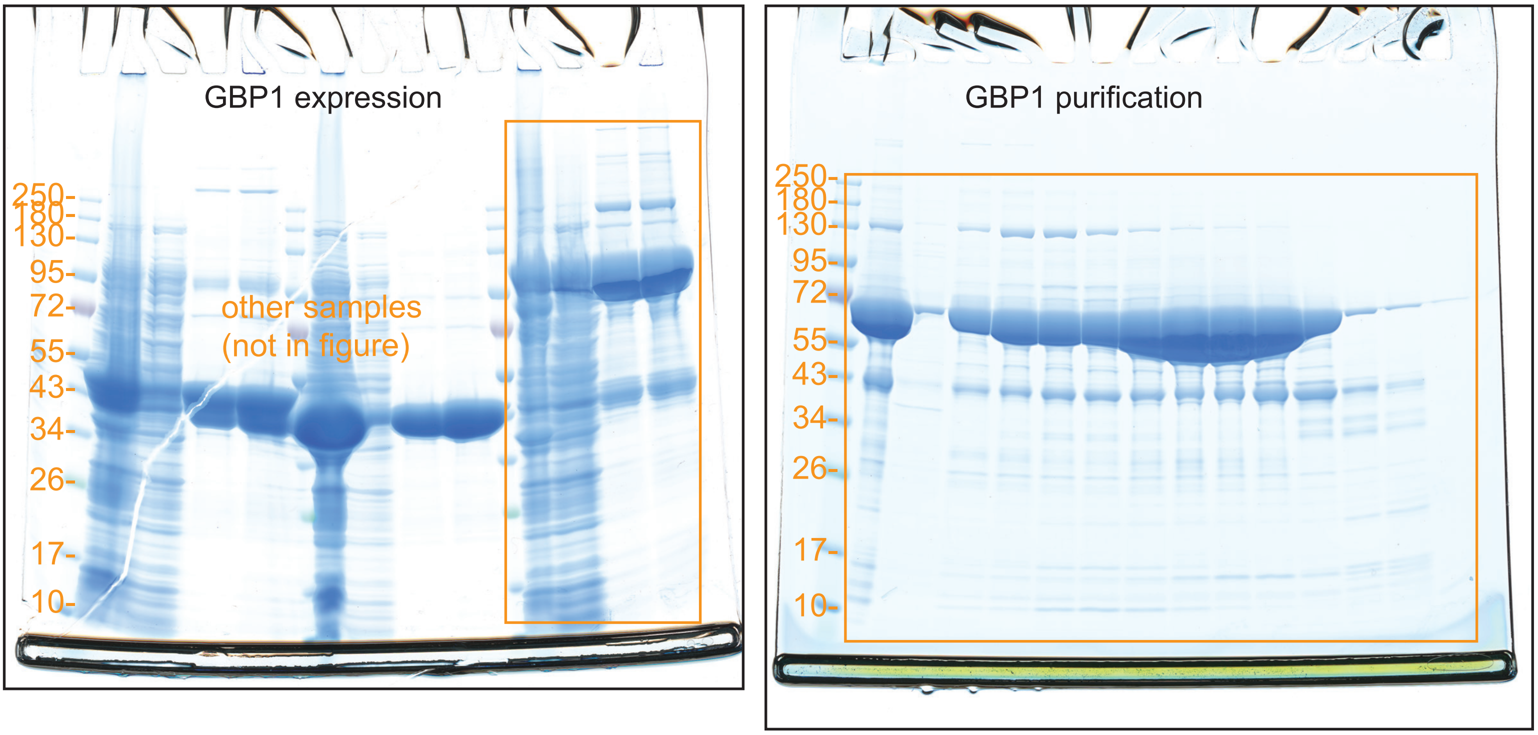

Fig. S12B

Figure as in manuscript:

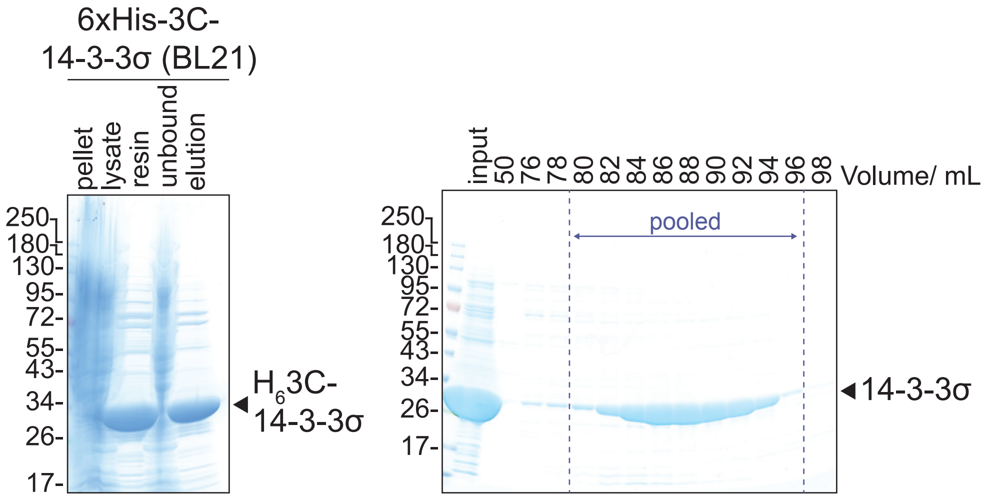

Uncropped gels with indicated size marker and crop area (lane annotation, if excluded from figure):

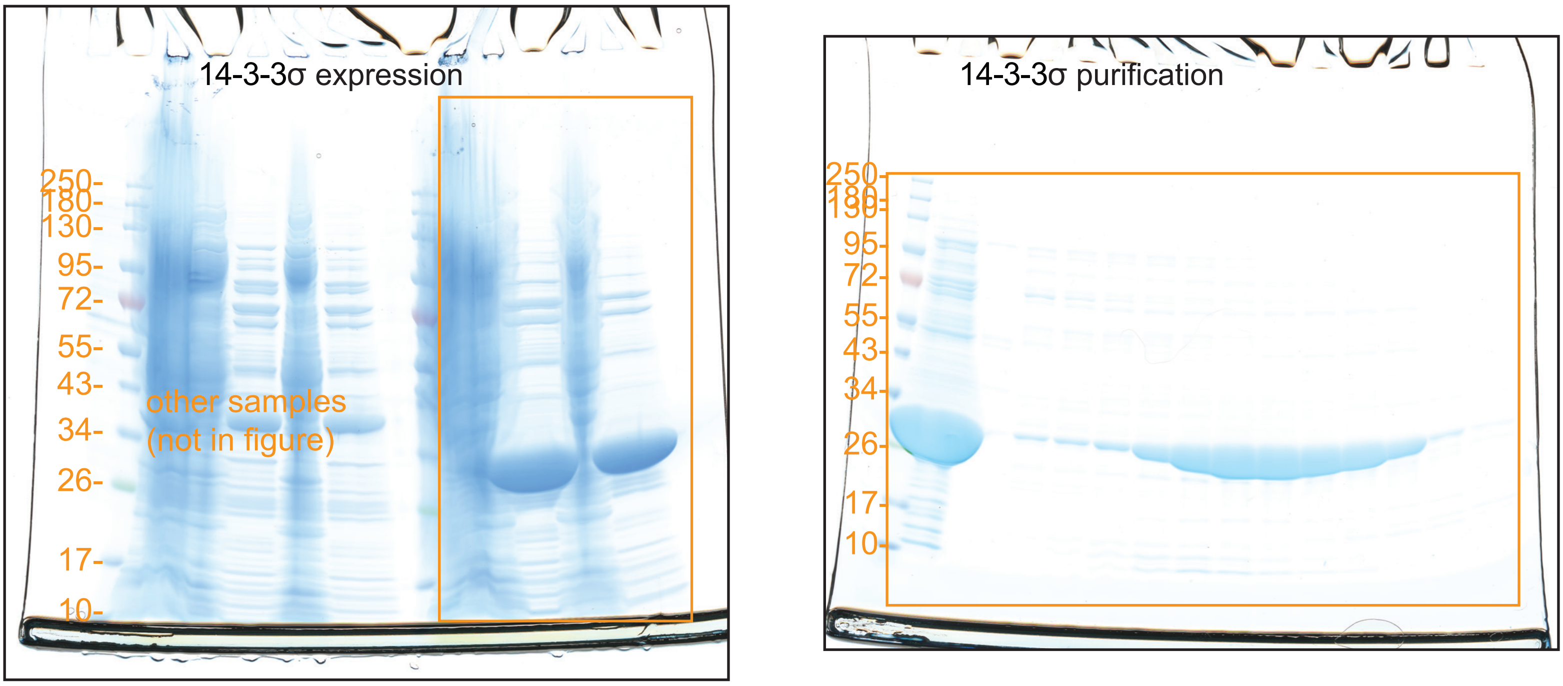

Fig. S12C

Figure as in manuscript:

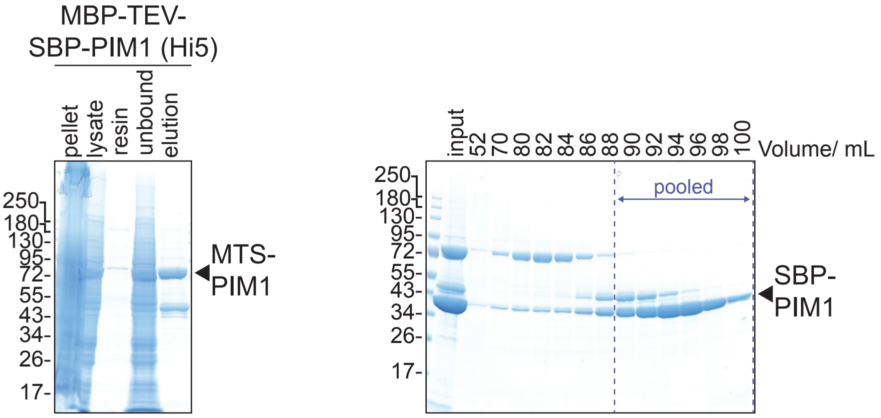

Uncropped gels with indicated size marker and crop area (lane annotation, if excluded from figure):

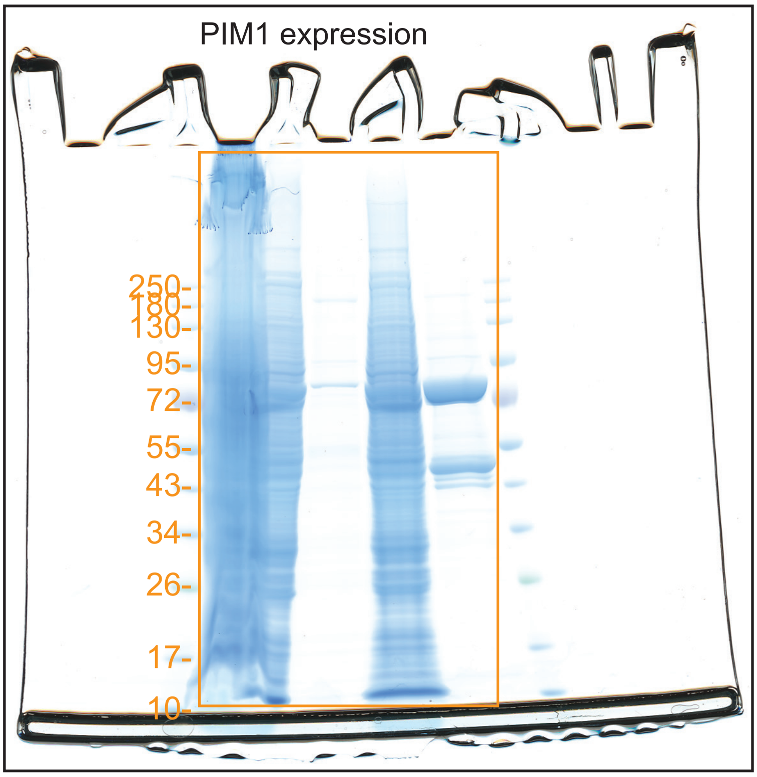

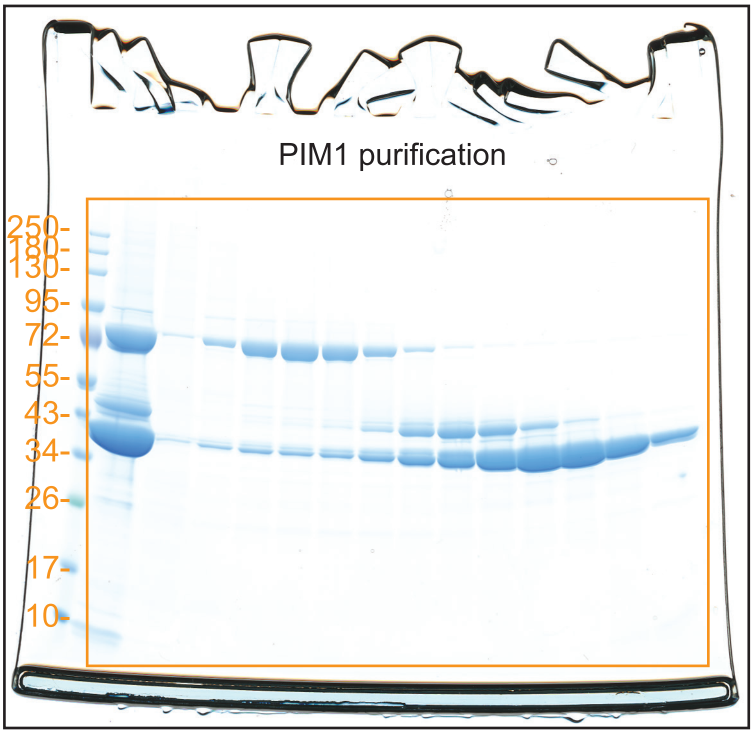

Fig. S13A

Figure as in manuscript:

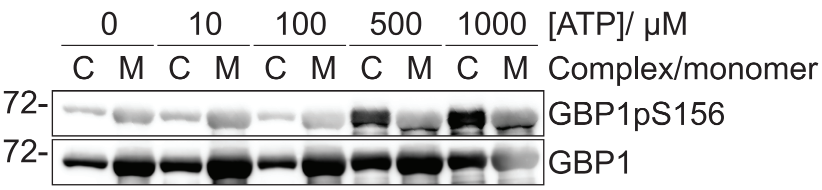

Uncropped gels with indicated size marker and crop area (lane annotation, if excluded from figure):

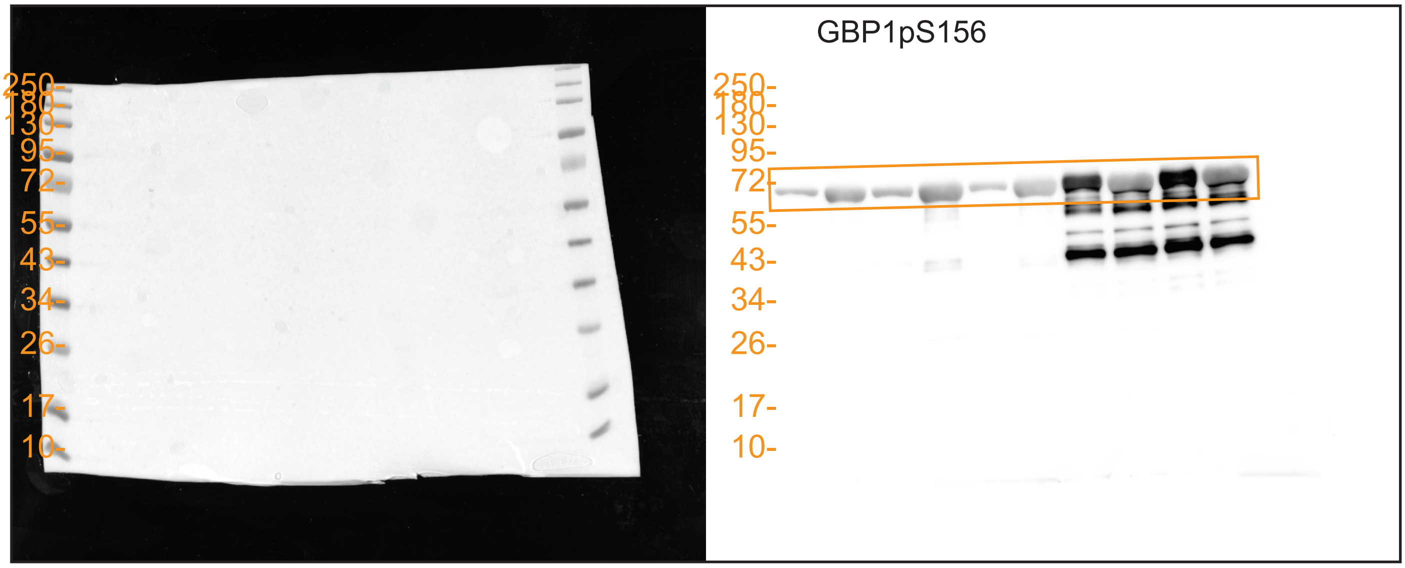

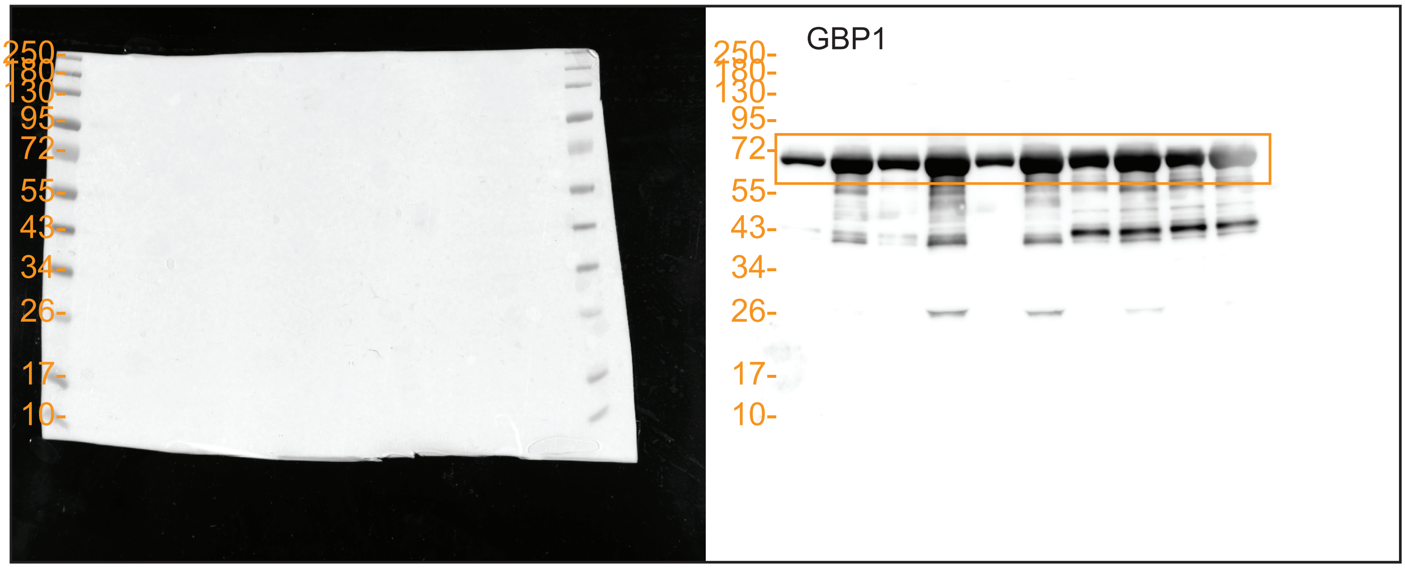

Fig. S13B

Figure as in manuscript:

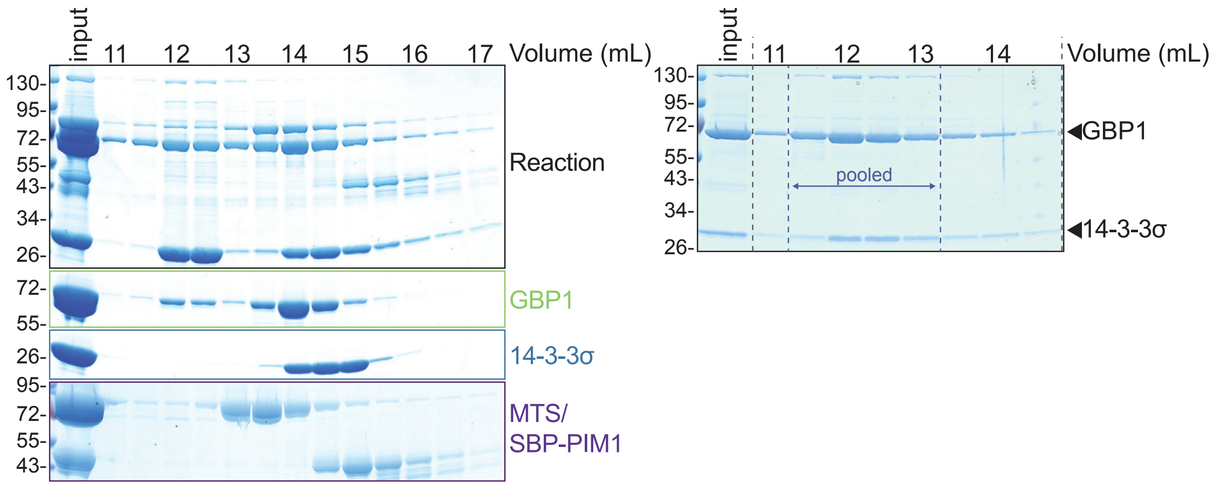

Uncropped gels with indicated size marker and crop area (lane annotation, if excluded from figure):

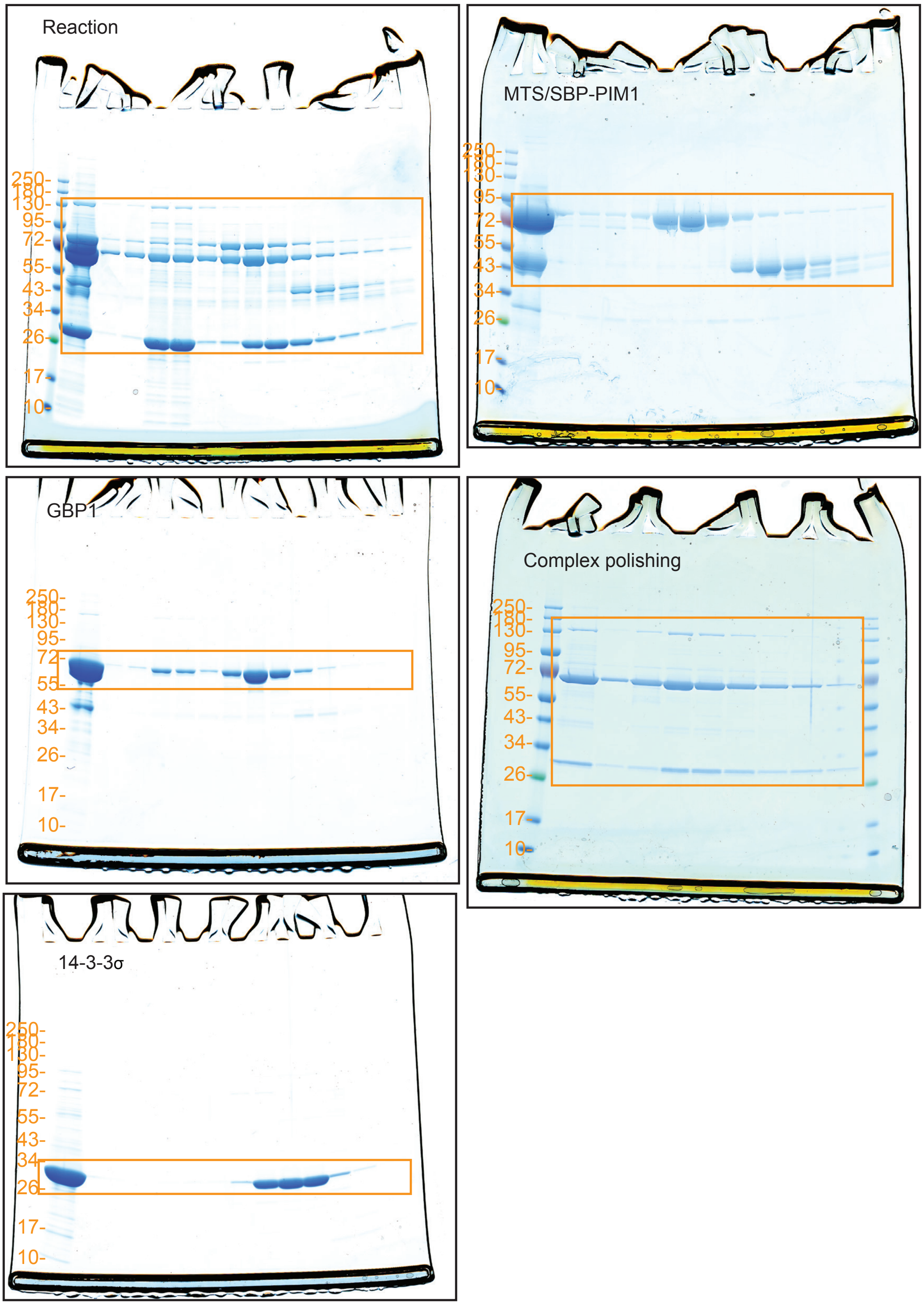

Fig. S13C

Figure as in manuscript:

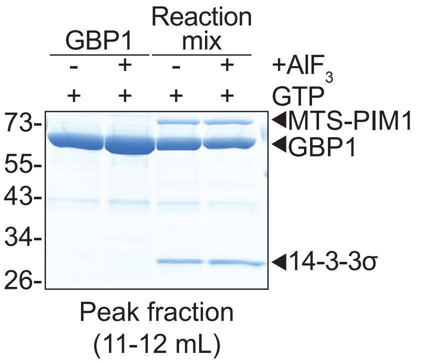

Uncropped gels with indicated size marker and crop area (lane annotation, if excluded from figure):

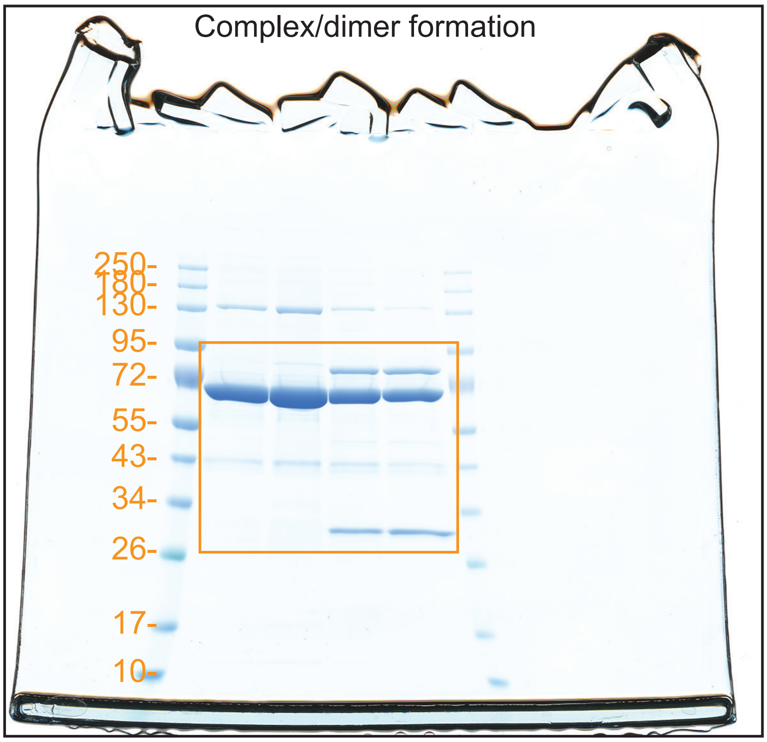

Fig. S16D

Figure as in manuscript:

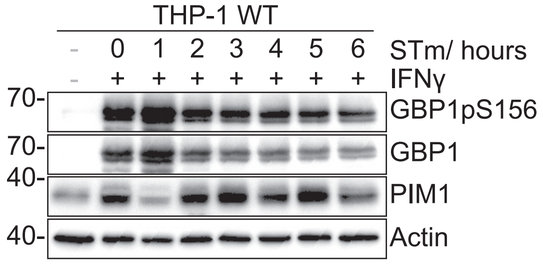

Uncropped gels with indicated size marker and crop area (lane annotation, if excluded from figure):

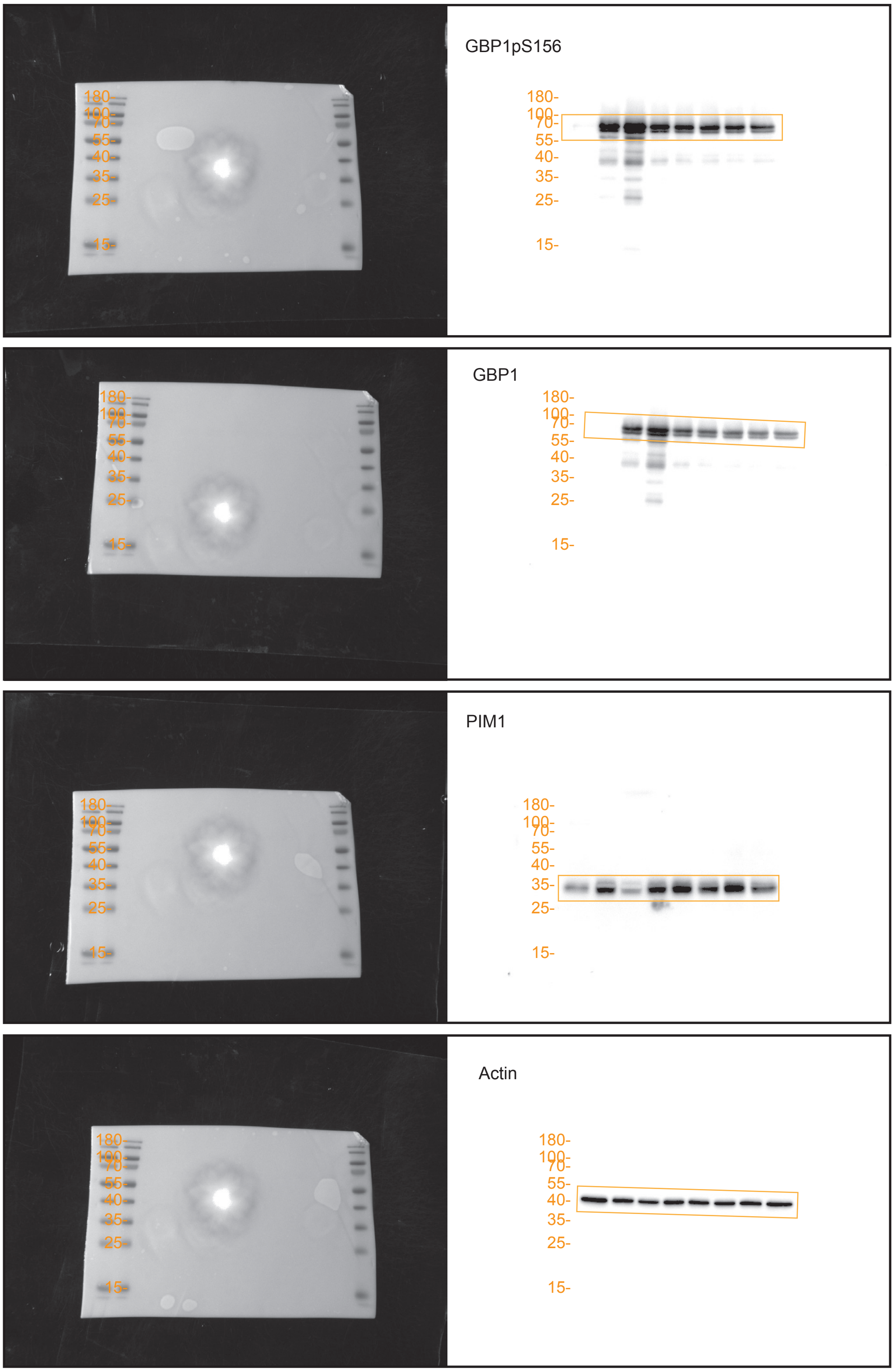

Supplement: Data S5 [file EMS189180-supplement-Data_S5.pdf]
