# Supplementary material for: PIM1 controls GBP1 activity to limit self-damage and to guard against pathogen infection
Source: Science. Author manuscript; Available in PMC 2023 Oct 12. (PMC7615196; doi:10.1126/science.adg2253)
Supplement: Supplementary Material [file EMS189180-supplement-Supplementary_Material.pdf]

Science
AAAS

Supplementary Materials for

# PIM1 controls GBP1 activity to limit self-damage and to guard against pathogen infection

Daniel Fisch, Moritz M Pfleiderer, Eleni Anastasakou, Gillian M Mackie, Fabian Wendt, Xiangyang Liu, Barbara Clough, Samuel Lara-Reyna, Vesela Encheva, Ambrosius P Snijders, Hironori Bando, Masahiro Yamamoto, Andrew D Beggs, Jason Mercer, Avinash R Shenoy, Bernd Wollscheid, Kendle M Maslowski, Wojtek P Galej, Eva-Maria Frickel

Correspondence to: e.frickel@bham.ac.uk

**This PDF file includes:**

Supplementary Figures S1-S16
Supplementary Tables S1-S6
Captions for Data S1 to S6

## SUPPLEMENTARY FIGURES:

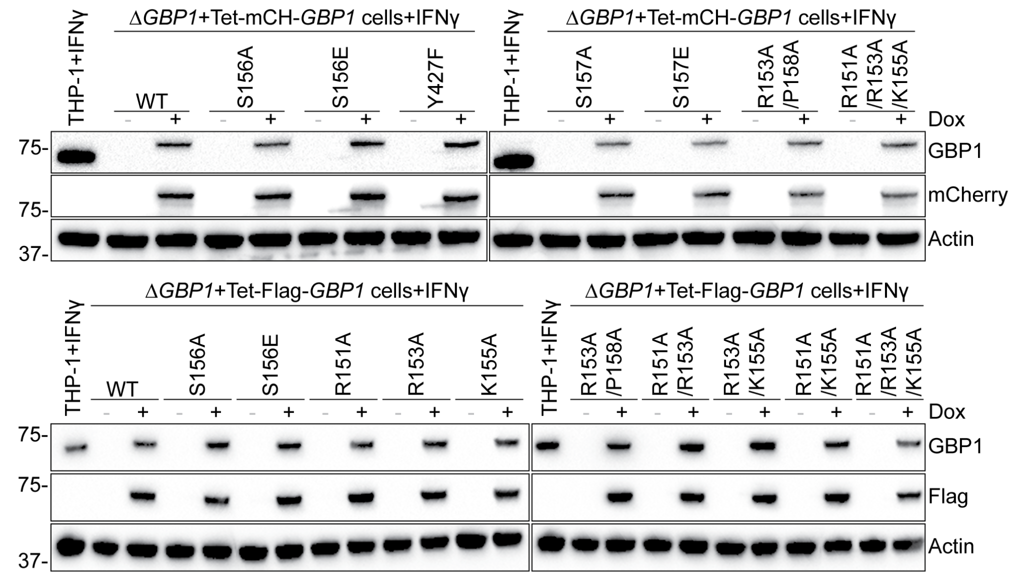


**Fig. S1: Validation of GBP1-expressing THP-1 cell lines.**

**(A)** Immunoblots of IFNγ-primed THP-1 WT, THP-1Δ*GBP1*+Tet-mCH-*GBP1* or THP-1Δ*GBP1*+Tet-Flag-*GBP1* cells expressing the indicated mutant of GBP1 under a Dox-inducible promoter. For gel source data, see **Data S5**.

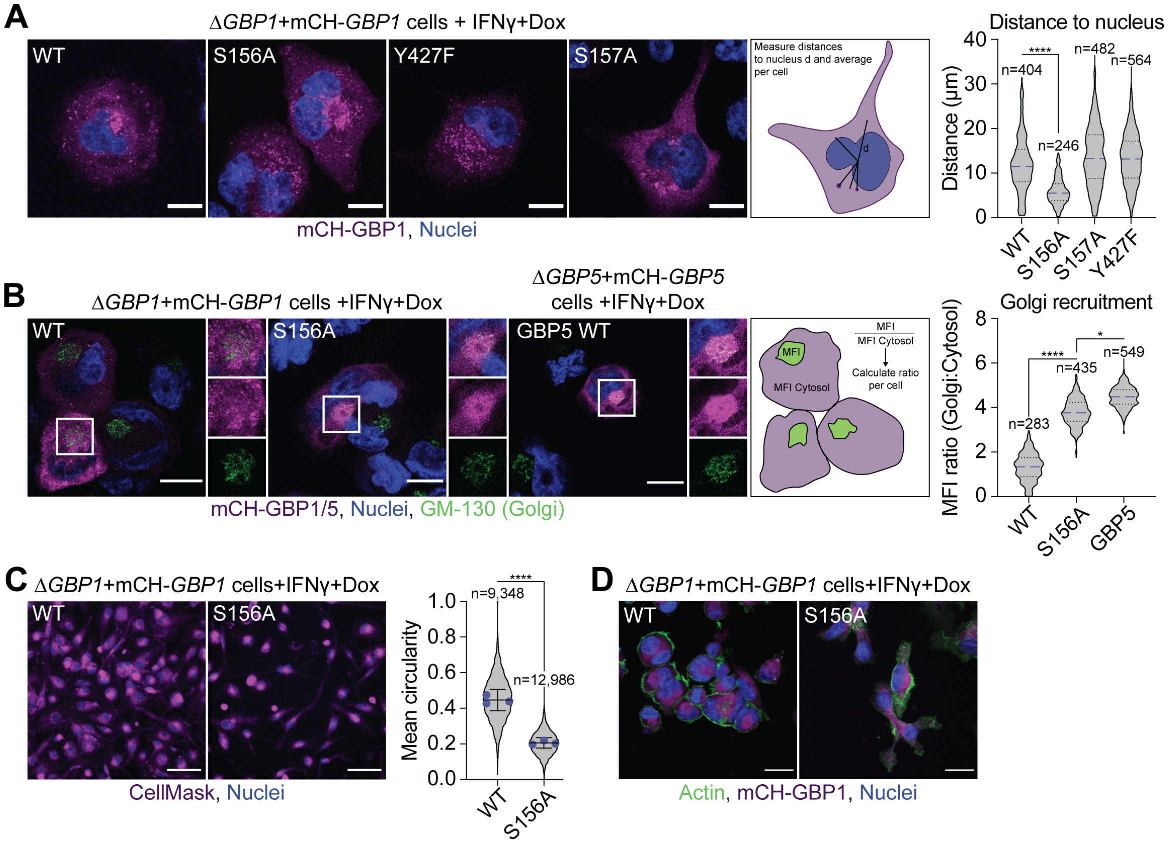


**Fig. S2: Ser156 phosphorylation changes GBP1 localization.**

**(A)** Immunofluorescence images showing localization of indicated, mCherry-tagged mutants of GBP1 or GBP1 WT, expressed in human THP-1Δ*GBP1* macrophages treated with IFNγ+Dox (left), cartoon of quantification method (middle) and quantification of average distance of GBP1 aggregates to the nucleus (right). Magenta: mCH-GBP1; Blue: Nuclei; Scale bar 10 µm. **(B)** Immunofluorescence images showing localization of mCH-GBP1 WT or S156A mutant expressed in IFNγ+Dox-treated THP-1Δ*GBP1* or mCH-GBP5 in THP-1Δ*GBP5* cells and stained for the Golgi marker GM-130 (left), cartoon of quantification method (middle) and quantification of mean fluorescence intensity (MFI) ratio of GBPs in the cytosol and the Golgi (right). Green: GM-130 (Golgi); Magenta: mCH-GBP1; Blue: Nuclei; Scale bar 10 µm. **(C)** Immunofluorescence images showing appearance of IFNγ+Dox-treated THP-1Δ*GBP1* cells expressing mCH-GBP1 WT or S156A mutant (left) and cell circularity quantification (right). Magenta: CellMask; Blue: Nuclei; Scale bar 100 µm. **(D)** Immunofluorescence images showing cortical actin cytoskeleton of IFNγ+Dox-treated THP-1Δ*GBP1* cells expressing mCH-GBP1 WT or S156A mutant. Green: Phalloidin; Magenta: mCH-GBP1; Blue: Nuclei; Scale bar 20 µm. **Data information:** Images in **(A-D)** representative of n = 3 experiments. Graph in **(A-C)** shows distribution of datapoints across n = 3 experiments (grey area) as well as mean of the repeats ± SD. Total number of quantified cells indicated in the figure. *** $P \leq 0.001$; **** $P < 0.0001$ for indicated comparisons in **(A-C)** from nested t-tests.

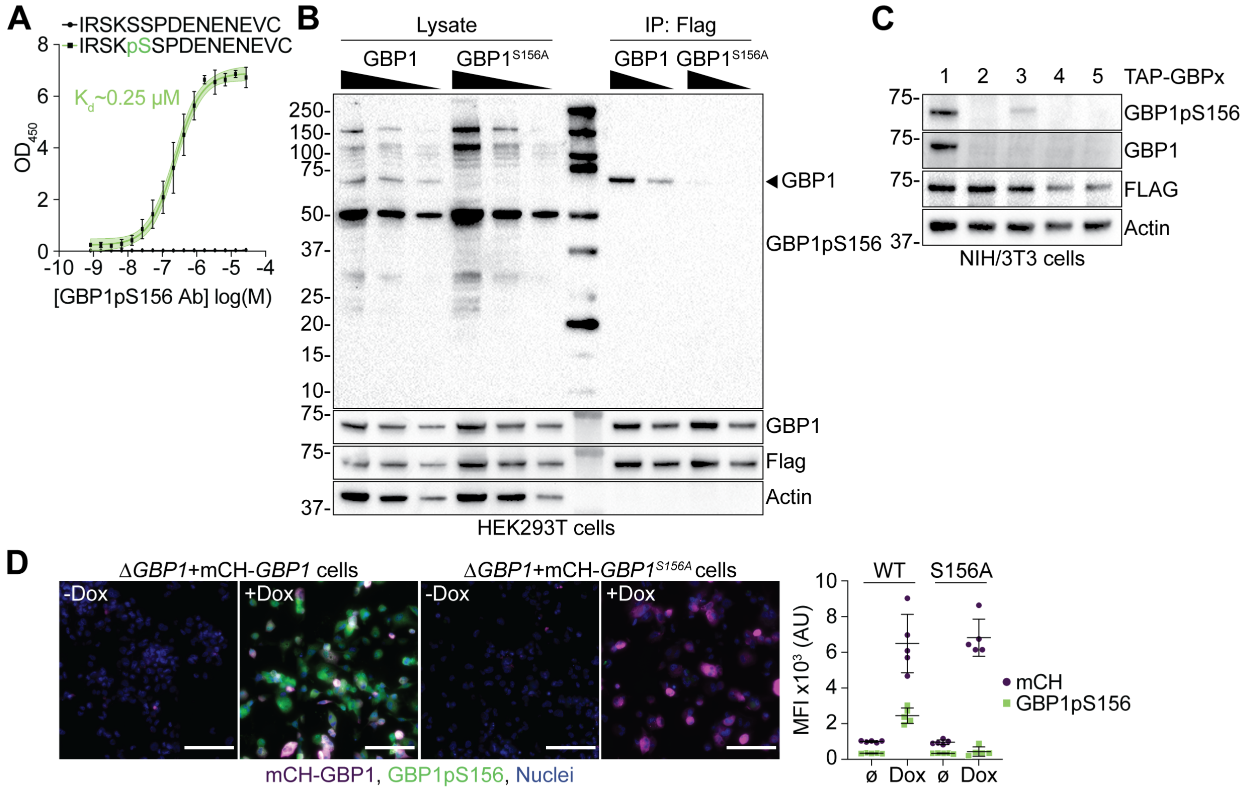


**Fig. S3: Characterization of the GBP1pS156-specific antibody.**

**(A)** In vitro binding assay determining affinity and specificity of the new GBP1pS156 antibody. **(B)** Immunoblots testing the GBP1pS156 Ab with an immunoprecipitation of Flag-GBP1 WT or Flag-GBP1$^{S156A}$ from transfected 293T cells. Arrowhead marks the bands for phosphorylated GBP1. **(C)** Representative immunoblots for GBP1 Ab or GBP1pS156 antibodies with lysates from naïve, murine NIH3T3 cells transfected with vectors for expression of tandem-affinity-purification (TAP)-tagged human GBPs. **(D)** Immunofluorescence images and quantification of mCherry (mCH) or GBP1pS156-stain fluorescence intensity per cell of IFNγ-primed THP-1Δ*GBP1*+mCH-*GBP1* WT or mCH-*GBP1*$^{S156A}$ cells treated with Dox as indicated. Magenta: mCH-GBP1; Green: GBP1pS156; Blue: nuclei. Scale bar, 100 µm. **Data information:** Graph in **(A)** shows mean ± SD from n = 3 experiments. Images in **(B)** representative of n = 2 and in **(C-D)** from n = 3 experiments. Graph in **(D)** shows mean ± SD from n = 5 experiments. For gel source data, see **Data S5**.

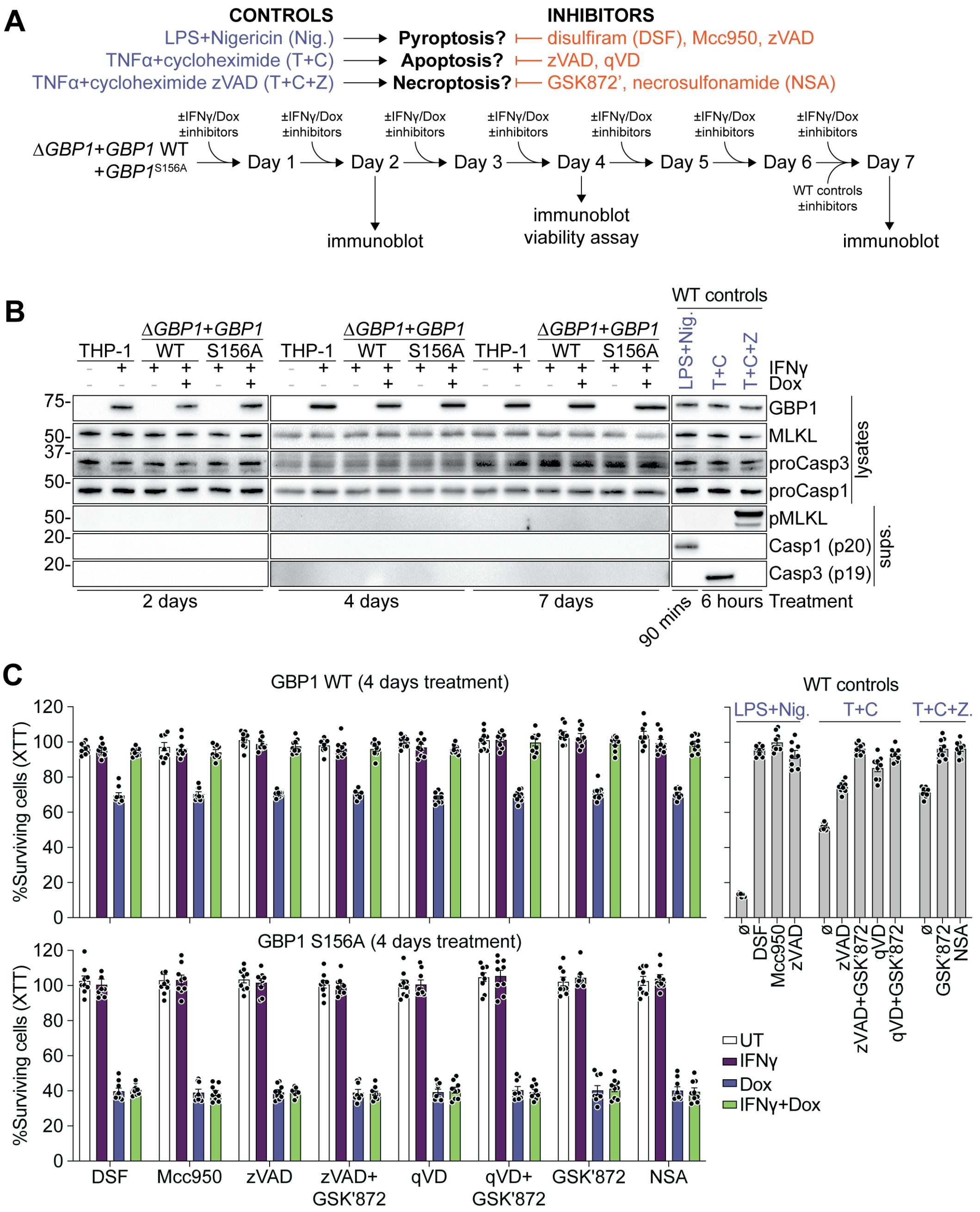


**Fig. S4: Ectopic GBP1 expression does not induce regulated cell death.**

**(A)** Overview of experimental conditions and treatments/inhibitors used to induce/block programmed cell death. **(B)** Immunoblots for markers of regulated cell death. THP-1 WT or THP-1Δ*GBP1*+*GBP1* WT or S156A expressing cells were treated with IFNγ+Dox as indicated for 2, 4 or 7 days. Regulated cell death was chemically induced by treatment with TNFα+cycloheximide (T+C) for apoptosis, Nigericin (Nig.) for pyroptosis or TNFα+cycloheximide+zVAD (T+C+Z) for necroptosis as control. **(C)** XTT cell survival assay of THP-1Δ*GBP1*+*GBP1* WT or S156A mutant expressing cells treated with IFNγ±Dox and inhibitors of programmed cell death for 4 days. **Data information:** Images in **(B)** representative of n = 3 experiments and graphs in **(C)** show mean ± SEM from n = 3 experiment. For gel source data, see **Data S5**.

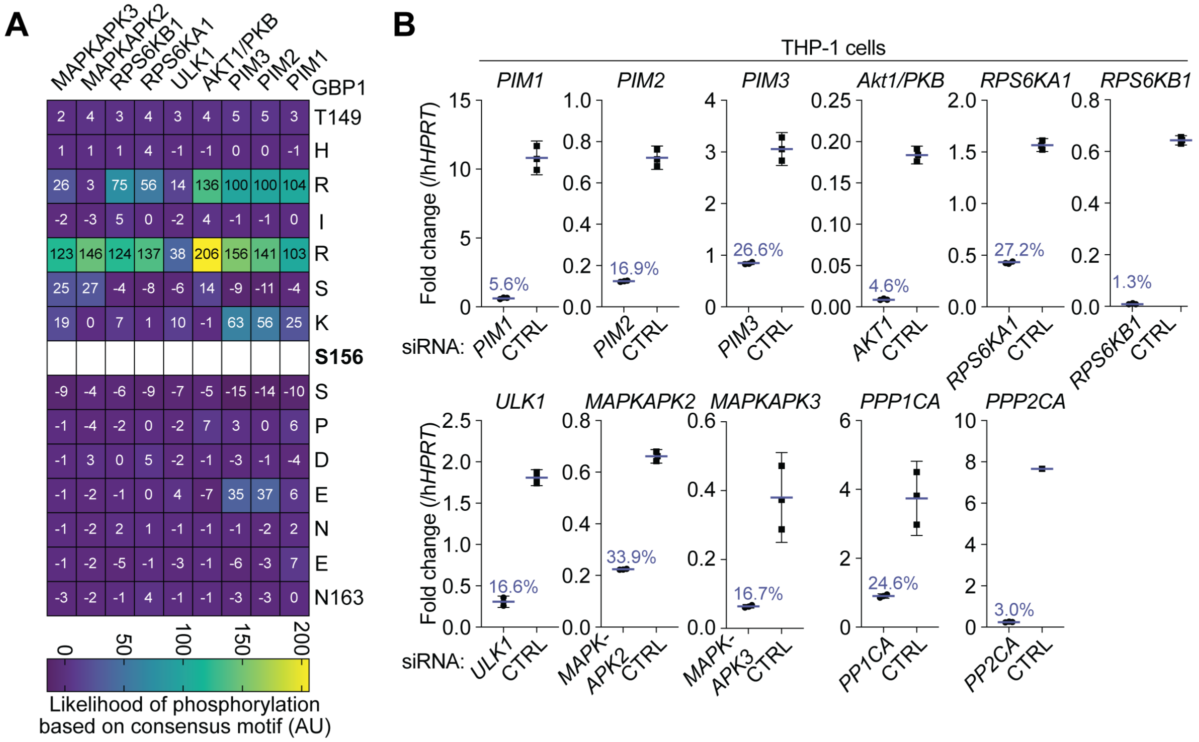


**Fig. S5: Identification of GBP1-targeting candidate kinases.**

**(A)** GBP1 Ser156 phosphorylation in silico analysis for identification of potential kinases. Diagram illustrates likelihood of motif recognition for individual kinases based on the GBP1 primary sequence. **(B)** Control of gene knockdowns using siRNA interference. Graphs showing mRNA levels (normalized to *HPRT1*) from IFNγ-primed THP-1 WT cells. Percentage mRNA levels as compared to cells transfected with non-targeting control siRNA (CTRL) are indicated. **Data information:** Graphs in **(B)** show mean ± SD from n = 3 experiments.

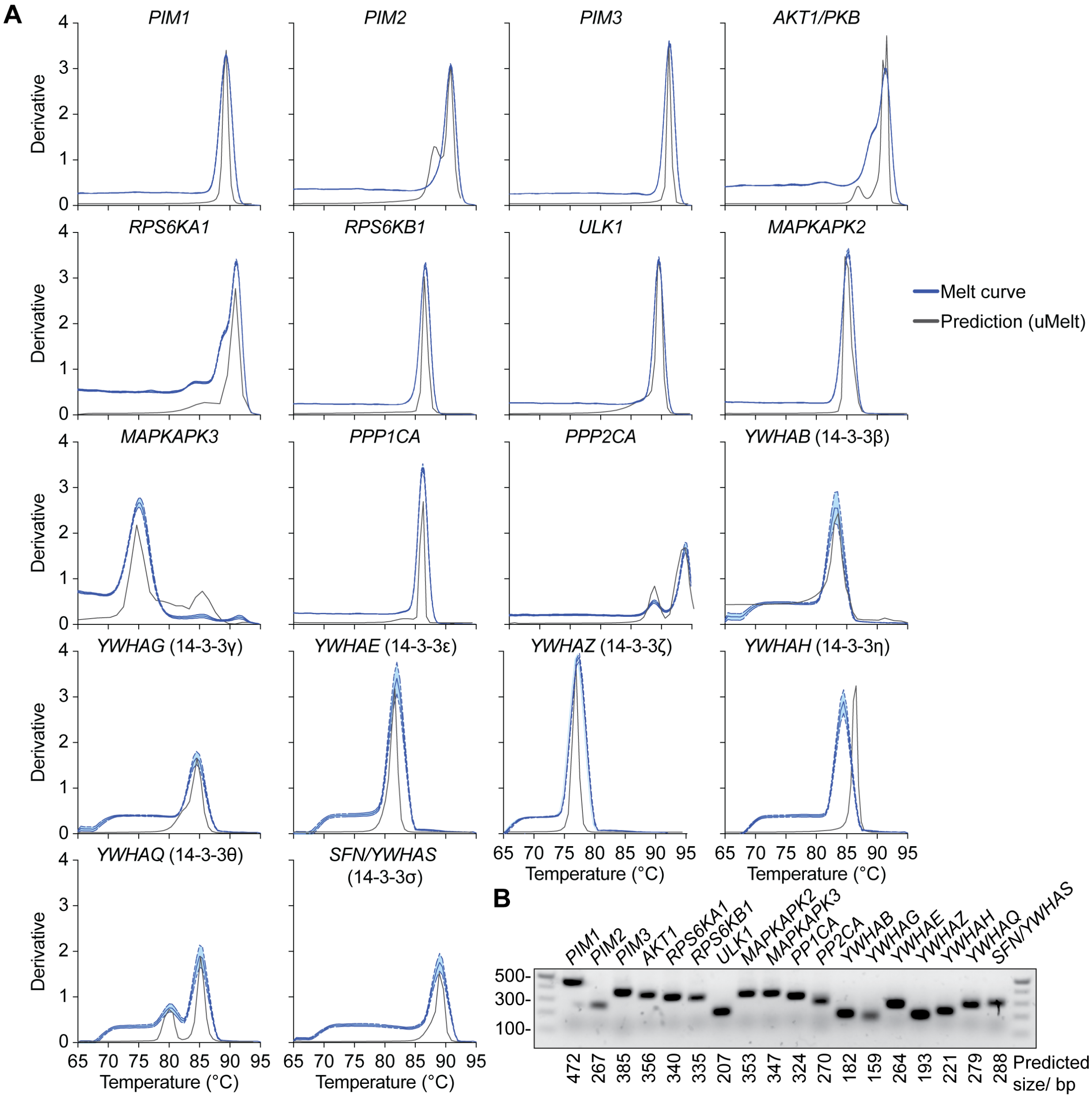


**Fig. S6: Validation of RT-qPCR primers.**

**(A)** Graphs showing measured and predicted melt curves for RT-qPCR amplicons using primer pairs for the indicated genes. **(B)** DNA gel with amplicons of the RT-qPCR primers showing specific amplification. Predicted amplicon size for the respective primer pair shown below. For gel source data, see **Data S5**.

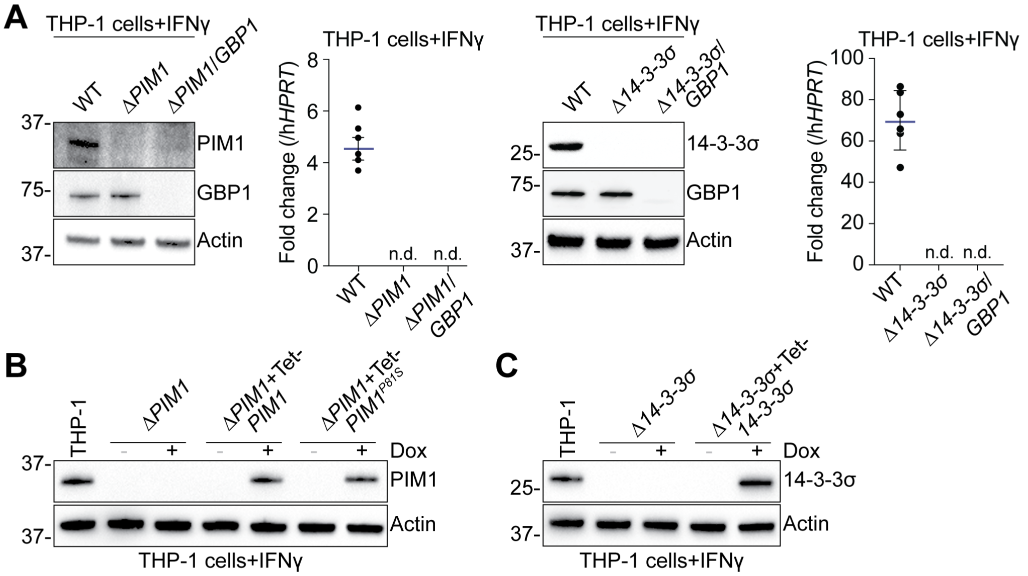


**Fig. S7: Creation of THP-1 *PIM1* and *14-3-3σ* knockout cell lines and reconstitution.**
**(A)** Immunoblots and RT-qPCR analysis of PIM1 or 14-3-3σ expression in newly created and IFNγ-primed THP WT, Δ*PIM1*, Δ*PIM1*/*GBP1*, Δ*14-3-3σ* or Δ*14-3-3σ*/*GBP1* cells; n.d. not detected. **(B)** Immunoblots of IFNγ-primed THP-1 WT or THP-1Δ*PIM1*+Tet-*PIM1* WT or kinase dead *PIM1*$^{\mathrm{P81S}}$ mutant cells treated with Dox or left untreated as indicated. **(C)** Immunoblots of IFNγ-primed THP-1 WT or THP-1Δ*14-3-3σ*+Tet-*14-3-3σ* cells treated with Dox or left untreated as indicated. For gel source data, see **Data S5**.

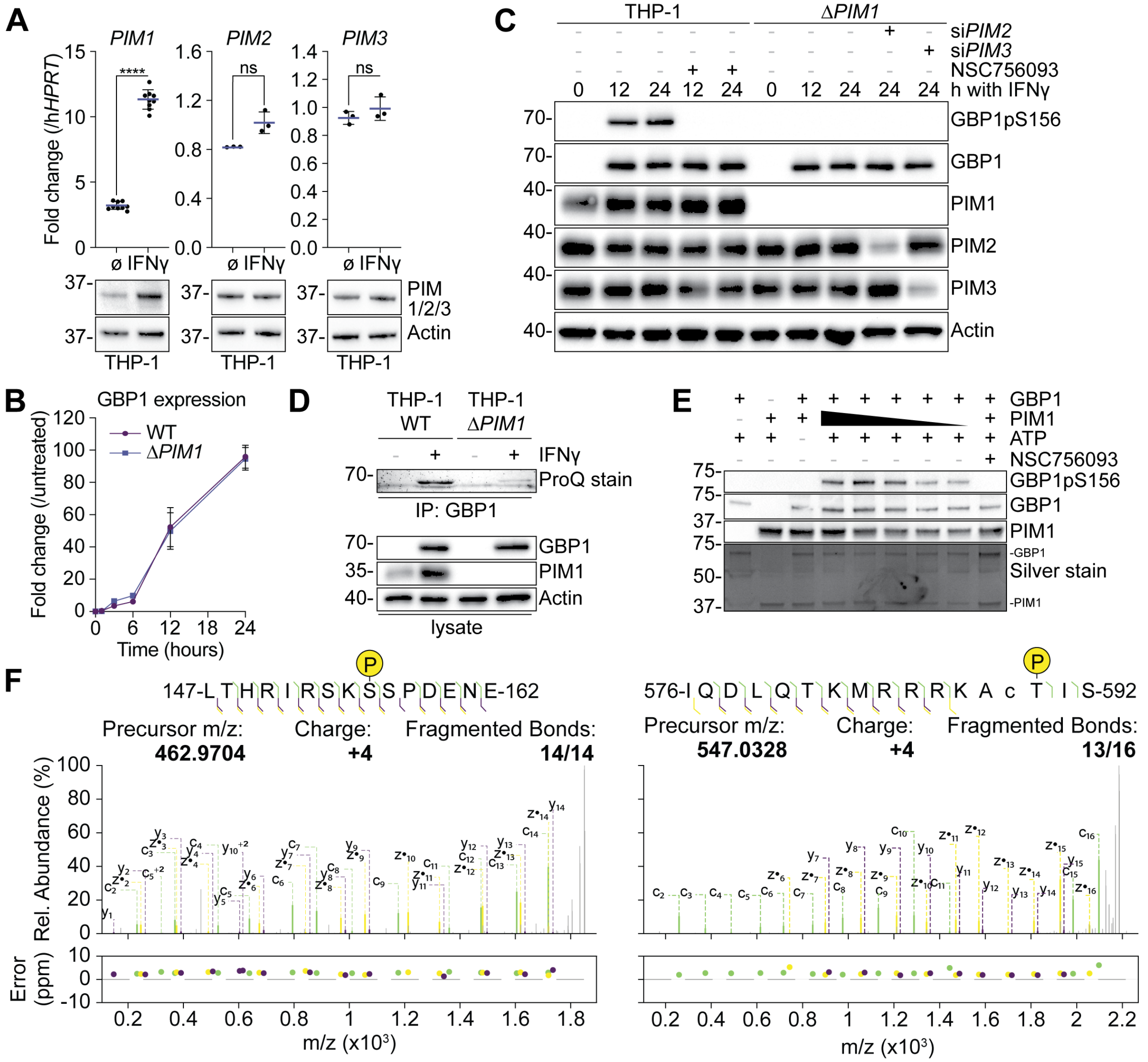

**Fig. S8: PIM1 phosphorylates GBP1 in IFNγ-primed human macrophages.**
**(A)** RT-qPCR analysis and immunoblots for PIM1/2/3 expression in naïve or IFNγ-primed THP-1 WT cells. **(B)** RT-qPCR analysis of *GBP1* mRNA expression following IFNγ-priming of THP-1 WT or Δ*PIM1* cells. **(C)** Immunoblots testing phosphorylation of endogenous GBP1 in THP-1 WT or Δ*PIM1* macrophages, primed with IFNγ and treated with NSC756093 or transfected with *PIM2* or *PIM3* siRNA. **(D)** Immunoblots and ProQ phosphoprotein stain of immunoprecipitated endogenous GBP1 in IFNγ-primed THP-1 WT or Δ*PIM1* cells. **(E)** Immunoblots and silver stain of in vitro kinase assays. **(F)** ETD-activated MS/MS spectra of peptides containing Ser156 (left) or Thr590 (right). Spectra display fragment ions used to identify the monophosphorylated peptides and localize the phosphorylation (probability > 0.9999). **Data information:** Images in **(A)** and **(C-E)** representative of n = 3 experiments. Graphs in **(A)** show mean ± SD from n = 9 experiments (PIM1) or n = 3 experiments (PIM2/3). Graph in **(B)** shows mean ± SD from n = 3 experiments. For gel source data, see **Data S5**.

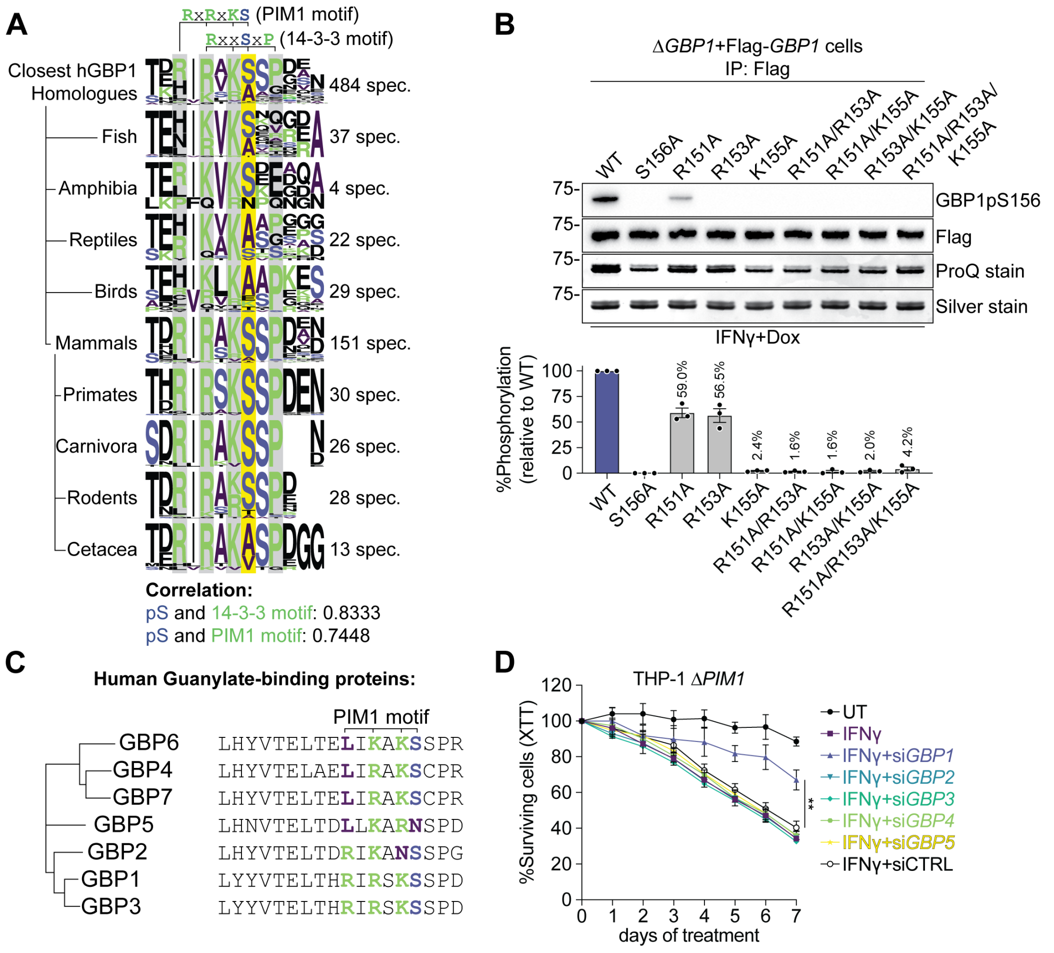


**Fig. S9: Investigation of the GBP1 kinase recognition motif.**

**(A)** Sequence conservation analysis of the closest human GBP1 homologue from 484 species. Sequence motifs show amino acid composition of the site surrounding Ser156 for the indicated class/order of species. Overall correlation between occurrence of the corresponding phosphorylation site to human Ser156 and either the 14-3-3 protein binding motif or the PIM1 recognition motif indicated below. **(B)** Immunoblots and silver/ProQ phosphoprotein gel stain of immunoprecipitated Flag-GBP1 from IFNγ+Dox-treated THP-1Δ*GBP1*+Flag-*GBP1* cells expressing the indicated mutant of GBP1 or GBP1 WT and quantification of phosphorylation level at Ser156. **(C)** Sequence alignment and phylogenetic tree of human GBP1-7. Residue equivalent to Ser156 of GBP1 highlighted in blue and basophilic kinase recognition motif in green. Disruptive amino acid replacements shown in magenta. **(D)** XTT cell survival assay of IFNγ-treated THP-1Δ*PIM1* cells transfected with the indicated siRNAs against human GBPs or non-targeting siCTRL. **Data information:** Images and graph in **(B+D)** representative of n = 3 experiments. Graph in **(B)** shows normalized ProQ signal relative to GBP1 WT (=100%) and GBP1 S156A mutant (=0%). Graph in **(D)** shows mean ± SEM from n = 3 experiments, normalized to untreated cells. ** $P \leq 0.01$ for indicated comparisons in **(D)** from one-way ANOVA For gel source data, see **Data S5**.

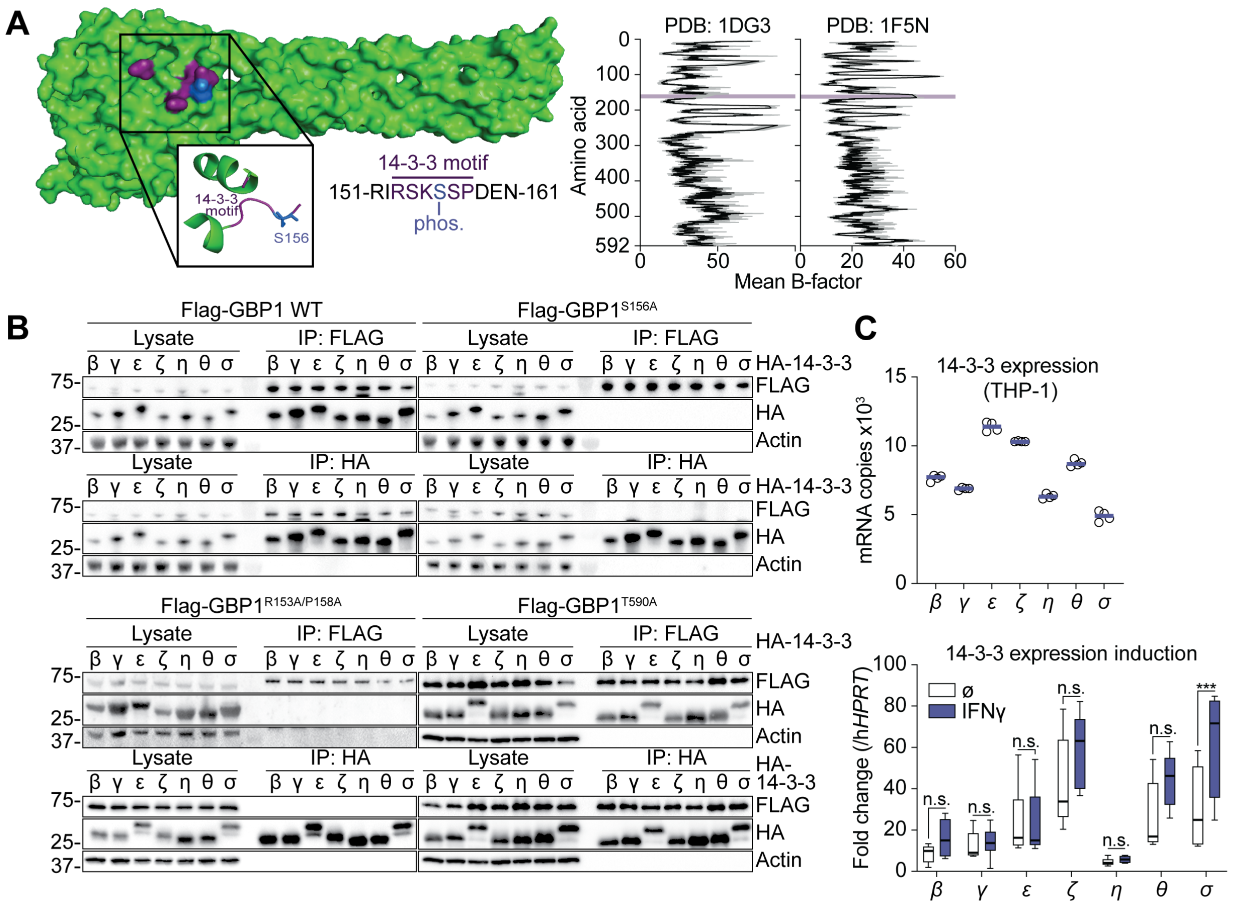


**Fig. S10: Characterization of GBP1 interaction with 14-3-3 proteins.**

**(A)** Crystal structure of human GBP1 with predicted 14-3-3-binding-site highlighted in purple and Ser156 phosphorylation site in blue and graphs showing B-factor of two GBP1 crystal structures with the phosphorylation-motif-containing loop highlighted in purple. **(B)** Immunoblots for immunoprecipitations of Flag-GBP1 WT, Flag-GBP1$^{S156A}$, Flag-GBP1$^{R153A/P158A}$ or Flag-GBP1$^{T590A}$ and reverse IP of HA-14-3-3 proteins from HEK293T cells co-transfected with vectors expressing the respective proteins. **(C)** RT-qPCR analysis of absolute 14-3-3 protein expression in naïve THP-1 WT cells as compared to plasmid standard and expression induction following IFNγ-stimulation. **Data information:** Images in **(B)** representative of n = 3 experiments. Graphs in **(C)** show mean of n = 4 experiments (14-3-3 expression) or box plot with median and interquartile range (relative expression induction; n = 3). *** $P \leq 0.001$ in **(C)** comparing IFNγ-treated to naïve cells from 2-way ANOVA following adjustment for multiple comparisons; n.s. not significant. For gel source data, see **Data S5**.

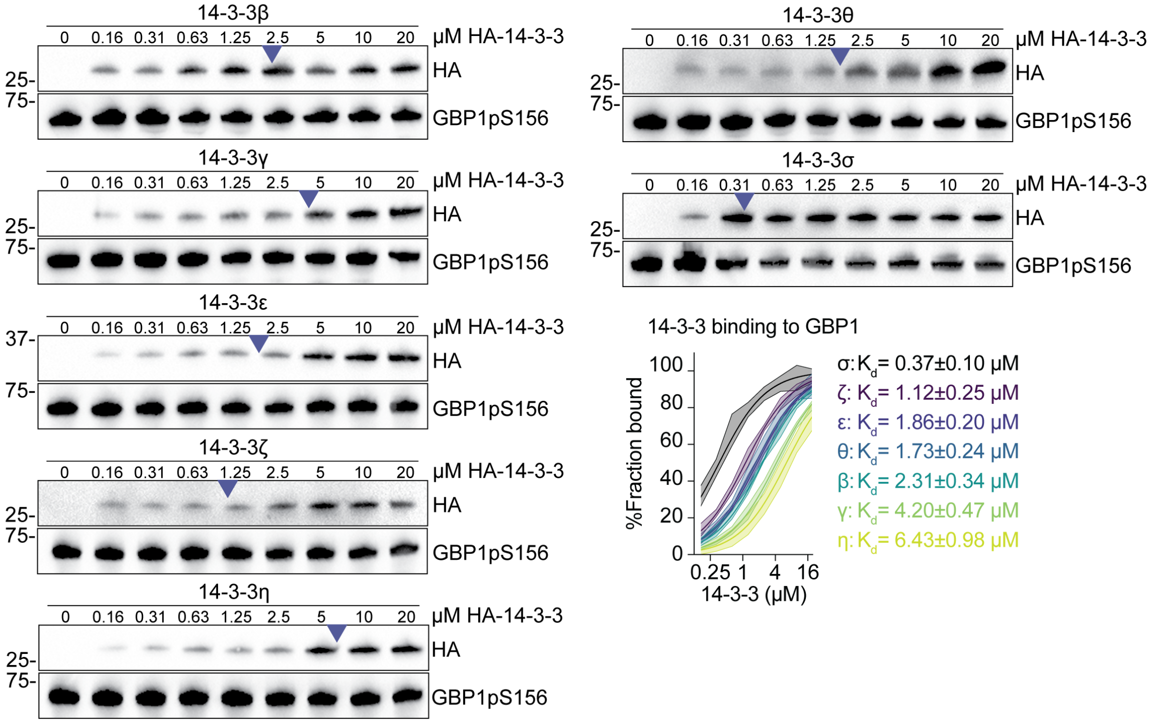


**Fig. S11: Determination of GBP1:14-3-3 protein interaction affinities.**
Immunoblots for semi-quantitative co-IP using immobilized GBP1pS156 to determine affinity of the respective 14-3-3 protein added in the indicated concentrations. Blue arrowheads mark approximate $K_d$ of the interaction. Graph showing result of semi-quantitative co-immunoprecipitations, plotting percentage of bound 14-3-3 protein to immobilized GBP1pS156 bait against added concentration of respective 14-3-3 protein.
**Data information:** Images and graph representative of n = 3 experiments. Graph shows mean ± SD (error bands). For gel source data, see **Data S5**.

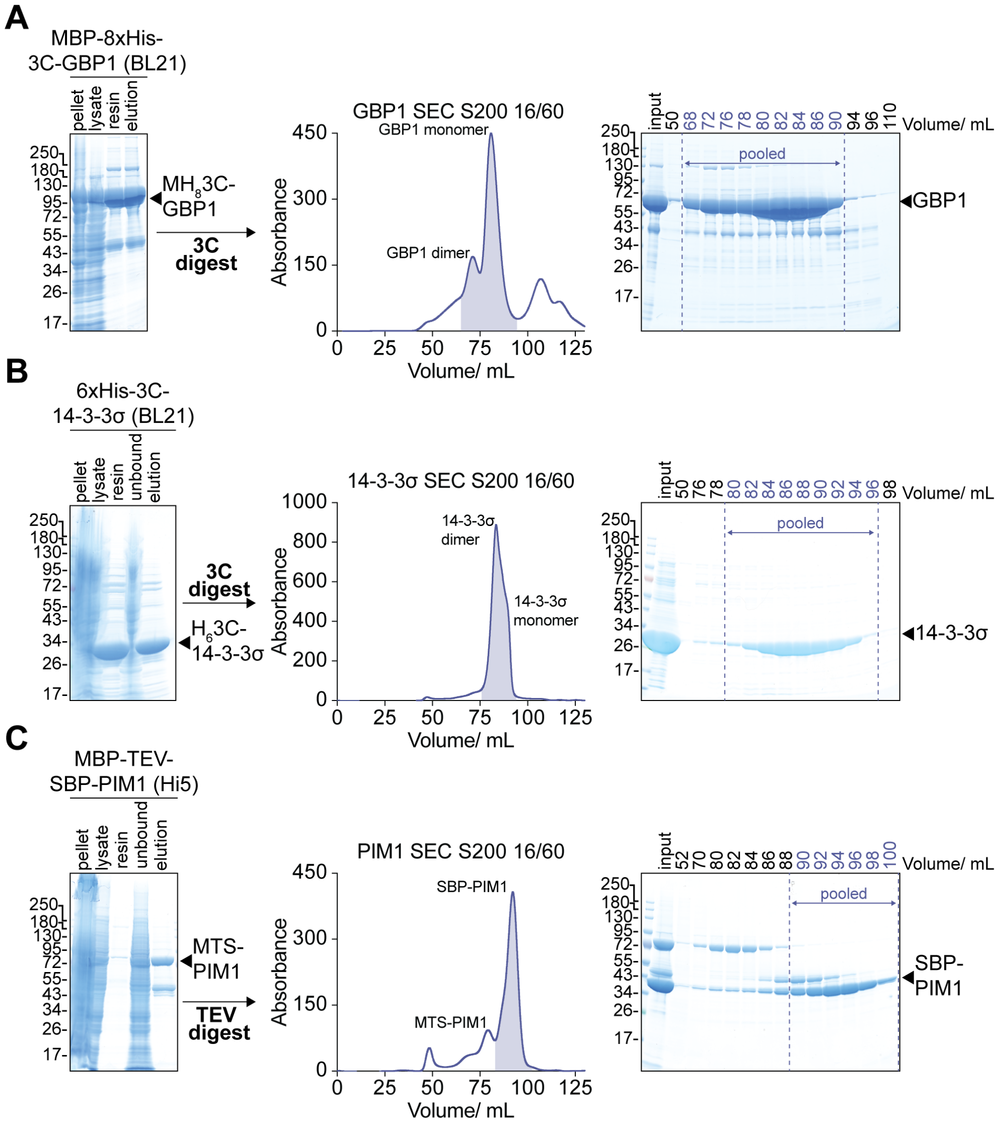


**Fig. S12: Production of recombinant human GBP1, 14-3-3σ and PIM1.**

Figure showing expression and purification of recombinant GBP1 **(A)**, 14-3-3σ **(B)** and PIM1 **(C)**. Coomassie stain of bulk purification using Ni-NTA affinity pulldown of MBP-8xHis-3C-GBP1 or 6xHis-3C-14-3-3σ from BL21 bacteria or amylose affinity purification of MBP-TEV-SBP (MTS)-PIM1 from Hi5 insect cells (left). Following digest with 3C- or TEV-protease, proteins were purified using size exclusion chromatography (SEC). Representative chromatograms and Coomassie-stained SDS-PAGE gels shown on the right, with indicated fractions that were pooled to obtain the purified proteins. For gel source data, see **Data S5**.

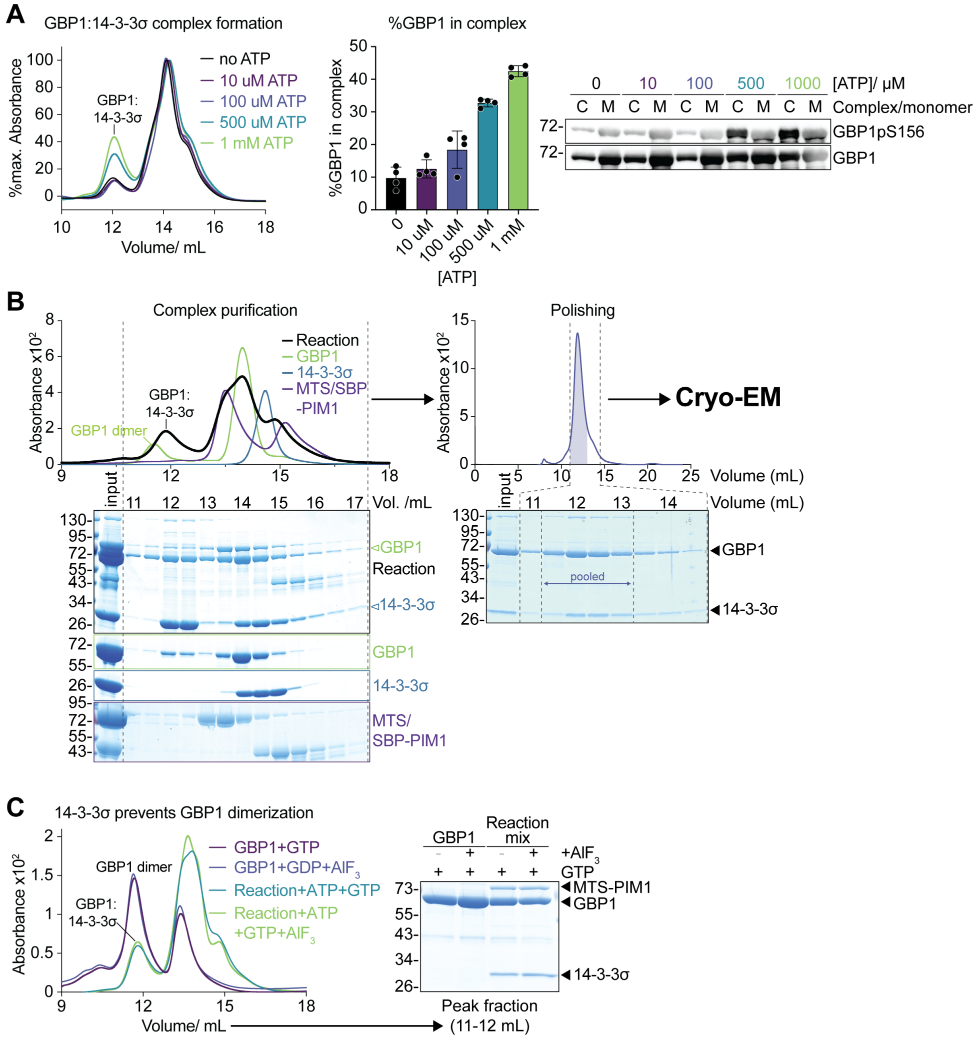


**Fig. S13: Biochemical characterization of the GBP1:14-3-3σ complex.**

**(A)** Size exclusion chromatography (SECs) of GBP1 phosphorylation by PIM1 and binding to 14-3-3σ (left). Peak quantification for GBP1:14-3-3σ complex and free GBP1 monomer, plotted as proportion of GBP1 contained within the complex (middle). Immunoblots for GBP1 phosphorylation in complex (C) or monomer (M) containing fractions from depicted chromatography. **(B)** Chromatograms and SDS-PAGE gels of indicated fractions of GBP1, PIM1 and 14-3-3σ alone or from an in vitro complex formation reaction (left). GBP1:14-3-3σ complex containing peak fractions were pooled and re-purified for polishing samples before structure determination by cryo-electron microscopy. Chromatogram and SDS-PAGE gel with indicated fractions used for structure determination (right). **(C)** SEC chromatograms and SDS-PAGE gels of peak fractions illustrating GBP1 dimerization. Samples contained GBP1 or GBP1+PIM1+14-3-3σ+ATP (Reaction mix) with addition of GTP, GDP+$AlF_3$. **Data information:** Chromatograms, graphs and blots shown in **(A)** representative of n = 4 and in **(B-C)** from n = 3 experiment. For gel source data, see **Data S5**.

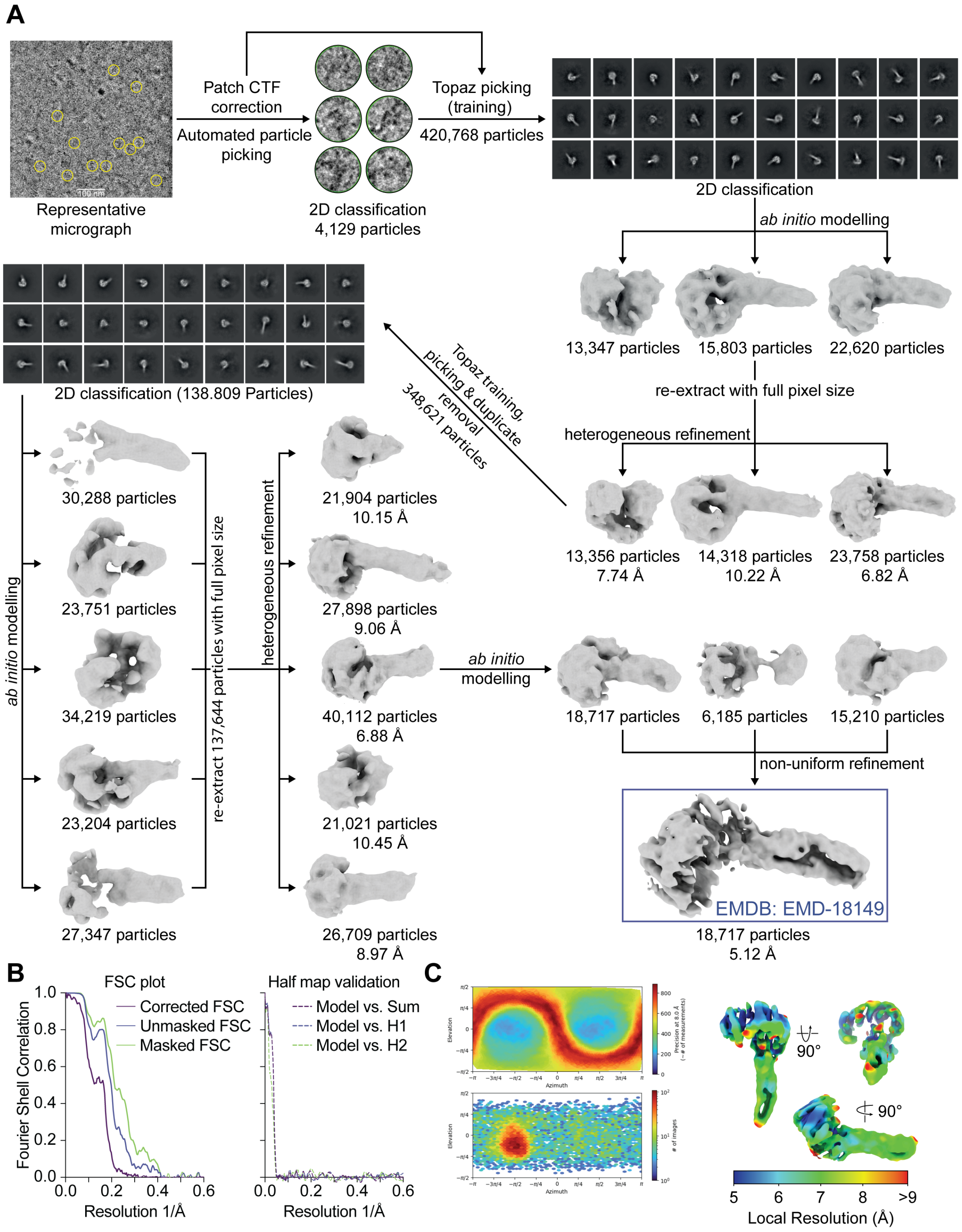


**Fig. S14: Supporting cryo-electron microscopy data.**

**(A)** Overview of cryo-EM data analysis strategy to obtain a 5.1 Å map of the trimeric GBP1:14-3-3σ complex. The final model was obtained using rigid body docking of previously described structures and without refining to the map. **(B)** Fourier shell correlation (FSC) and half map validation plots of the GBP1:14-3-3σ complex. **(C)** Angular distribution coverage (left) and EM density colored by local resolution (right).

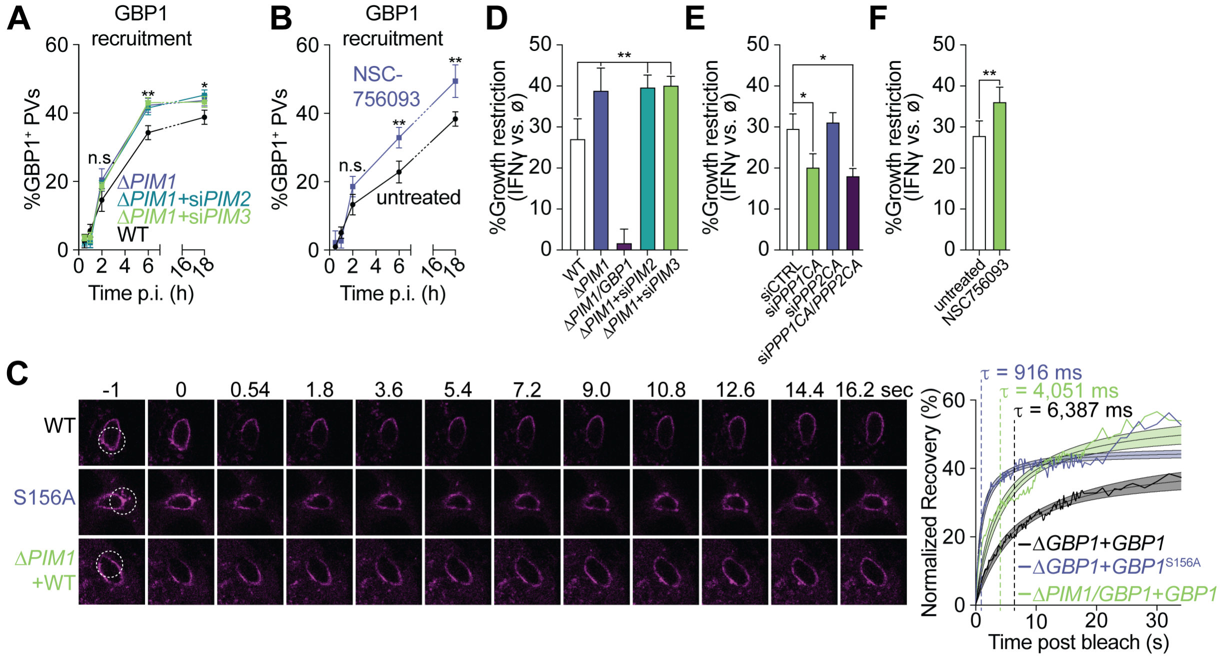


**Fig. S15: Effect of external factors on *Toxoplasma gondii* vacuole targeting and growth restriction by GBP1.**

**(A)** GBP1 recruitment quantification to type II *Toxoplasma gondii* (Tg) vacuoles at indicated times post infection in IFNγ-primed THP-1 WT or IFNγ+Dox-treated THP-1Δ*PIM1*/*GBP1*+mCH-*GBP1* cells transfected with siRNA for *PIM2* or *PIM3*. **(B)** mCH-GBP1 recruitment quantification to type II Tg vacuoles at indicated times post infection in IFNγ+Dox-primed THP-1Δ*GBP1*+mCH-*GBP1* cells treated with NSC756093. **(C)** Image sequence of fluorescence recovery after photobleaching (FRAP) experiments bleaching GBP1 on type II Tg vacuoles in IFNγ+Dox-primed cells at 2 hours p.i. Bleach area indicated by dotted circle. Graphs showing analysis of mCH-GBP1 fluorescence intensity to obtain recovery times ($\tau_{1/2}$) using hyperbolic curve. **(D-F)** Analysis of type II Tg-growth restriction at 18 hours p.i. in indicated cell lines treated with IFNγ or left untreated and transfected with siRNA for *PIM2* or *PIM3* **(D)**, transfected with indicated siRNAs **(E)** or treated with GBP1:PIM1 interaction inhibitor NSC756093 **(F)**. **Data information:** Graphs in **(A+B)** show mean ± SD from n = 3 experiments. Images in **(C)** representative of n = 10 bleached vacuoles per cell line. Graphs in **(C)** show mean and curve fit ± SD (error bands). Graphs in **(D-F)** show mean ± SD of n = 3 experiments plotted as relative growth restriction comparing to untreated cells. * *P ≤0.05*; ** *P ≤ 0.01*; *** *P ≤ 0.001* for comparison to WT cells in **(A)** or between untreated and NSC756093-treated cells in **(B)** and for indicated comparisons in **(D-F)** from 2-way ANOVA following adjustment for multiple comparisons; n.s. not significant.

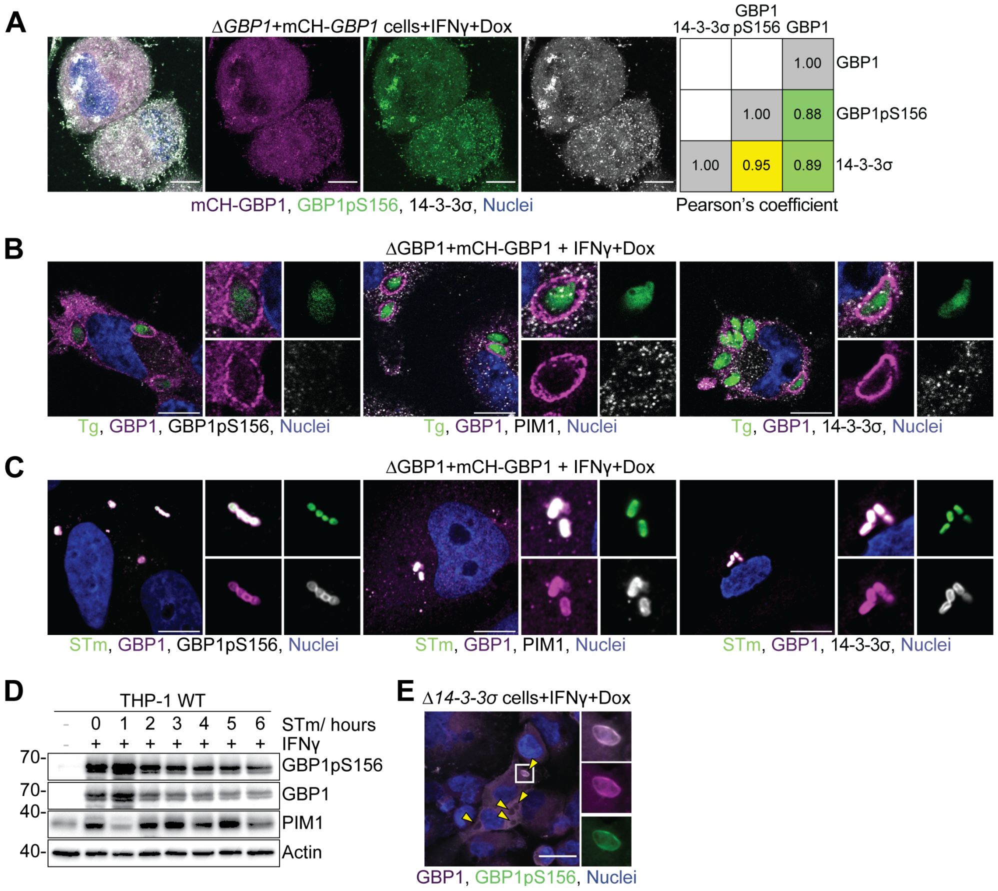


**Fig. S16: Protein localization and pathogen targeting by GBP1 upon infection.**
**(A)** Immunofluorescence images from IFNγ+Dox-treated THP-1Δ*GBP1*+mCH-*GBP1* cells stained for 14-3-3σ and GBP1pS156 and analysis of fluorescence signal colocalization analysis using Pearson's colocalization coefficient. Magenta: mCH-GBP1; Grey: 14-3-3σ; Green: GBP1pS156; Blue: nuclei. Scale bar, 10 µm. **(B)** Immunofluorescence images of IFNγ+Dox-treated THP-1 Δ*GBP1*+mCH-*GBP1* cells infected with type II *Toxoplasma gondii* (Tg) for 6 hours and stained for GBP1pS156, PIM1 or 14-3-3s. Magenta: mCH-GBP1; Grey: GBP1pS156/PIM1/14-3-3σ; Green: Tg; Blue: nuclei. Scale bar, 10 µm. **(C)** Immunofluorescence images of IFNγ+Dox-treated THP-1 Δ*GBP1*+mCH-*GBP1* cells infected with *Salmonella Typhimurium* (STm, MOI = 10) for 2 hours and stained for GBP1pS156, endogenous PIM1 or 14-3-3 σ. Magenta: mCH-GBP1; Grey: GBP1pS156/PIM1/14-3-3σ; Green: STm; Blue: nuclei. Scale bar, 10 µm. **(D)** Immunoblot analysis for GBP1 phosphorylation and PIM1 protein levels following STm infection (MOI = 10) of IFNγ-primed THP-1 WT cells at the indicated time points post infection. **(E)** Immunofluorescence images from IFNγ+Dox-treated THP-1Δ*14-3-3σ* cells infected with type II Tg for 6 hours and stained for GBP1 and GBP1pS156. Arrowheads: Tg position based on DNA stain. Magenta: GBP1; Green: GBP1pS156; Blue: nuclei. Scale bar, 20 µm. **Data information:** Images in **(A-E)** representative of n = 3 experiments. Heat map in **(A)** shows mean from n = 12 cells per condition imaged by 3D confocal microscopy. For gel source data, see **Data S5**.

## SUPPLEMENTARY TABLES

**Table S1: Cryo-EM data collection and processing.**

| | GBP1:14-3-3σ complex (EMDB: EMD-18149) |
|---|---|
| **Data collection and processing** | |
| Magnification | 215,000 |
| Voltage (kV) | 300 |
| Electron exposure (e–/Å$^2$) | 65.5 |
| Defocus range (μm) | -0.8 to -2 |
| Pixel size (Å) | 0.638 |
| Symmetry imposed | C1 |
| Initial particle images (no.) | 8,689,007 |
| Final particle images (no.) | 18,717 |
| Map resolution (Å)<br>FSC threshold | 5.12<br>0.143 |
| Map resolution range (Å) | 5.12 - 10 |

**Table S2: Overview of cell lines and *Toxoplasma gondii* parasite lines.**

| **Cell line** | **Source** |
|---|---|
| HEK293T | The Francis Crick Institute, Cell Services |
| HFF | The Francis Crick Institute, Cell Services |
| NIH3T3 | The Francis Crick Institute, Cell Services |
| Organoid 389 | Wanigasooriya *et al*, 2022 (52) |
| THP-1 WT | ATCC, TIB-202 |
| THP-1Δ*GBP1* | Fisch *et al*, 2019 (11) |
| THP-1Δ*GBP1*+Tet | Fisch *et al*, 2019 (11) |
| THP-1Δ*GBP1*+Tet-EV | Fisch *et al*, 2019 (11) |
| THP-1Δ*GBP1*+Tet-Flag-*GBP1*$^{K155A}$ | This study |
| THP-1ΔGBP1+Tet-Flag-*GBP1*$^{R151A}$ | This study |
| THP-1Δ*GBP1*+Tet-Flag-*GBP1*$^{R151A/K155A}$ | This study |
| THP-1Δ*GBP1*+Tet-Flag-*GBP1*$^{R151A/R153A}$ | This study |
| THP-1Δ*GBP1*+Tet-Flag-*GBP1*$^{R151A/R153A/K155A}$ | This study |
| THP-1Δ*GBP1*+Tet-Flag-*GBP1*$^{R153A}$ | This study |
| THP-1Δ*GBP1*+Tet-Flag-*GBP1*$^{R153A/K155A}$ | This study |
| THP-1Δ*GBP1*+Tet-Flag-*GBP1*$^{R153A/P158A}$ | This study |
| THP-1Δ*GBP1*+Tet-Flag-*GBP1*$^{S156A}$ | This study |
| THP-1Δ*GBP1*+Tet-Flag-*GBP1*$^{S156E}$ | This study |
| THP-1Δ*GBP1*+Tet-Flag-*GBP1* WT | Fisch *et al*, 2020 (13) |
| THP-1Δ*GBP1*+Tet-*GBP1* WT | Fisch *et al*, 2019 (11) |
| THP-1Δ*GBP1*+Tet-mCH-*GBP1*$^{R151A/R153A/K155A}$ | This study |
| THP-1Δ*GBP1*+Tet-mCH-*GBP1*$^{R153A/P158A}$ | This study |
| THP-1Δ*GBP1*+Tet-mCH-*GBP1*$^{S156A}$ | This study |
| THP-1Δ*GBP1*+Tet-mCH-*GBP1*$^{S156E}$ | This study |
| THP-1Δ*GBP1*+Tet-mCH-*GBP1*$^{S157A}$ | This study |
| THP-1Δ*GBP1*+Tet-mCH-*GBP1*$^{S157E}$ | This study |
| THP-1Δ*GBP1*+Tet-mCH-*GBP1* WT | Fisch *et al*, 2019 (11) |
| THP-1Δ*GBP1*+Tet-mCH-*GBP1*$^{Y427F}$ | This study |
| THP-1Δ*PIM1* | This study |

| | |
|---|---|
| THP-1Δ*PIM1*/*GBP1* | This study |
| THP-1Δ*PIM1*/*GBP1*+Tet | This study |
| THP-1Δ*PIM1*/*GBP1*+Tet-EV | This study |
| THP-1Δ*PIM1*+Tet | This study |
| THP-1Δ*PIM1*+Tet-EV | This study |
| THP-1Δ*PIM1*+Tet-*PIM1*$^{P81S}$ | This study |
| THP-1Δ*PIM1*+Tet-*PIM1* WT | This study |
| THP-1Δ*14-3-3σ* | This study |
| THP-1Δ*14-3-3σ*/*GBP1* | This study |
| THP-1Δ*14-3-3σ*/*GBP1*+Tet | This study |
| THP-1Δ*14-3-3σ*/*GBP1*+Tet-EV | This study |
| THP-1Δ*14-3-3σ*+Tet | This study |
| THP-1Δ*14-3-3σ*+Tet-EV | This study |
| THP-1Δ*14-3-3σ*+Tet-*14-3-3σ* | This study |
| **Parasite line** | **Source** |
| Type II (Prugniaud) Tg-GFP-Luc | Kim *et al*, 2007 (*81*) |
| Type II (Prugniaud) TgΔ*TgIST* | Moritz Treeck, The Francis Crick Institute |
| Type II (Prugniaud) TgΔ*Hpt* | Moritz Treeck, The Francis Crick Institute |
| Type II (Prugniaud) TgΔ*Hpt*+$GFP_{1-10}$ | Fisch *et al*, 2020 (*13*) |
| **Bacteria strain** | **Source** |
| *Salmonella Typhimurium* SL1344-GFP | Fisch *et al*, 2019 (*11*) |

**Table S3: List of siRNAs.**

| Target gene | Cat. number | Target gene | Cat. number |
|---|---|---|---|
| *PIM1* | L-003923 | *PPP1CA* | L-008927 |
| *PIM2* | L-005359 | *PPP2CA* | L-003598 |
| *PIM3* | L-032287 | *GBP1* | L-005153 |
| *AKT1/PKB* | L-003000 | *GBP2* | L-011867 |
| *RPS6KA1* | L-003025 | *GBP3* | L-031864 |
| *RPS6KB1* | L-003616 | *GBP4* | L-018177 |
| *ULK1* | L-005049 | *GBP5* | L-018178 |
| *MAPKAPK2* | L-003516 | Neg. CTRL | D-001810 |
| *MAPKAPK3* | L-005014 | | |

**Table S4: List of CRISPR guide RNA sequences**.

| Guide | Sequence 5' à 3' |
|---|---|
| *PIM1*-gRNA1 | GGAGGCCCGAGAGGAGTCGG |
| *PIM1*-gRNA2 | GTTCTGCTGAATGCCGCGAT |
| *14-3-3σ*-gRNA1 | ATGCCAGCCCCGAACAAGAG |
| *14-3-3σ*-gRNA2 | GGAGCAAGAACAGCGCCTAG |

**Table S5: List of qPCR primer sequences.**

| qPCR primer | Sequence 5' à 3' |
|---|---|
| *PIM1* | GCTCGGTCTACTCAGGCATC |
| | TACACTCGGGTCCCATCGAA |
| *PIM2* | TTCAGTGGGCTCAATCTGCG |
| | GACTGAGTCTGACAAGGGGG |
| *PIM3* | TACACCGACTTCGACGGCAC |
| | CTCCTCACAAGCTCTCGCTG |
| *AKT1* | TCCTGGTCCTGTCTTCCTCAT |
| | GTACTCCCCTCGTTTGTGCAG |
| *RPS6KA1* | AATGGACAGACCTCAGGGGA |
| | CCTCGGTCTGGAAGGCATAG |
| *RPS6KB1* | CCCTGTACGCATGCTCCTAC |
| | CCATGCCAAGTTCATATGGTCC |
| *ULK1* | GACCGCATTCACAGCATCAC |
| | AATGCACAGCTTGCACTTGG |
| *MAPKAPK2* | GTACATCCTGCTGTGTGGGTA |
| | ACAACCTGGTCAGCCAAGTG |
| *MAPKAPK3* | TCTTCCTAGAACCGCGGGC |
| | CCTCAGTTTTCTCACCCTGCC |
| *PPP1CA* | CCTGCTGGAAGTGCAGGG |
| | CCACGGAGCAGGAAGAAGTT |
| *PPP2CA* | CGAGTACTGCGGTGAGAGC |
| | CAGGATTTCTTTAGCCTTCTCGC |
| *YWHAB* | GGGCATGAACTCTCCAACGA |
| | ATGTCCTGCAGTTCTGCCTC |
| *YWHAG* | AAGAATTGCAGCGAGACCCA |
| | TTTGCTGATCTCGTGGGCTT |
| *YWHAE* | CACCTCATTCCAGCAGCTAACAC |
| | CAACCTGCAGGCACGGTC |
| *YWHAZ* | AGGAGCCCGTAGGTCATCTT |
| | GACTTTGCTCTCTGCTTGTGAAGC |
| *YWHAH* | CCGCTACTTAGCAGAGGTCG |
| | TGGCATCATCGAAGGCTTGT |
| *YWHAQ* | TGGCCTACAAGAACGTGGTC |
| | TTCGATCATCACCACACGCA |
| *YWHAS (14-3-3σ)* | TATAAGAACGTGGTGGGCGG |
| | GATGCGCTTCTTGTCGTCAC |
| *HPRT1* | ACCAGTCAACAGGGGACATAA |
| | CTTCGTGGGGTCCTTTTCACC |

**Table S6: List of primary antibodies.**

*IB: immunoblot, IF: immunofluorescence, IP: immunoprecipitation. Ms: mouse, Gt: goat, Rb: rabbit*

| primary Antibody | IB | IF | IP | | Source | RRID |
|---|---|---|---|---|---|---|
| 14-3-3σ | x | x | | Ms | ab14123, abcam | RRID:AB_300927 |
| β-actin | x | | | Gt | ab8229, abcam | RRID:AB_306374 |
| caspase-1 | x | | | Ms | Bally-1, Adipogen | RRID:AB_2490257 |
| cleaved caspase-3 | x | | | Rb | #9661, CST | RRID:AB_2341188 |
| E-cadherin | x | | | Rb | #3195, CST | RRID:AB_2291471 |
| Flag-M2 | x | | x | Ms | F3165, Sigma | RRID:AB_259529 |
| GAPDH | x | | | Rb | #2118, CST | RRID:AB_561053 |
| GBP1 (mAb) | x | x | x | Ms | Frickel lab | - |
| GBP1 (pAb) | x | | | Rb | Frickel lab | - |
| GBP1pS156 | x | x | x | Rb | Frickel lab | - |
| GM130 | | x | | Rb | ab52649, abcam | RRID:AB_880266 |
| HA | x | | x | Rb | #3724, CST | RRID:AB_1549585 |
| Histone H3 | x | | | Rb | #4499, CST | RRID:AB_10544537 |
| mCherry | x | | | Rb | ab167453, abcam | RRID:AB_2571870 |
| MLKL | x | | | Rb | #14993, CST | RRID:AB_2721822 |
| pan14-3-3 | x | | | Rb | #8312, CST | RRID:AB_10860606 |
| PIM1 | x | x | | Ms | ab54503, abcam | RRID:AB_882031 |
| PIM2 | x | | | Rb | #4730, CST | RRID:AB_2163921 |
| PIM3 | x | | | Rb | #4165, CST | RRID:AB_1904094 |
| pMLKL | x | | | Rb | #91689, CST | RRID:AB_2732034 |
| pro-caspase-3 | x | | | Rb | #9662, CST | RRID:AB_331439 |
| TgSAG1 | | x | | Ms | Frickel lab | - |

**DATA LEGENDS:**

**Data S1: MS/MS spectrum of GBP1-Ser156 and Thr590 phosphorylation (Separate Excel file).** Table showing experimental, and respective theoretical values of fragments in MS/MS spectrum of GBP1 peptide $L^{148}$THRIRSKpSSPDENE$^{162}$ containing the Ser156 phosphorylation site and $I^{576}$QDLQTKMRRRKACTIS$^{592}$ containing the Thr590 phosphorylation site, used for mapping of the sites to the GBP1 sequence. Extracted from Interactive Peptide Spectral Annotator.

**Data S2: Evolutionary analysis of GBP1 homologues (Separate Excel file).**
Table containing the information on the closest human GBP1 homologue from 484 species used for evolutionary analysis of occurrence of corresponding GBP1 Ser156 residue, the 14-3-3 binding motif and the PIM1 recognition motif.

**Data S3: GBP1 interactome in uninfected THP-1 cells (Separate Excel file).**
Table containing the mass spectrometry analysis for identification of GBP1 interacting proteins in IFNγ-primed THP-1 cells. Annotation: orange: significant interactor; green: GBP1; dark green: 14-3-3 proteins; red: common contaminant as identified using the CRAPome database.

**Data S4: GBP1 interactome in *Toxoplasma gondii*-infected THP-1 cells (Separate Excel file).**
Table containing the mass spectrometry analysis for identification of GBP1 interacting proteins in IFNγ-primed and type II *Toxoplasma gondii*-infected THP-1 cells. Annotation: orange: significant interactor upon infection, as compared to uninfected cells; green: highlighted significant hits; red: common contaminant as identified using the CRAPome database.

**Data S5: Uncropped gels and immunoblots (Separate pdf file).**
Uncropped immunoblots and gels.

**Data S6: Source data (Separate Excel file).**
Excel file containing all underlying source data.
